# Integrated metabolome and transcriptome analysis of castor oil accumulation during seed development in *Ricinus communis*

**DOI:** 10.3389/fpls.2026.1763593

**Published:** 2026-02-27

**Authors:** Xinxin Geng, Wenhua Tang, Maomao Ma, Chaoying Xu, Lijun Wang, Ying Wang, Mengling Huang, Yemei Sun, Liang Chen, Xiaomeng Xue

**Affiliations:** 1 Hubei Key Laboratory of Edible Wild Plants Conservation & Utilization, Hubei Normal University, Huangshi, China; 2 College of Life Sciences, Hubei Normal University, Huangshi, China; 3 Hubei Engineering Research Center of Special Wild Vegetables Breeding and Comprehensive Utilization Technology, Hubei Normal University, Huangshi, China; 4 Oil Crops Research Institute of the Chinese Academy of Agricultural Sciences/Key Laboratory of Biology and Genetic, Improvement of Oil Crops, Ministry of Agriculture, Wuhan, China

**Keywords:** castor oil accumulation, metabolome, ricinoleic acid, *Ricinus communis*, transcriptome

## Abstract

Ricinoleic acid is a high-value hydroxy fatty acid with broad industrial applications, which is the main composition of castor oil (approximately 87%). Elucidating the molecular mechanisms underlying seed oil synthesis and identifying key candidate genes are essential for increasing ricinoleic acid production. In this study, we employed an integrated metabolomic and transcriptomic approach to investigate the dynamic synthesis of castor oil during seed development in *Ricinus communis* (*R. communis*). A total of 790 structurally identified metabolites were detected across 5 distinct developmental stages. These differentially abundant metabolites (DAMs) were classified into 19 clusters based on their expression trends throughout seed development. Annotation results highlighted three key DAMs, i.e., hydroxy ricinoleic acid, ricinoleic acid methyl ester and ricinoleic acid, associated with ricinoleic acid, all of which exhibited a gradual increase in abundance as seed development progressed. Transcriptomic analysis identified 9,940 differentially expressed genes (DEGs), which were grouped into five distinct clusters. Among these, 30 enzyme genes related to fatty acid biosynthesis and six oil body-related genes were screened as candidate genes, with the majority predominantly enriched in clusters I and II. Co-expression network analysis of transcription factors (TFs) and oil-related genes in these two clusters further identified candidate key TFs potentially involved in oil synthesis, including *WRI1*, *DREB*, *LOB* and eight other families. Integrated metabolomic and transcriptomic analysis revealed that the co-upregulation of five candidate genes with three dominant accumulated metabolites (hydroxy ricinoleic acid, ricinoleic acid methyl ester and ricinoleic acid), including an enzyme gene (*FAH12*) directly involved in ricinoleic acid biosynthesis and four oil body-associated protein genes (*OLEs* and *PXG*) associated with oil accumulation and storage. Candidate alternative splicing events were identified during seed development, notably within lipid metabolism genes (*SCAD*, *LACS1*, *SBH2* and *LIP1P*) and transcription factor *ARF2*. This study provides a theoretical foundation and valuable reference for future high-oil breeding and metabolic engineering efforts aimed at optimizing ricinoleic acid production.

## 1 Introduction

*Ricinus communis* (*R. communis*, also known as castor) a species of the Euphorbiaceae family, produces seeds with a remarkably high oil content of 46-56%. Notably, only 2.2-2.5 kg of castor seeds are needed to yield one kg of oil, a conversion efficiency superior to that of most other oil crops. As the sole natural source of ricinoleic acid (Nitbani et al., 2022), castor seeds contain oil in which approximately 87% of the fatty acids are ricinoleic acid. In addition to its high ricinoleic acid content, castor exhibits exceptional agronomic traits, including drought tolerance, resistance to barren and saline soils, strong adaptability, and low input requirements, making it a high-efficiency source of valuable industrial feedstocks (Sánchez-Álvarez et al., 2022). On the other hand, the growing demand for non-freezing lubricants in the aviation industry has positioned castor as one of the top ten oil crops globally. Furthermore, ricinoleic acid can be chemically converted into petroleum-and diesel-like products, earning it the designation of “green oil”, with expanding applications in national defense, chemical manufacturing, pharmaceuticals, and other sectors. As a key renewable resource, ricinoleic acid has garnered worldwide attention (Melo et al., 2008). However, current castor oil production falls short of global market demand. Enhancing its cultivation and processing is therefore of strategic importance, particularly in developing countries facing resource shortages.

In recent years, high-throughput sequencing has revolutionized genetic and transcriptomic research, shifting from traditional Sanger sequencing to comprehensive genome-wide analyses that extend beyond individual genes or loci (Komarova et al., 2020). In plants, RNA transcriptomics has been widely applied in species such as *Arabidopsis*, rice, maize, *R. communis* (Hartmann et al., 2022; Waititu et al., 2021; Wang et al., 2016, 2019);, enabling the discovery of differentially expressed genes, highly expressed transcripts, novel genes, alternative splicing (AS) events, and SSR markers. Several transcriptomic studies in castor have provided valuable insights. For example, Chandrasekaran et al. (2014) profiled abscisic acid-mediated oil accumulation during seed development, while Wan et al. (2019) identified key genes involved in triacylglycerol (TAG) assembly and fatty acid synthesis, achieving a 20-fold increase in TAG accumulation in leaves via transient expression. These findings raise the possibility of producing oil in non-seeded tissues or microbial systems using castor caruncle genes. Wang et al. (2019a) combined Illumina short-read and PacBio long-read sequencing to reveal transcriptomic complexity and identify critical differentially expressed genes (DEGs) in the ricinoleic acid biosynthesis pathway across three developmental stages in high- and low-oil castor varieties. Similarly, Wu et al. (2021) reported 49 lipid biosynthesis-related DEGs in *R. communis*. Deng et al. (2025) investigated the effects of foliar-applied sodium selenite (Na_2_SeO_3_) on the physiological and molecular responses of castor (*Ricinus communis* L.) using transcriptomic analysis. Wu et al. (2025) focused on castor to investigate the molecular basis underlying leaf size variation using a synthetic autotetraploid by doubling the diploid homologous chromosomes via transcriptomic analysis.

Metabolomics, an emerging field focused on the comprehensive quantification and qualification of low-molecular-weight metabolites, seeks to link metabolic changes to physiological processes. In plants, widely-targeted metabolomics has been extensively used to investigate metabolite accumulation patterns, identify metabolic genes, and elucidate biosynthetic pathways. Profiling small molecules across developmental stages or under specific treatments to characterize metabolic dynamics has become a major focus in plant biology (Dettmer et al., 2007; Guo et al., 2019). For instance, Liu et al. (2020) compared secondary metabolites in yellowed and normal rice using a widely-targeted approach, while Li et al. (2021b) identified 672 metabolites and several differentially abundant metabolites (DAMs) across proso millet varieties with differing bran colors. Ma et al. (2022) uncovered region-specific metabolites in *Chaenomeles* sp*eciosa* through widely-targeted metabolomics. However, the biosynthesis of ricinoleic acid in castor bean (*Ricinus communis*) has not yet been elucidated through metabolomic approaches.

Multi-omics integration represents a powerful high-throughput strategy for uncovering regulatory networks in biological systems, offering deeper mechanistic insights. Integrated transcriptomic and metabolomic analysis, explores correlation between gene expression and metabolite accumulation, helping to construct coregulatory networks, pinpoint key candidate genes, and interpret phenotypic outcomes. Zhuang et al. (2019) unveiled anthocyanins accumulation and molecular analysis through metabolome and transcriptome in purple and green turnips. Wang et al. (2020) clarified the role of CoA in the salt tolerance by transcriptome and metabolome analysis in *Zygophyllum* spp. Zhou et al. (2020) identified several genes that involved in anthocyanin accumulation and coloration using metabolome and transcriptome analysis between white and pink tea flowers. Guo et al. (2019) revealed dynamic processes of flavonoid synthesis and purine metabolism by combing transcriptome and metabolome analysis during somatic embryogenesis transdifferentiation in cotton. Ying et al. (2019) provided fresh insights into fruit development and flesh coloration through transcriptomic and metabolomic profiling in a wild peach species *Prunus mira Koehn*e. Previously, a series of genes associated with lipid synthesis were identified in oil crops through combined transcriptome and metabolome analyses. Zhao et al. (2023) detected 98 lipid-related metabolites and key genes (e.g., *GmGAPDH* and *GmGPAT*) using transcriptomic and metabolomic approaches, thereby uncovering mechanisms underlying oil accumulation in soybean seeds. Xu et al. (2025) elucidated the mechanisms of oleic acid synthesis and identified key genes (e.g., *SAD* and *FabD*) in the mesocarp of seedless and tenera varieties via integrated transcriptome and metabolome analyses in oil palm. Although studies on metabolomic and combined omics during castor seed development remain limited, metabolomics can clarify the relationship between metabolite dynamics and physiological changes, while transcriptomics facilitates the identification of key genes involved in oil synthesis. Integrating both approaches provides a more accurate reconstruction of gene–metabolite networks, offering a robust strategy to decipher the molecular basis of castor oil accumulation.

To further enhance castor oil content and explore the underlying processes of ricinoleic acid biosynthesis, this study employs integrated metabolomic and transcriptomic analyses. By identifying key metabolites and DEGs associated with castor oil biosynthesis and incorporating them into a cohesive co-expression network, our work advances the molecular understanding of oil accumulation and expands the genetic toolkit for metabolic engineering aimed at optimizing ricinoleic acid production.

## 2 Materials and methods

### 2.1 Plant materials and growth conditions

From spring to autumn 2021, seeds of Youbi No.5 (*R. communis* line) were sown and planted under standard field conditions in Yangluo experimental field at OCRI, CAAS. The plant materials came from National Infrastructure for Crop Germplasm Resources of China. Seed samples were extracted from the fruits after removal of the pericarp for experimentation. Totals of 15 unmatured seed from 7 days after flowering (DAF) (S1), 14 DAF (S2), 21 DAF (S3), 28 DAF (S4) and 35 DAF (S5) (covering the whole development stages of seeds) were collected with three biological replicates, which were promptly frozen and stored at -80°C for transcriptome sequencing and metabolite profiling.

### 2.2 Metabolomics analysis and differentially accumulated metabolites screening

The protocols of metabolome profiling were performed according to Wang et al. (2019b) and Cao et al. (2019) and implemented by Wuhan MetWare Biotechnology Co., Ltd. (www.MetWare.cn). Briefly, three biological samples are freeze-dried and crushed with a zirconia bead for 1.5 min at 30 Hz. Then, 100mg of lyophilized powder was dissolved with 1.2 mL 70% methanol solution. Following centrifugation, the extracts were filtrated and then metabolome analysis was performed using UPLC/MS/MS system (SHIMADZU Nexera X2, Japan; Applied Biosystems 4500 Q-TRAP, USA) as described by Guo et al. (2019). The identification of metabolites was achieved by integrating multiple dimensions of analytical data, including the exact mass, MS/MS fragmentation patterns, isotopic distributions of MS/MS fragment ions, and retention time (RT). Specifically, a proprietary developed intelligent MS/MS spectrum-matching algorithm was employed to align the MS/MS spectra and RTs of metabolites detected in the project samples with those recorded in the in-house reference database (GB-PLANT). During the matching process, mass tolerance thresholds were set at 20 ppm for both MS and MS/MS data, and the RT tolerance was defined as 0.2 min. Multi reaction monitoring triple quadrupole mass spectrometry and peak area were used to quantify metabolites.

Principal component analysis (PCA) and correlation coefficients among the sample were determined using R software (v.4.5.2). Normalized metabolite data were used to compare all samples. Differentially accumulated metabolites (DAMs) between groups were determined by variable importance of projection (VIP) ≥ 1 and fold change > 1.5. VIP values were extracted from the OPLS-DA results generated using the R package (Metabo Analyst R) (v.1.0.1). The Metware database (MWDB) and other public databases were used to annotate metabolites. Metabolic pathways were subsequently annotated based on the Kyoto Encyclopedia of Genes and Genomes (KEGG) database.

### 2.3 Transcriptome and differential expression analysis

Following the standard protocol of Oxford Nanopore Technologies (ONT), total RNA was first extracted, assessed for purity and concentration, and only high-quality samples were used for transcriptome sequencing. Library construction was then performed, which involved primer annealing and reverse transcription to generate full-length cDNA with the addition of a switch oligo, followed by synthesis of the complementary strand. The resulting DNA underwent damage repair and end-repair was subsequently purified using magnetic beads. An ONT sequencing adapter was ligated using T4 DNA ligase (NEB). Finally, for sequencing, the prepared cDNA libraries were loaded onto FLO-MIN109 flow cells before running on a PromethION platform at Biomarker Technology Company (Beijing, China). The low-quality reads (length less than 500bp, quality score less than 6) and ribosomal RNA sequences were filtered out from raw sequences. Then, full-length non-chimeric (FLNC) transcripts based on the presence of primers at both ends of the sequence were obtained. FLNC transcripts were polished to obtain consensus isoforms before using minimap2 (Li, 2018) to remove redundancy based on the comparison results with the reference genome of *R. commons* (Chan et al., 2010). The final transcripts were ready for subsequent analysis.

The alternative splicing events in each sample were obtained through Astalavista software (Foissac and Sammeth, 2007), and the occurrence of the above five alternative splicing events in the transcripts was statistically analyzed according to Barbazuk et al. (2008) and Li et al. (2021a).

Based on Fold Change≥2 and FDR<0.01, DESeq2 (1.6.3) was performed for differential expression analysis. The *p*-value obtained from the null hypothesis test was adjusted using the Benjamini and Hochberg’s approach. Gene Ontology (GO, http://geneontology.org/), Non-redundant (Nr, http://ftp.ncbi.nih.gov/blast/db/FASTA/nr.gz), and Kyoto Encyclopedia of Genes and Genomes (KEGG, https://www.kegg.jp/) databases were used for gene annotation.

### 2.4 Quantitative real-time PCR analysis

Twelve overlapping DEGs (*28166.t000046, 29200.t000004, 29200.t000006, 29601.t000014, 29620.t000004, 29646.t000070, 29841.t000070, 29881.t000005, 29929.t000288, 30055.t000018, 30131.t000233, 30147.t000716, 30174.t000160, 30183.t000035, 60629.t00001, 60637.t00001*), 6 DEGs belong to Cluster I related to castor oil accumulation (*28035.t000007*, *27985.t000040*, *29673.t000033*, *29726.t000092*, *29682.t000014* and *27810.t000017*) and 5 candidate DEGs from correlation analysis of transcriptome and metabolome (*29794.t000071, 29917.t000061, 30147.t000162, 30008.t000036* and *28035.t000007*) were verified by qRT-PCR analysis. With a reverse transcriptase (Vazyme, Nanjing, China), total RNA was reverse transcribed to cDNA. QRT-PCR reaction was performed in ABI PRISM7500 sequencing system (Applied Biosystems, USA) with ChamQ Universal SYBR qPCR Master Mix (Vazyme, Nanjing, China). QRT-PCR reaction procedure was performed as follows: 95 °C for 5 minutes, then 40 cycles at 95 °C for 15 seconds, 60 °C for 15 seconds, and finally 72 °C for 30 seconds. All reactions were executed using one biological sample with four technical replicates. Aata analysis adopted the comparative Ct method according to Schmittgen and Livak (2008). *RcActin* gene was used as the internal standard (Cao et al., 2019). The qRT-PCR primers were designed using Primer 6.0 software (Premier, Canada) (**Supplementary Table S1**).

### 2.5 Co-expression analysis of TFs and lipid-related genes

Correlation analysis between the screened transcription factors (TFs) and lipid-related genes was conducted based on Pearson correlation coefficient using the built-in cor function in R software (v.4.5.2). The statistical significance of the correlation coefficients was determined by calculating the corresponding *p*-values with the corPvalueStudent function. Only the gene pairs meeting the stringent thresholds (coefficient of determination (*R²*) > 0.8 and *p*-value < 0.05) were retained. After removing duplicate gene pairs from the filtered results, the interaction network of the remaining gene pairs was visualized using Cytoscape software (v.3.10.4) (Shannon et al., 2003).

### 2.6 Comprehensive analysis of metabolome and transcriptome profiling

Correlation analysis between differential genes and differential metabolites were calculated based on Pearson’s correlation coefficients method. Before calculating the correlation, the z-value transformation method was used for data preprocessing, and then filtering was performed based on the correlation coefficient (r) and the *p*-value of the correlation. The filtering threshold was |r| > 0.80 and *p*-value < 0.05. The relationships between the metabolome and transcriptome data were illustrated using R software (v.4.5.2).

## 3 Results

### 3.1 Overall metabolite identification

To gain deeper insights into the metabolic changes during seed development in *R. communis*, we conducted metabolite profiling across these five stages (S1-S5). Three biological replicates of immature seeds from each stage (S1-S5) were subjected to metabolomic analysis. Principal component analysis (PCA) revealed a clear separation among the five distinct seed developmental stages (S1-S5) (**Supplementary Figure S1**). The results demonstrated high correlation coefficients among the sample replicates, indicating the reliability of the metabolomic data (**Supplementary Figure S2**). Following quality filtering, totals of 790 known metabolites were identified in S1 and S5. Among these, lipids (17.1%), Phenolic acid (15.9%), organic acids (12.8%), flavonoids (10.0%), and amino acids and derivatives (9.7%) accounted for a substantial proportion (**Supplementary Figure S3**). Detailed information on all identified metabolites, including ionization models, molecular weights, KEGG pathways, and the names, categories and quantities of compounds across the five seed developmental stages, was provided in **Supplementary Table S2**.

### 3.2 Differentially accumulated metabolites identification

**Figure 1:**
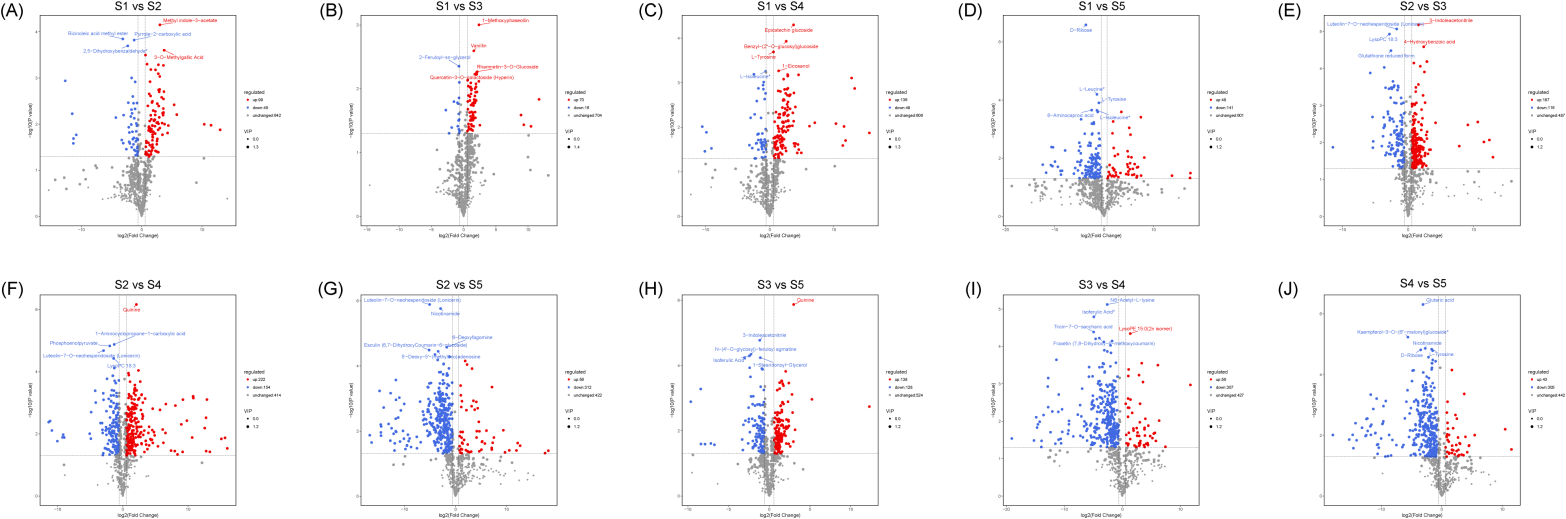
Volcano plot showing the differentially accumulated metabolites (DAM) among different group comparisons during seed development. **(A)** S1 vs S2, **(B)** S1 vs S3, **(C)** S1 vs S4, **(D)** S1 vs S5, **(E)** S2 vs S3, **(F)** S2 vs S4, **(G)** S2 vs S5, **(H)** S3 vs S4, **(I)** S3 vs S5, and **(j)** S4 vs S5.

Metabolomic profiling across five seed developmental stages (S1-S5) revealed dynamic changes in metabolite accumulation. A total of 10 pairwise comparisons were performed (**Figures 1A-J**; **Supplementary Figure S4**). The number of differentially accumulated metabolites (DAMs) varied among comparisons. Notably, stage S5 emerged as a critical phase for metabolic process, showing the highest number of common DAMs shared across comparisons involving this stage (**Supplementary Figure S5**). Three DAMs (isohyperoside, 1-O-(3,4-Dihydroxy-5-methoxy-benzoyl)-glucoside, and perillyl alcohol) were common to all ten comparison groups, suggesting their central roles throughout seed development.

### 3.3 KEGG enrichment analysis of DAMs and candidate metabolites identification

**Figure 2:**
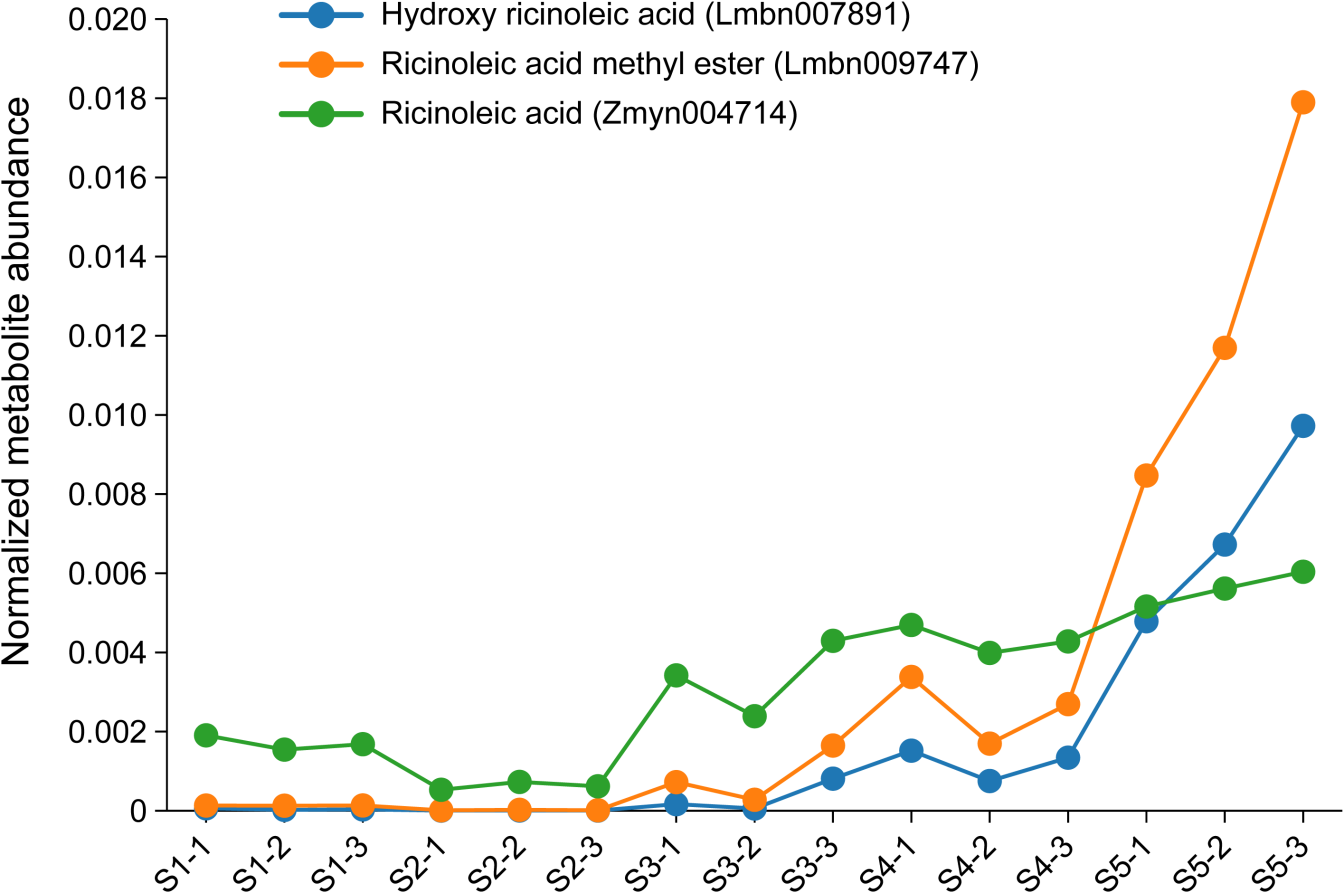
Normalized intensity of hydroxy ricinoleic acid , ricinoleic acid methyl ester, and ricinoleic acid at different developmental stages (S1–S5) of seeds. S1: 7 days after flowering (DAF), S2: 14 DAF, S3: 21 DAF, S4: 28 DAF, S5: 35 DAF. S1-1, S1-2, and S1-3 represent three biological replicates for stage S1, and the same applies to other stages.

All DAMs were categorized into 19 expression trend clusters via K-means clustering (**Supplementary Figure S6A-S**). KEGG enrichment analysis of DAMs indicated that metabolic shifts during seed development were prominently associated with amino acid biosynthesis, phenylpropanoid metabolism, and oxidative carboxylic acid metabolism across multiple comparisons (**Supplementary Figure S7**; **Supplementary Tables S3-S6**). Focusing on lipids relevant to castor oil composition, we identified several DAMs corresponding to major fatty acids, including hydroxy ricinoleic acid (Lmbn007891), ricinoleic acid methyl ester (Lmbn009747), ricinoleic acid (Zmyn004714), stearic acid (mws1489), palmitic acid (mws1488) and linoleic acid (mws1491). Notably, three ricinoleic acid-related metabolites (Lmbn007891, Lmbn009747, and Zmyn004714), based on MS/MS spectral matching, clustered together (subcluster 16) and exhibited a progressive increase in abundance from stage S1 to S5 (**Figure 2**; **Supplementary Figure S6P**), aligning with the active phase of oil accumulation during late seed maturation.

### 3.4 Transcriptome profiling and differentially expressed genes (DEGs) identification

To identify candidate factors associated with oil accumulation in castor seeds, a total of 15 RNA-seq libraries were constructed from immature seeds collected at five developmental stages (S1-S5). High-quality sequencing data were obtained with mean read lengths spanning 845 to 1,555 bp across all samples. Clean reads showing high alignment rates (96.84-98.62%) were successfully aligned to the *R. communis* reference genome (**Supplementary Table S7**). A total of 32,194 genes were annotated for subsequent analysis. Differential expression analysis identified 9,940 genes that were differentially expressed in at least one pairwise comparison between stages (**Supplementary Table S8**). The greatest number of differentially expressed genes (DEGs) was observed in comparisons involving the earliest stage (S1), indicating a major shift in gene expression during initial development. In contrast, fewer DEGs were detected between the later stages (e.g., S3-S5), suggesting increased transcriptional stability as maturation progresses.

To characterize the expression dynamics of DEGs throughout seed development, K-means clustering was performed on the 9,940 DEGs based on log_2_(FPKM + 1) values, using a similarity regulation model combined with hierarchical clustering. The DEGs were partitioned into five distinct clusters according to their expression trends. Cluster I (1,127 DEGs) exhibited a continuous upward trend from S1 to S5 (**Supplementary Figure S8A**). Cluster II (2,640 DEGs) displayed a bell-shaped profile, increasing from S1 to S3 and subsequently declining through S5 (**Supplementary Figure S8B**). Cluster III (1,519 DEGs) showed an initial decrease from S1 to S2, followed by stable expression in later stages (**Supplementary Figure S8C**). Cluster IV (2,865 DEGs) demonstrated a consistent down-ward trend across all stages (**Supplementary Figure S8D**). Finally, Cluster V (1,789 DEGs) exhibited a fluctuating pattern, characterized by a decline from S1 to S2, stability from S2 to S3, an increase from S3 to S4, and a final decrease from S4 to S5 (**Supplementary Figure S8E**).

### 3.5 Detection of alternative splicing events

**Figure 3:**
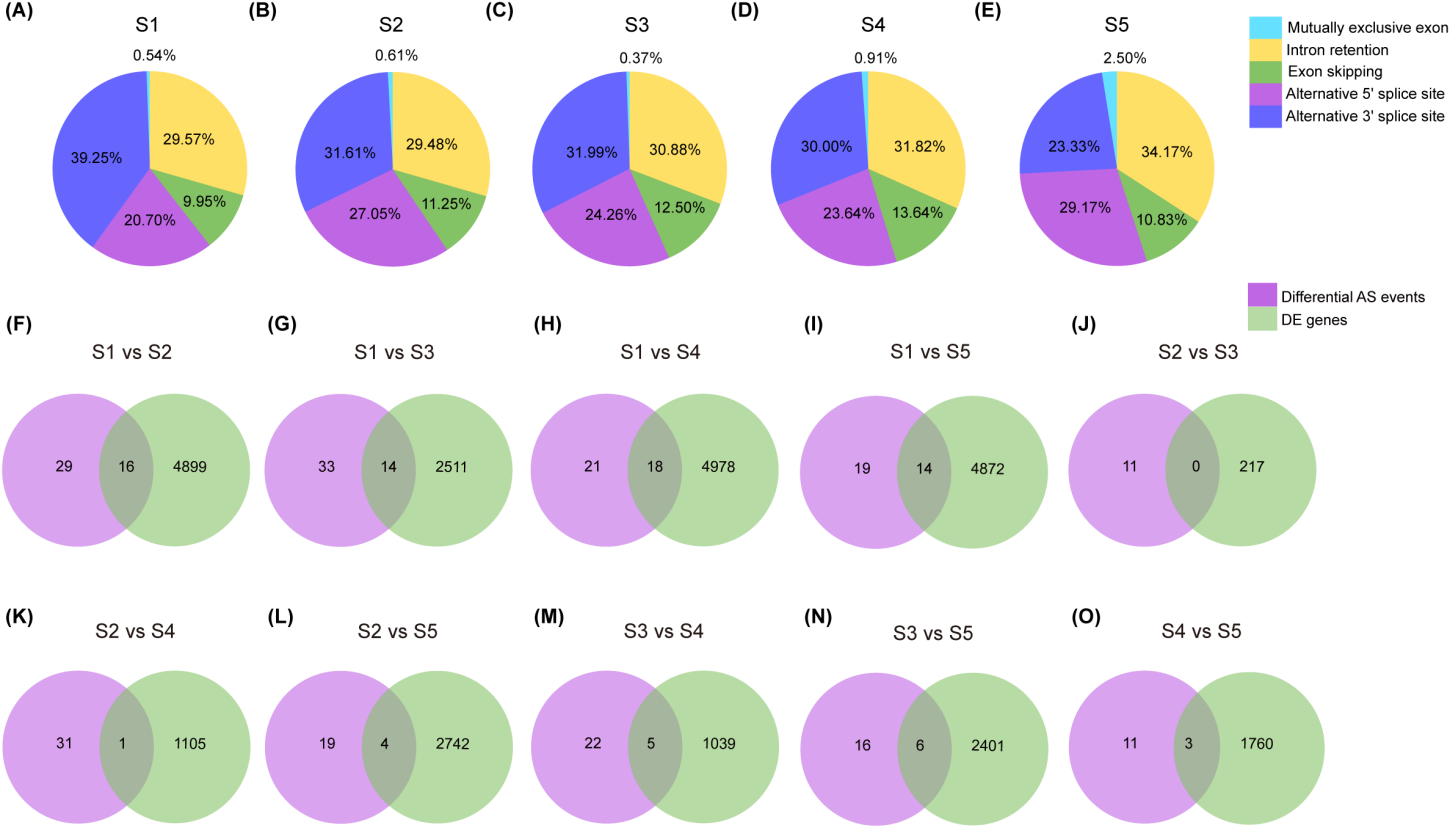
Alternative splicing landscapes among five different seed developing stages samples (S1-S5) and comparison between differentially expressed (DE) genes and differentially AS genes. **(A-E)** Frequency of each AS types in sample of S1, S2, S3, S4 and S5, respectively. **(F-O)** Venn diagram showed number of DE genes associated with differential AS genes in the comparison of S1 vs S2, S1 vs S3, S1 vs S4, S1 vs S5, S2 vs S3, S2 vs S4, S2 vs S5, S3 vs S4, S3 vs S5 and S4 vs S5, respectively.

Based on Oxford Nanopore Technologies (ONT) sequencing data, a total of 3,556 alternative splicing (AS) events were identified during seed development from S1 to S5. Intron retention (IR; 1,235 events) and alternative 3’splice sites (A3SS; 1,054 events) were the most common types, followed by exon skipping (ES; 625 events), alternative 5’splice sites (A5SS; 622 events), and mutually exclusive exons (MEE; 20 events). The number of AS events was highest at stage S1 and lowest at S5, indicating dynamic AS regulation during seed development The distribution of AS types varied across developmental stages (**Figures 3A-E**). IR, A3SS, and ES consistently represented most events in S1-S3. In S4, A3SS and A5SS proportions increased slightly, while IR and ES decreased. In S5, the profile shifted markedly: A3SS became the most abundant type (34.17%), followed by ES (29.17%), while IR declined to 23.33%. MEE also increased to 2.5% in S5 but remained below 1% in earlier stages. These shifts suggest that specific splicing patterns are associated with different phases of seed development, and phenotypic differences between S5 and the other four stages may be partially influenced by shifts in alternative splicing profiles.

We further investigated differentially expressed alternative splicing (DAS) events by overlapping DEGs and different AS events across stages S1-S5. Most DAS events occurred in comparisons involving S1 (**Figures 3F-I**), with fewer events detected between later stages (**Figures 3J-O**). This indicates that splicing-mediated regulatory changes are most active during the transition from S1 to S2, whereas later development (S3-S5) is characterized by relative splicing stability. Functional annotation of DAS genes highlighted several genes associated with lipid metabolism and hormone response. Specifically, *SCAD* (*30032.t000002*) was identified in the S1 vs S2 comparison; *LACS1* (*30076.t000180*) in S1 vs S3; *SBH2* (*29813.t000027*) and *LIP1P* (*29804.t000062*) in S1 vs S4; and *ARF2* (*29647.t000040*) in S3 vs S5 (**Supplementary Table S9**). These findings suggest that alternative splicing plays an important role during seed development by generating distinct isoforms in a stage-specific manner.

### 3.6 Candidate genes identification

**Figure 4:**
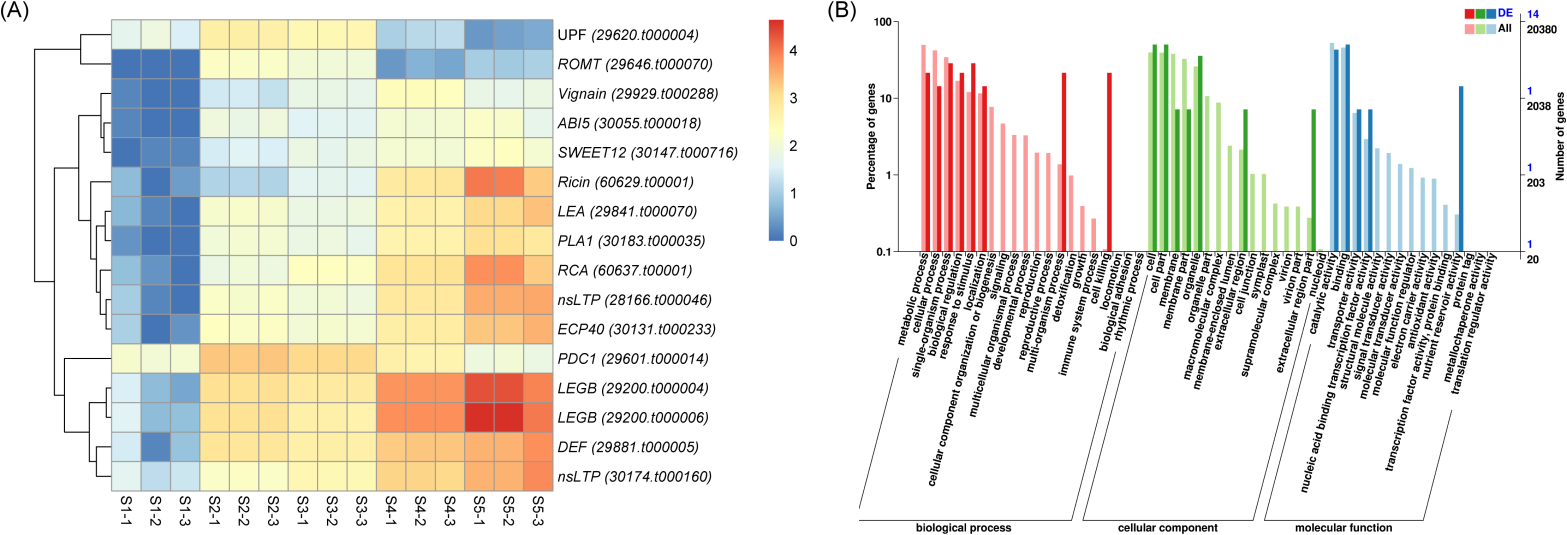
Expression patterns **(A)** and GO term analysis **(B)** of the 16 overlapping DEGs among 10 comparison groups. The heatmap presents normalized Log_2_FPKM expression values.

To identify candidate genes potentially involved in castor oil accumulation, we intersected DEGs across multiple seed developmental stages to pinpoint common DEGs shared among ten comparative groups. This analysis revealed only 16 overlapping DEGs exhibiting distinct expression profiles (**Table 1**). Among these, the expression of five genes, *29200.t000006*, *29620.t000004*, *30174.t000160*, *60637.t00001* and *60629.t00001*, increased gradually during seed development (**Figure 4A**), suggesting that they might play a significant role in seed development. Functional annotation indicated that these genes encode proteins including glutelin type-A 3 precursor (LEGB, 29200.t000006), non-specific lipid-transfer protein A-like (nsLTP, 30174.t000160; 29620.t000004), ricin-like (Ricin, 60629.t00001) and agglutinin (RCA, 60637.t00001). GO enrichment analysis further highlighted their predominant involvement in metabolic processes (**Figure 4B**). The expression patterns of eight randomly selected common DEGs were validated by qRT-PCR, confirming consistency with the RNA-seq data (**Supplementary Figure S9**).

**Table 1:** Functional annotation of the 16 overlapping genes DEGs among different seed development groups.

Gene ID	Gene name	Function description	KEGG pathway
*28166.t000046*	nsLTP	Non-specific lipid-transfer protein	NA
*29200.t000004*	LEGB	Legumin B precursor	NA
*29200.t000006*	LEGB	Legumin B	NA
*29601.t000014*	PDC1	Pyruvate decarboxylase 2	Glycolysis/Gluconeogenesis
*29620.t000004*	UPF	Uncharacterized proteinfamily	NA
*29646.t000070*	ROMT	Trans-resveratrol di-O-methyltransferase	NA
*29841.t000070*	LEA	Late embryogenesis abundant protein D-7	NA
*29881.t000005*	DEF	Defensin-like protein	NA
*29929.t000288*	Vignain	Vignain-like	NA
*30055.t000018*	ABI5	Abscisic acid-insensitive 5	Plant hormone signal transduction
*30131.t000233*	ECP40	embryogenic cell protein 40	NA
*30147.t000716*	SWEET12	Bidirectional sugar transporter SWEET12	NA
*30174.t000160*	nsLTP	Non-specific lipid-transfer protein A-like	NA
*30183.t000035*	PLA1	Phospholipase A1-Igamma2	NA
*60629.t00001*	Ricin	Ricin-like	NA
*60637.t00001*	RCA	Agglutinin	NA

**Figure 5:**
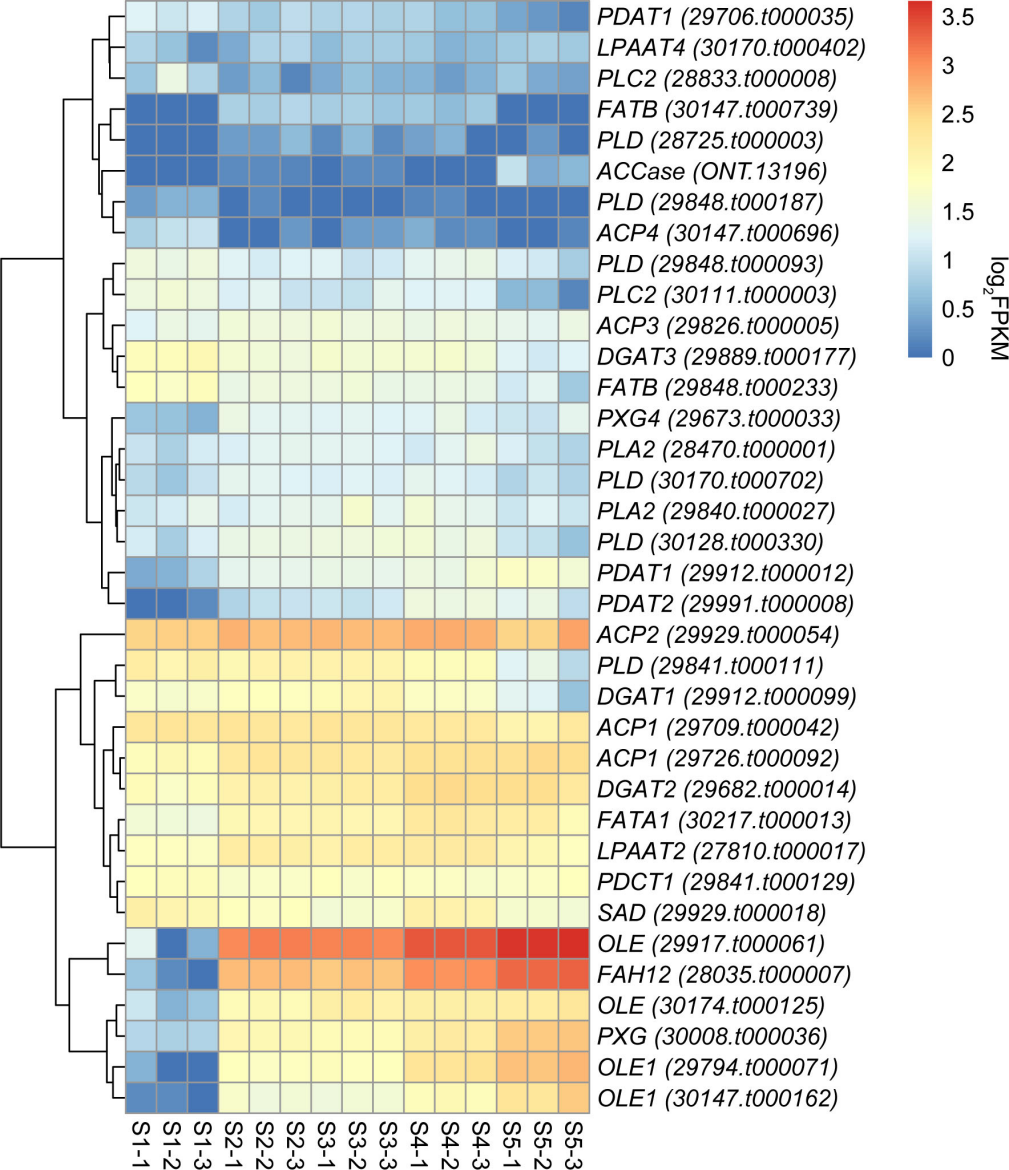
Heatmap illustrating the Log_2_FPKM of 30 key enzyme genes related to ricinoleic acid biosynthesis and six oil body-related genes at five time points (S1-S5) during seed development.

Key enzymes involved in castor oil biosynthesis, such as ACBP, ACCase, ACP, ACS, DGAT, EAR, FATA, FATB, FAH12, GPAT, KAS, KAR, LPAAT, LPCAT, PAP, PDCT, PDAT, PLA2, PLC2, PLD, SAD, as well as oil body-related proteins (OLE and PXG), were surveyed among the DEGs. A total of 30 genes potentially encoding enzymes associated with ricinoleic acid biosynthesis and six oil body-related genes were identified (**Table 2**). Based on their expression trends across five developmental stages, these genes were categorized into 5 clusters (**Table 2**; **Supplementary Table S6**). Thirteen genes, including *OLEs*, *PDATs*, *PXGs*, *ACP1*, *ACCase*, *FATA1*, *FAH12* and *DGAT2*, were assigned to Cluster I, showing a continuously rising expression pattern, consistent with the accumulation pattern of three ricinoleic acid-related metabolites (hydroxy ricinoleic acid, ricinoleic acid methyl ester and ricinoleic acid). Ten genes, such as *ACPs*, *FATB*, *PLA2*, *LPAAT2* and *PLDs*, fell into Cluster II, exhibiting a bell-shaped expression profile. The remaining genes were distributed among Cluster III (three genes: *PDCT1*, *LPAAT4*, and *PLD*), Cluster IV (six genes: *PDAT1*, *ACP4*, *DGAT1*, *DGAT3*, *PLC2*, and *PLD*), and Cluster V (four genes: *SAD*, *PLD*, *PLC2*, and *FAFB*), all displaying declining expression trends. Expression heatmap analysis of the 30 key enzyme and six oil body-related genes (**Figure 5**) revealed that one gene encoding ACP2 (*29929.t000054*) maintained relatively high expression throughout five seed development stages, implying a sustained role in oil synthesis. Additionally, seven genes including *ACP1* (*29709.t000042*), *DGAT2* (*29682.t000014*), *ACP1* (*29726.t000092*), *FATA1* (*30217.t000013*), *LPAAT2* (*27810.t000017*), *PDCT* (*29841.t000129*) and *SAD* (*29929.t000018*), exhibited moderate expression across all stages, suggesting their involvement in maintaining oil biosynthesis. In contrast, eight genes, including *PDAT1* (*29706.t000035*), *LPAAT4* (*30170.t000402*), *PLC2* (*28833.t000008*), *FATB* (*30147.t000739*), *PLD* (*28725.t000003*), *ACCase* (*ONT.13196*), *PLD* (*29848.t000187*), and *ACP4* (*230147.t000696*), showed consistently low expression, indicating limited contribution to oil synthesis during the observed stages.

**Table 2:** Clusters of the 30 DEGs encoding key enzymes related to ricinoleic acid biosynthesis and six oil body-related proteins among different seed development groups.

Subcluster	Gene ID	Enzyme	Oil body-related protein	Subcluster	Gene ID	Enzyme	Subcluster	Gene ID	Enzyme
Cluster 1	*29794.t000071*	–	OLE1	Cluster 2	*29709.t000042*	ACP1	Cluster 4	*29706.t000035*	PDAT1
	*29917.t000061*	–	OLE		*29826.t000005*	ACP3		*29841.t000111*	PLD
	*30147.t000162*	–	OLE1		*29929.t000054*	ACP2		*28833.t000008*	PLC2
	*30174.t000125*	–	OLE		*30147.t000739*	FATB		*29912.t000099*	DGAT1
	*29912.t000012*	PDAT1	–		*28470.t000001*	PLA2		*29889.t000177*	DGAT3
	*29991.t000008*	PDAT2	–		*29840.t000027*	PLA2		*30147.t000696*	ACP4
	*29673.t000033*	–	PXG4		*27810.t000017*	LPAAT2	Cluster 5	*29929.t000018*	SAD
	*30008.t000036*	–	PXG		*30128.t000330*	PLD		*29848.t000093*	PLD
	*29726.t000092*	ACP1	–		*28725.t000003*	PLD		*30111.t000003*	PLC2
	*ONT.13196*	ACCase	–		*30170.t000702*	PLD		*29848.t000233*	FATB
	*30217.t000013*	FATA1	–	Cluster 3	*29848.t000187*	PLD			
	*28035.t000007*	FAH12	–		*30170.t000402*	LPAAT4			
	*29682.t000014*	DGAT2	–		*29841.t000129*	PDCT1			

To verify transcriptome reliability, five randomly selected DEGs from Cluster I were subjected to qRT-PCR. Their expression trends aligned well with the RNA-seq data (**Supplementary Figure S10**), supporting the robustness of our transcriptomic findings.

### 3.7 Identification of core transcript factors related to lipid regulation

**Figure 6:**
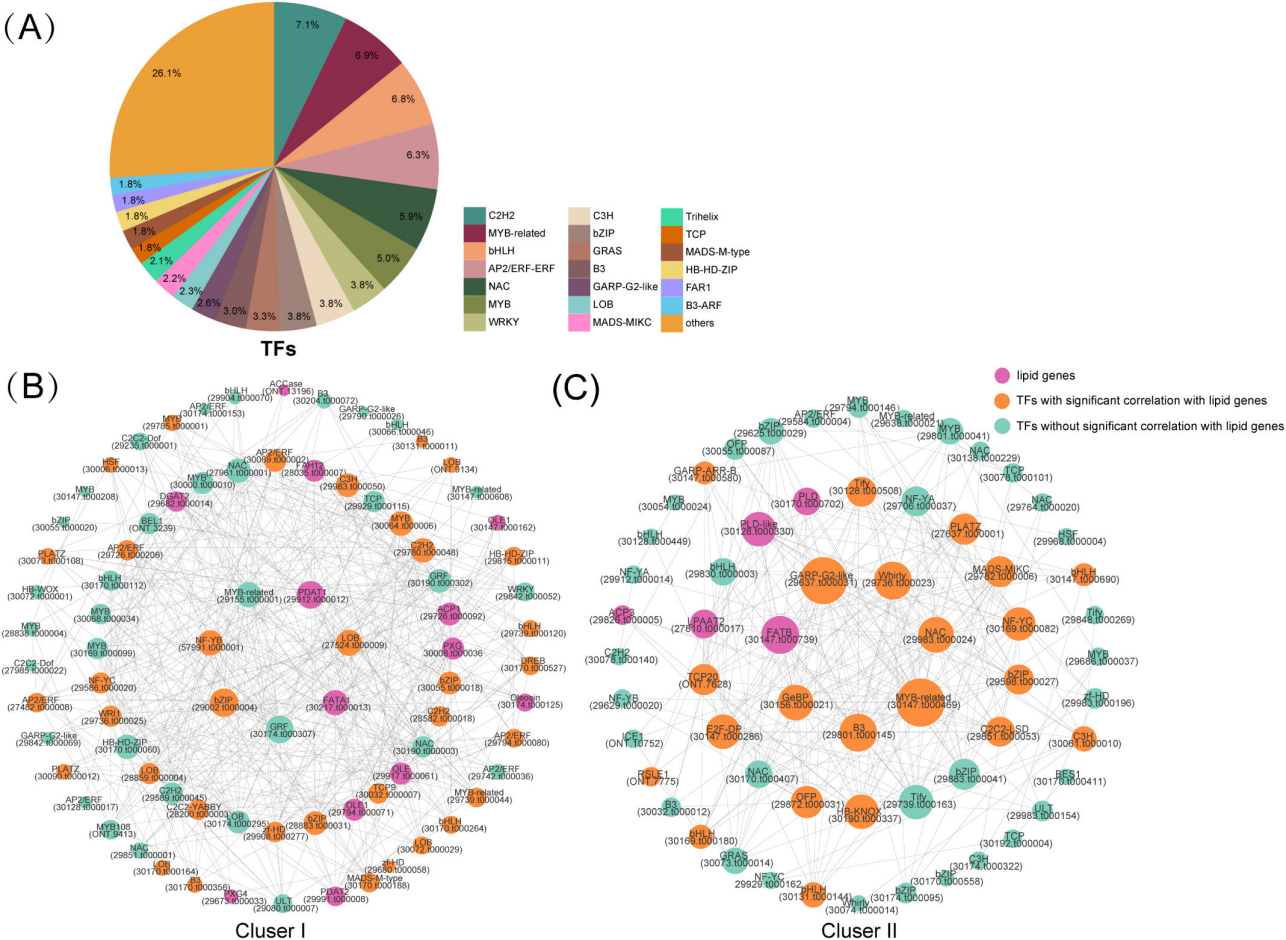
Network analysis and identification of key TFs related to lipid synthesis. **(A)** Statistical classification of predicted transcription factors. Gene co-expression networks of TFs and lipid-related genes in cluster I **(B)** and cluster II **(C)**.

Transcription factors (TFs), encoded by an extensive gene family in plants, serve as core regulators of gene expression. In this study, a total of 1,701 genes belonging to 69 TF families were identified through alignment of annotated transcripts against the AGRIS database (**Supplementary Table S11**). The top 20 TF families, each containing more than 30 genes, included C2H2, MYB-related, bHLH, AP2/ERF-ERF, NAC, MYB, WRKY, C3H, bZIP, GRAS, B3, GARP-G2-like, LOB, MADS-MIKC, Trihelix, TCP, MADS-M-type, HB-HD-ZIP, FAR1 and B3-ARF (**Figure 6A**). Enrichment analysis of DEG expression patterns demonstrated that a substantial number of lipid metabolism-related DEGs were predominantly clustered in clusters I and II (**Supplementary Table S9**). To further identify key TFs involved in lipid synthesis, we performed co-expression analyses between lipid-related genes and TFs within clusters I and II (**Figures 6B, C**). The results showed that 35 TFs, categorized into 17 distinct types, were identified in cluster I. Among these, the AP2/ERF family was the most abundant with 6 members, followed by the LOB family (5 members), MYB family (4 members), and bZIP family (3 members) (**Supplementary Table S12**). Notably, the AP2/ERF (e.g., *WRI1*, *DREB*) and LOB family members exhibited co-expression with multiple lipid-related genes in cluster I, such as *FAH12*, *PDAT2*, *ACP1*, *FATA1*, identifying them as candidate genes for functional validation in the oil accumulation process of castor seeds (**Figure 6C**; **Supplementary Table S12**). A total of 24 TFs, categorized into 20 distinct types, were identified in cluster II (**Supplementary Table S12**). Among these, only bHLH and Tify families contained three and two members, respectively (**Supplementary Table S12**). PLATZ, GARP-G2-like, MYB-related, GeBP and GRAS exhibited co-expression with multiple lipid-related genes in cluster II, such as *FATB*, *LPAAT2*, *PLD* (**Figure 6C**; **Supplementary Table S12**), indicating their potential important roles in lipid metabolism. Collectively, the identification of specific TF families, such as AP2/ERF and LOB in cluster I and PLATZ, GARP-G2-like, and others in cluster II, as co-expressed with lipid metabolism genes highlights them as strong candidate genes implicated in oil accumulation.

### 3.8 The integrated analysis of genes and metabolites related to ricinoleic acid biosynthesis

Pearson correlation analysis revealed 266 very strong (|r| > 0.9) and 338 strong (0.8 < |r| ≤ 0.9) correlation events between the top 100 DEGs and the top 50 DAMs (*p*-value < 0.05; **Supplementary Figure S11**). Co-enrichment analysis identified 9 KEGG pathways shared by transcripts and metabolites during seed development, including: biosynthesis of unsaturated fatty acids (ko01040), isoquinoline alkaloid biosynthesis (ko00950), tropane, piperidine and pyridine alkaloid biosynthesis (ko00960), phenylpropanoid biosynthesis (ko00940), ubiquinone and other terpenoid-quinone biosynthesis (ko00130), tyrosine metabolism (ko00350), phenylalanine, tyrosine and tryptophan biosynthesis (ko00400), phenylalanine metabolism (ko00360), and biosynthesis of amino acids (ko01230).

Furthermore, to examine the associations between 9,940 DEGs and 3 key DAMs, Pearson correlation analysis identified 1,982, 1,913, and 395 DEGs strongly correlated (0.8 < |r| ≤ 0.9, *p*-value < 0.05) with hydroxy ricinoleic acid (Lmbn007891), ricinoleic acid methyl ester (Lmbn009747) and ricinoleic acid (Zmyn004714), respectively (**Supplementary Table S13**). Notably, only six of these correlated DEGs were annotated as key enzymes involved in lipid biosynthesis or oil body-related proteins. Specifically, two DEGs (*FAH12* and *PLD*) were key enzymes for ricinoleic acid biosynthesis and four DEGs *(OLE1*, *OLE1*, *OLE* and *PXG*) encoded as oil body-related proteins (**Table 3**). Among these, *PLD* (*29841.t000111)* was classified into Cluster IV, and its expression level shows a decreasing trend. Notably, five DEGs (*OLE1*: *29794.t000071*, *OLE*: *29917.t000061*, *OLE1*: *30147.t000162*, *PXG*: *30008.t000036* and FAH12: *28035.t000007*) were assigned to Cluster I, exhibiting an upward expression trend consistent with the accumulation patterns of the three key DAMs (**Figure 5**). The expression patterns of the five candidate genes were validated by qRT-PCR, showing strong agreement with the RNA-seq data (**Supplementary Figure S10**). *FAH12* is a key gene influencing ricinoleic acid synthesis during seed development (Chen et al., 2007). Our findings provide further evidence confirming this earlier report. Oleosins (OLEs) and caleosins (PXGs) play a crucial role in encapsulating and stabilizing lipid droplets during triacylglycerol assembly (Badami and Kudari, 1970; Hanano et al., 2006). Collectively, these integrated transcriptomic and metabolomic findings highlight the essential role of further substantiate the pivotal function of *FAH12* in ricinoleic acid synthesis and oil body protein-related genes (OLEs and PXGs) in oil stabilization and storage during castor oil accumulation process in *Ricinus communis*.

**Table 3:** Functional annotation of the six candidate DEGs through the integrated analysis of genes and metabolites.

ID	Gene	KEGG pathway	Zmyn004714	Lmbn007891	Lmbn009747
			CC	CC	CC
*29794.t000071*	OLE1	--	0.83451099	0.93596709	0.93796082
*29917.t000061*	OLE	--	0.8088826	0.87567617	0.8779444
*30147.t000162*	OLE1	--	--	0.96708872	0.97194431
*30008.t000036*	PXG	Cutin, suberine and wax biosynthesis (ko00073)	--	0.93002331	0.92539821
*28035.t000007*	FAH12	Biosynthesis of unsaturated fatty acids (ko01040); Fatty acid metabolism (ko01212)	0.80009779	0.88849103	0.88919069
*29841.t000111*	PLD	Glycerophospholipid metabolism (ko00564); Ether lipid metabolism (ko00565); Endocytosis (ko04144)	--	-0.8797404	-0.8788608

## 4 Discussion

*R. communis*, a species belonging to the Euphorbiaceae family, produces seeds with an oil content exceeding 50% (Xu et al., 2016). Due to its high oil yield, it is ranked among the top ten oil crops globally (Brown et al., 2012). Castor oil serves as a key raw material for high-grade lubricants. Moreover, ricinoleic acid, often referred to as “green oil”, is widely utilized in national defense, chemical and pharmaceutical industries, and particularly in the aerospace sector as a high-performance lubricant. These attributes have drawn increasing research attention to the castor oil industry and the biosynthesis of ricinoleic acid. While previous transcriptomic studies partially elucidated the biosynthetic pathway of ricinoleic acid (Wang et al., 2019a; Wu et al., 2021), transcriptomics alone offers limited insights into the dynamic molecular mechanisms underlying its biosynthesis. In recent years, integrated multi-omics approaches have been extensively employed to uncover the biosynthetic mechanisms of various metabolites and secondary metabolites, such as lipids, anthocyanins, triterpenoids, and flavonoids, enabling a more comprehensive understanding of metabolite accumulation and transcriptional regulation in plants (Xie et al., 2025; You et al., 2024; Zheng et al., 2022; Xue et al., 2022; Guo et al., 2020; Zhuang et al., 2019). To gain deeper insights into the processes governing castor oil accumulation and ricinoleic acid biosynthesis, we performed a combined metabolomic and transcriptomic analysis across 5 distinct stages of seed development in *R. communis*, with the aim of constructing dynamic molecular profiles throughout this period.

### 4.1 Accumulation of DAMs associated with castor oil in *R. communis*

Metabolite profiling across different developmental stages identified several differentially accumulated metabolites (DAMs) associated with key fatty acids: including hydroxy ricinoleic acid, ricinoleic acid methyl ester, ricinoleic acid, stearic acid, palmitic acid and linoleic acid. Three ricinoleic acid-related DAMs (hydroxy ricinoleic acid, ricinoleic acid methyl ester and ricinoleic acid) exhibited a progressive increase from stages S1 to S5, indicating significant changes in ricinoleic acid content during seed development. Expression analysis of DAMs corresponding to stearic, palmitic, and linoleic acids revealed a declining trend for mws1489, whereas mws1488 and mws1491 showed upward trends (**Supplementary Table S3**). These metabolomic findings are consistent with previous reports. Zhang et al. (2016b) observed that oleic, linoleic, and ricinoleic acid levels increased steadily during castor seed development, aligning with the upward trends of mws1488 and mws1491 in our study. Furthermore, Chen et al. (2007) and Wu et al. (2021) reported that ricinoleic acid was undetectable in early developmental stages but accumulated substantially during mid-to-late stages, peaking at maturity. This pattern corroborates the expression profiles of the three ricinoleic acid-related DAMs identified in our metabolome analysis (**Figure 2**).

### 4.2 Revealed candidate genes involved in castor oil accumulation through integrated metabolomic and transcriptomic analysis in *R. communis*

Transcriptomic analysis of developing castor seeds enabled the identification of key genes involved in castor oil accumulation. Wu et al. (2021) identified 10 key candidate genes (including *OLEs*, *PXG*, *LACS7*, *PLA2*, *ACCase*, *DGAT*, and *PDAT*) that exhibited increasing expression patterns during seed development. Similarly, Tian et al. (2019) reported 12 ricinoleic acid-related genes in *Hiptage benghalensis*, further supporting the functional importance of *OLE*, *PXG*, *ACCase*, *DGAT*, and *PDAT* in ricinoleic acid accumulation. In this study, transcriptomic analysis identified 30 genes encoding key enzymes in the ricinoleic acid biosynthesis pathway and six oil body-related proteins involved in castor oil accumulation and storage (**Table 2**; **Supplementary Table S10**). Among these, 13 genes, including *OLEs*, *PDATs*, *PXGs*, *ACP1*, *ACCase*, *FATA1*, *FAH12*, and *DGAT2*, were grouped into Cluster I, characterized by an upward expression trend during seed development (**Table 2**). Notably, approximately 85% of these genes, such as *OLEs*, *PDATs*, *PXGs*, *FAH12*, and *DGAT2*, function in the later stages of castor oil synthesis and accumulation. Their rising expression paralleled the increasing ricinoleic acid content. This co-occurrence suggests these genes are implicated in two key processes: ricinoleic acid biosynthesis (as enzymes) and castor oil accumulation/storage (as oil body-associated proteins). Additionally, the Cluster II genes with a “bell-shaped” expression trend also played a crucial role in the accumulation of ricinoleic acid in castor oil. Ten genes, including *ACPs*, *FATB*, *PLA2*, *LPAAT2* and *PLDs*, were classified into this cluster (**Supplementary Table S10**).

#### 4.2.1 Candidate enzyme genes directly involved in ricinoleic acid biosynthesis

Oleate 12-hydroxylase (FAH12) serves as the key enzyme in the biosynthesis of ricinoleic acid in castor oil, catalyzing the conversion of 2-oleoyl-PC to 2-ricinoleoyl-PC (Chan et al., 2010). van de Loo et al. (1995) demonstrated that *FAH12* expression was highly abundant in castor seeds but remains minimal in stems, leaves, and other organs. Heterologous expression of *FAH12* in model plants such as *Arabidopsis* and *Camelina sativa* has been shown to significantly increase hydroxy fatty acid (HFA) accumulation in seeds (Broun and Somerville, 1997; Lu et al., 2006). Furthermore, co-expression with *PDAT1A*, *FAH12* enhanced HFA accumulation, reaching about 27% (van Erp et al., 2011). In the present study, *FAH12* (*28035.t000007*) exhibited a progressive increase from developmental stage S1 to S5 (**Table 2**; **Supplementary Table S10**), consistent with its role in castor oil biosynthesis. This expression pattern aligns with earlier reports of its upregulation during seed development in castor (Chen et al., 2007). Importantly, integrated transcriptomic and metabolomic correlation analysis in this study revealed that *FAH12* expression strongly correlate with several ricinoleate-associated metabolites, further supporting its central role in the hydroxy fatty acid synthesis pathway. In summary, *FAH12* is critically important for maintaining high ricinoleic acid synthesis in developing castor seeds.

DGAT, a key enzyme in the Kennedy pathway for triacylglycerol assembly, is critical for ricinoleic acid synthesis (Xu et al., 2018; Cagliari et al., 2010; Troncoso-Ponce et al., 2011). In *Physaria fendleri*, *DGAT1*, *DGAT2*, and *PDAT2* promote hydroxy fatty acid enrichment (Horn et al., 2016), whereas *DGAT2* and *PDAT2* are highly expressed during ricinoleic acid biosynthesis in *H. benghalensis* (Tian et al., 2019). Notably, we identified the same *DGAT2* gene (*29682.t000014*) previously reported by Wang et al. (2019a). ACCase, localized in the cytoplasm, generates malonyl-CoA for fatty acid synthesis (Sasaki et al., 2004). Its overexpression enhances fatty acid content in oil crops (Roesler et al., 1997). Here, a newly annotated *ACCase* gene (*ONT.13196*) exhibited a rising expression pattern from S1 to S5 (**Table 2**; **Supplementary Table S10**).

Plant phospholipases catalyze the hydrolysis of membrane phospholipids and are classified by their cleavage sites: PLA, PLC, and PLD. PLA releases fatty acids and lysophospholipids by cleaving the sn-1 or sn-2 positions of glycerophospholipids. PLC produces DAG by hydrolyzing the bond on the glycerol side, while PLD yields PA by cleaving the bond near the head group (Ali et al., 2022). PLA2 was reported to help accumulate a high level of the hydroxy fatty acid ricinoleate in TAGs (Chen et al., 2015). Wu et al. (2021) identified a PLA2 gene (*30142.t000005*) that showed a high expression level in the later stage of castor bean seed development. In our study, two PLA2s and three PLDs were found to exhibit a bell-shaped expression pattern that rose first and then fell (**Table 2**; **Supplementary Table S10**). The expression peaks of these PLA2 and PLD genes temporally coincided with the key stage of ricinoleic acid accumulation, implying their potential roles in fine-tuning the supply of precursors or signaling molecules for castor oil biosynthesis.

LPAAT is a key rate-limiting enzyme in the triacylglycerol (TAG) assembly pathway, primarily involved in plant oil synthesis within the endoplasmic reticulum. Overexpression of the *LPAAT* gene in *Arabidopsis thaliana*, *Brassica napus*, and tobacco has been found to increase seed oil content by 8% to 48% (Zou et al., 1997; Wang et al., 2017; Shams et al., 2025). Here, the expression of *LPAAT2* (*27810.t000017*) rose first and then fell, which likely represented the metabolic switch from membrane lipid synthesis to storage lipid synthesis (**Table 2**; **Supplementary Table S10**). As a member of AAT enzyme family, the two main types of FATs in plants (FATA and FATB) demonstrate high specificity for oleoyl-ACP substrate (Sujatha, 2009). Sánchez-García et al. (2010) suggested that the high catalytic efficiency of FATA and the affinity of FATB for oleoyl-ACP promote ricinoleic acid accumulation in *R. communis*. Consistently, in this study, *FATA1* (*30217.t000013*) showed gradually increasing expression and *FATB* (*30147.t000739*) displayed a “bell-shaped” trend from S1 to S5 (**Table 2**; **Supplementary Table S10**). In the process of fatty acid synthesis, acyl carrier protein (ACP) carries acyl chains to complete enzymatic reactions such as condensation, reduction, and dehydrogenation in plastid. Kim and Chen (2015) identified *PfACP5* as the most highly expressed *ACP* isoform in lesquerella, while Wu et al. (2021) reported a bell-shaped expression pattern for the same *ACP1* gene (*29726.t000092*) from S1 to S3.

Overall, our expression profiling reveals a coordinated transcriptional program of key genes (including *FAH12*, *FATs*, *ACPs*, *ACCase*, *LAPPT*, *PDAT*, *PLs* and *DGAT2*), providing novel insights into the process of ricinoleic acid accumulation.

#### 4.2.2 Proteins associated with oil body formation and lipid storage

Oleosins (OLEs), steroleosins and caleosins (PXGs) are key oil-body-associated proteins that stabilize lipid droplets during triacylglycerol assembly in developing seeds (Badami and Kudari, 1970; Beaudoin and Napier, 2002; Murphy, 2005). In this study, four *OLE* genes (*29917.t000061, 29794.t000071*, *30147.t000162* and *30174.t000125*) exhibited up-regulated expression, with OLE (*29917.t000061*) showing the highest transcript levels, suggesting a principal role in seed development, while the other three likely support later ricinoleic acid accumulation. Integrated transcriptomic and metabolomic analysis revealed strong correlations (|r| > 0.8, *p*-value <0.05) between three OLE genes (*29917.t000061*, *29794.t000071* and *30147.t000162*) (**Table 2**; **Supplementary Table S10**) and three ricinoleate-related metabolites, aligning with prior reports of their involvement in castor oil body formation and lipid storage (Wu et al., 2021; Tian et al., 2019; Hanano et al., 2006). Meanwhile, these proteins have been implicated in oil body formation not only in *R. communis* (Troncoso-Ponce et al., 2011) but also in *Physaria fendleri* (Horn et al., 2016) and *H. benghalensis* (Tian et al., 2019). Additionally, two caleosin (*PXG*) genes (*29673.t000033* and *30008.t000036*) in this study were grouped into Cluster I and displayed up-regulated expression from stages S1 to S5. Among them, *30008.t000036* exhibited a strong correlation (|r| > 0.8, *p*-value < 0.05) with three key ricinoleate metabolites. These findings reinforce the functional importance of both *OLE* and *PXG* genes in oil body stability and castor oil accumulation, consistent with earlier studies in *R. communis* and related species (Wu et al., 2021).

Taken together, it underscores the essential roles of *OLEs* and *PXGs* in the sequestration and stability of ricinoleic acid during seed development in *R. communis.*

#### 4.2.3 Transcription factors

TFs are the master switches controlling gene expression in plants. They govern essential processes like development and stress response by activating or repressing target genes. Meanwhile, previous studies in *R. communis* have identified a series of key transcription factors regulating lipid metabolism. For instance, *WRI1* was identified as crucial for triacylglycerol (TAG) storage in developing seeds (Tajima et al., 2013), while other TFs, including bHLH, MYB, and NAC families, were shown to govern ricinoleic acid metabolism (Brown et al., 2012). These established roles are consistent with the findings of our present study. In our study, a total of 1,701 genes from 69 TF families were identified (**Supplementary Table S11**). Consistently, a transcriptomic analysis of castor bean seeds by Wang et al. (2019a) also identified 69 TF families, which indicates that the repertoire of transcription factors is relatively conserved in *R communis*. Members of the AP2/ERF, LOB, MYB, and bZIP families showed significant co-expression with multiple lipid metabolism genes, particularly key lipid regulators in the AP2/ERF family such as *WRI1* and *DREB*. *WRI1* has been confirmed in various oil crops as an upstream regulator of core fatty acid synthesis genes (Kim et al., 2016; Kong et al., 2020; Zhang et al., 2016a), and our findings support *WRI1* as a strong candidate gene in playing a conserved and central role in lipid accumulation in castor. Moreover, research on the LOB family primarily focuses on disease resistance and plant lateral organ development, while its function in castor oil lipid biosynthesis remains incompletely elucidated (Jia et al., 2017; Guo et al., 2016). In this study, multiple members of this family were co-expressed with lipid metabolism genes, suggesting that the LOB family may play an important role during specific stages of seed development that is associated with lipid synthesis processes. Collectively, our analysis suggest that multiple transcription factor families may be involved in the lipid accumulation process in castor seeds. In particular, *AP2/ERF* and *LOB* families, as well as several novel families, are prioritized for their potential association with the lipid synthesis pathway, though their functional roles require validation. These findings lay the groundwork for a deeper understanding of the transcriptional network associated with lipid synthesis in castor seeds and provide a set of candidate genes for improving lipid yield and quality in castor seeds and other oil crops through genetic engineering approaches.

**Figure 7:**
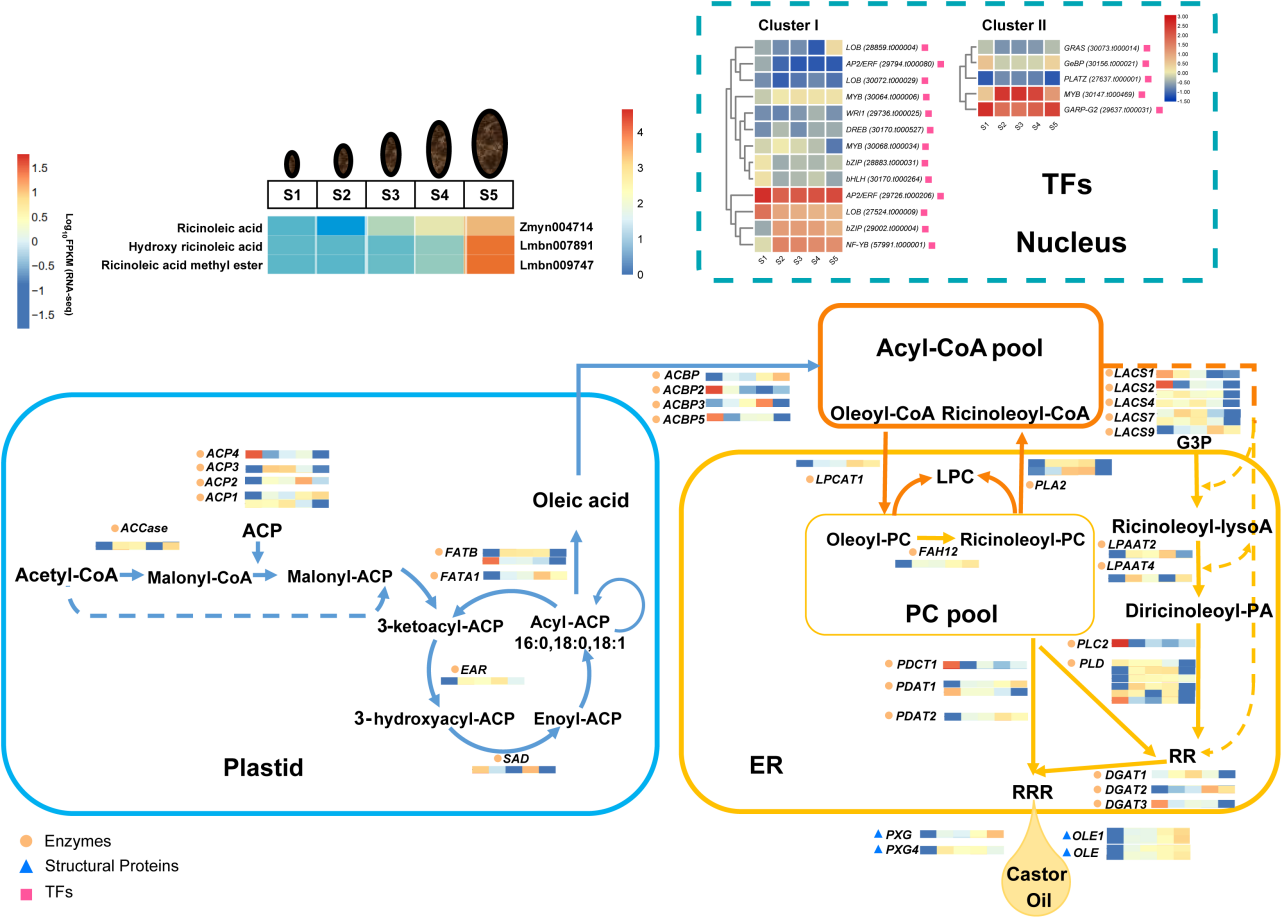
Proposed theoretical model of 66 candidate genes and three strong correlated ricinoleates expression to delineate the dynamic changes during seed development and to model key processes central to castor oil accumulation, including ricinoleic acid biosynthesis, the storage function of oil body-associated proteins, and the regulatory candidates suggested by co-expression networks. The expressions (represented by Log_10_FPKM) of the 42 key enzyme-encoding genes, six oil body related-genes, 18 key transcription factors and three DAMs (hydroxy ricinoleic acid, ricinoleic acid methyl ester and ricinoleic acid) at different seed development stages (S1-S5) in *R. communis* are highlighted in color scales (blue to red). ACBP, acyl CoA-binding protein; ACCase, acetyl-CoA Carboxylase; ACP, acyl carrier protein; DGAT, diacylglycerol acyltransferase; EAR, enoyl-ACP reductase; ER, endoplasmic reticulum; FATA, acyl-ACP thioesterase FatA; FATB, acyl-ACP thioesterase FatB; FAH12, oleate-12-hydroxylase; G3P, sn-glycerol-3-phosphate; LACS, long-chain acyl-CoA synthase; LPAAT, acyl-CoA:lysophosphatidic acid acyltransferase; LPC, lysophosphatidylcholine; LPCAT, lysophosphatidylcholine acyltransferase; OLE, oleosin; PC, phosphatidylcholine; PDAT, phospholipid:diacylglycerol acyltransferase; PDCT, phosphatidylcholine:diacylglycerol choline phosphotransferase; PLA2, phospholipase A2; PLC, phospholipase C; PLD, phospholipase D; PXG, peroxygenase; RR, 1,2- diricinoleoyl-sn-glycerol; RRR, triricinolein; SAD, stearoyl-ACP desaturase. This model was developed based on the transcriptome data of this study and information from Bafor et al. (1991), Cagliari et al. (2010), Chandrasekaran et al. (2014), Tian et al. (2019); Wang et al. (2019a), and Wu et al. (2021).

Based on integrated metabolomic and transcriptomic profiling, along with previous evidence, we constructed a putative co-expression network involving 42 enzyme genes, six oil body-related genes, 18 key TFs, and three strongly correlated ricinoleate metabolites (hydroxy ricinoleic acid, ricinoleic acid methyl ester and ricinoleic acid) to delineate the dynamic changes during seed development and to model key processes central to castor oil accumulation, including ricinoleic acid biosynthesis, the storage function of oil body-associated proteins, and the regulatory candidates suggested by co-expression networks (**Figure 7**). This study systematically identifies four oil body-related candidate genes (*OLEs* and *PXGs*) as potential contributors to castor oil accumulation, and further reveals the key association of the oleate 12-hydroxylase gene *FAH12* with the synthesis and deposition of ricinoleic acid. Furthermore, co-expression analysis of lipid-related genes and transcription factors in clusters I and II identified 11 candidate transcription factors whose expression patterns correlate with lipid synthesis stages. By integrating transcriptome and metabolomic data, we have comprehensively summarized the relevant metabolic pathways involved, providing deeper molecular insights into castor oil accumulation. These findings offer a theoretical foundation for metabolic engineering and future industrial applications of castor and other ricinoleic acid-producing plants.

However, the inferences drawn from our multi-omics profiling and correlation-based analysis, though insightful, constitute a foundational framework rather than definitive mechanistic insight. The connections posited here, including the central association of *FAH12*, *OLEs* and *PXGs*, the co-expression networks involving specific TFs, and the alternative splicing events, are compelling candidate relationships that require direct experimental testing. Consequently, the primary contribution of this work is to identify a series of candidate genes and to provide specific targets and a prioritized list for subsequent functional validation. Future work employing functional validation, such as genetic knockout/complementation, heterologous expression, or enzyme activity assays, will be essential to confirm their specific biochemical functions and regulatory relationships within the castor oil network. Meanwhile, metabolite-gene correlations that these associations are based on putatively annotated metabolites and that future validation with standards is warranted.

## Data Availability

The raw RNA-seq data supporting the conclusions of this article are available in the Genome Sequence Archive (Genomics, Proteomics & Bioinformatics 2025) in National Genomics Data Center (Nucleic Acids Res 2025), China National Center for Bioinformation/Beijing Institute of Genomics, Chinese Academy of Sciences (GSA: CRA037191). The metabolite profile data reported in this article have been deposited in the OMIX, China National Center for Bioinformation/Beijing Institute of Genomics, Chinese Academy of Sciences: Accession No. OMIX014522.
