# Supplementary material for: Integrated metabolome and transcriptome analysis of castor oil accumulation during seed development in Ricinus communis
Source: Front Plant Sci. 2026 Feb 27;17:1763593. doi: 10.3389/fpls.2026.1763593 (PMC12982342; doi:10.3389/fpls.2026.1763593)
Supplement: Supplementary file 1 [file DataSheet1.pdf]

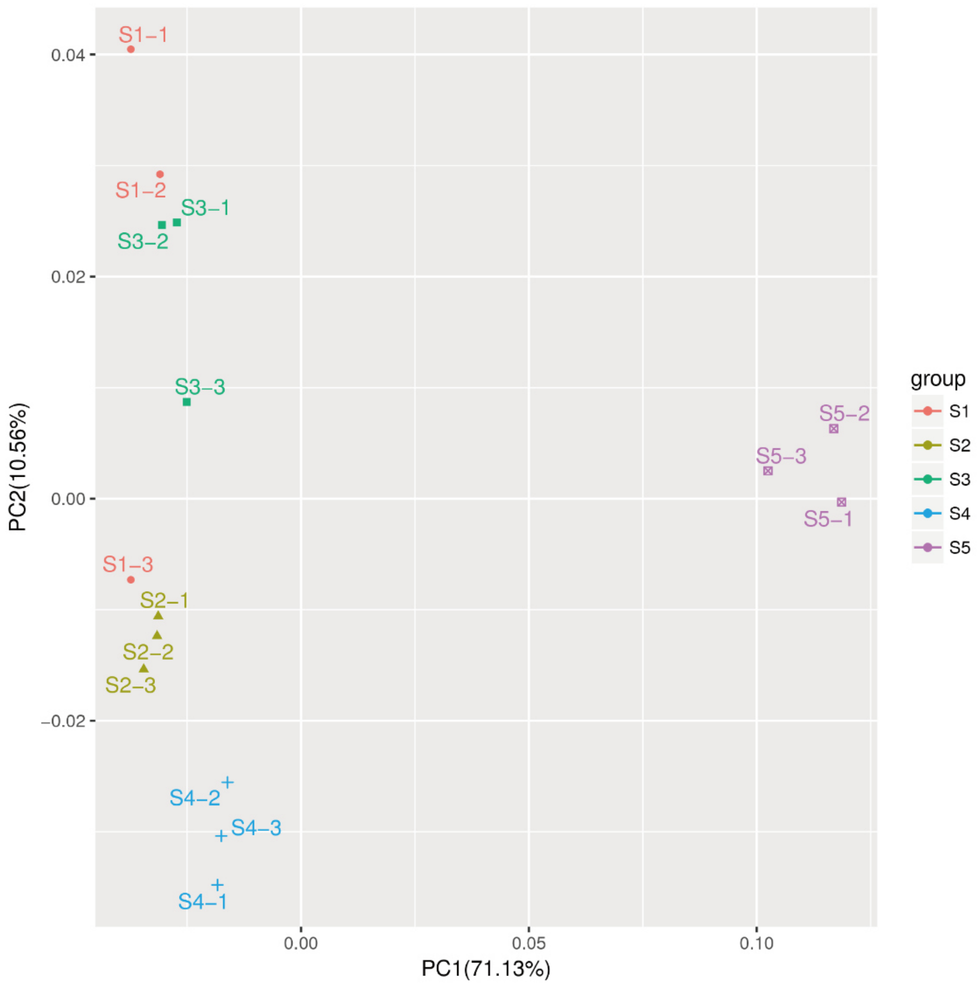

Supplementary Figure 1. PCA analysis of metabolites among five different seed developing stages samples (S1-S5); the x-axis represents the first principal component and the y-axis represents the second principal.

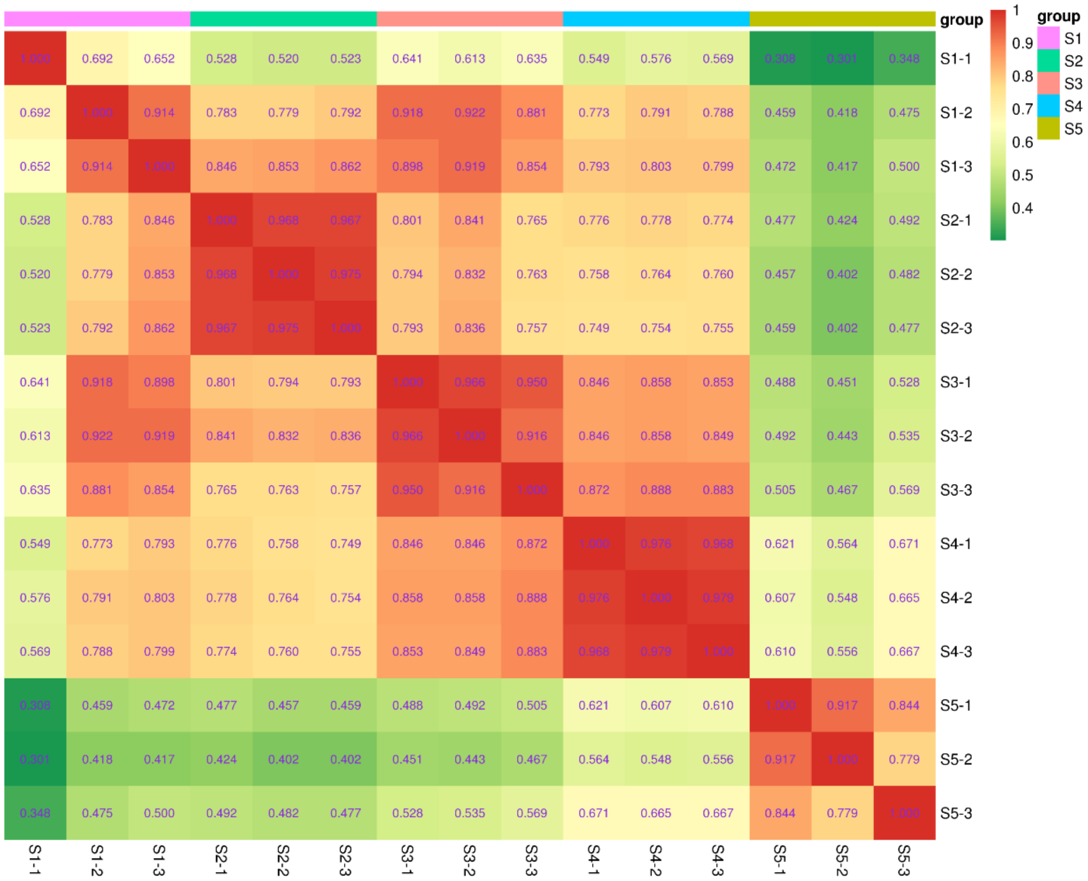

Supplementary Figure 2. Pearson's correlation coefficients among *R. communis* five different samples (S1, S2, S3, S4 and S5) based on global expression profiles of metabolome data.

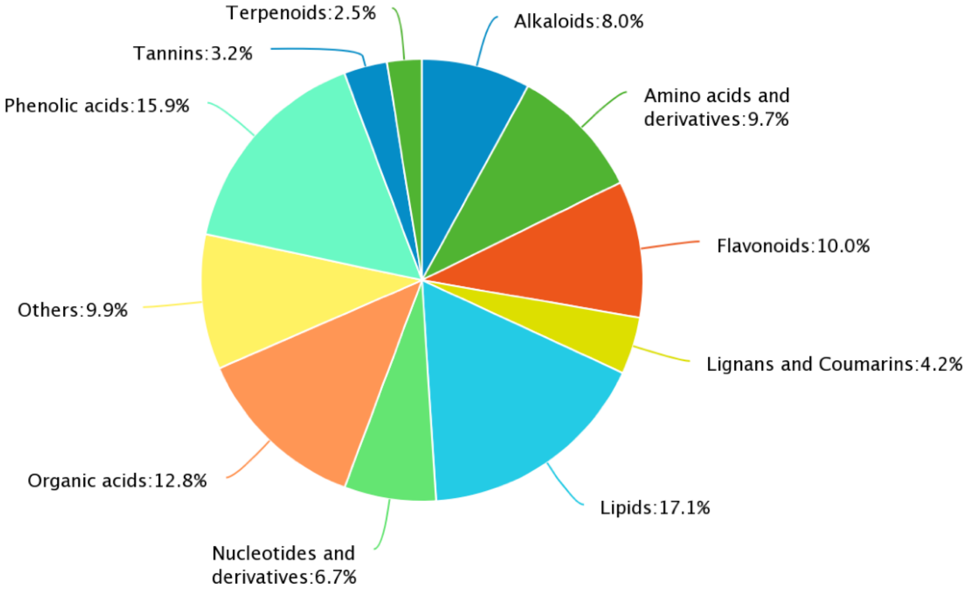

Supplementary Figure 3. Component analysis of the identified 790 metabolites. Eleven main metabolites are shown beside the chart.

(A)

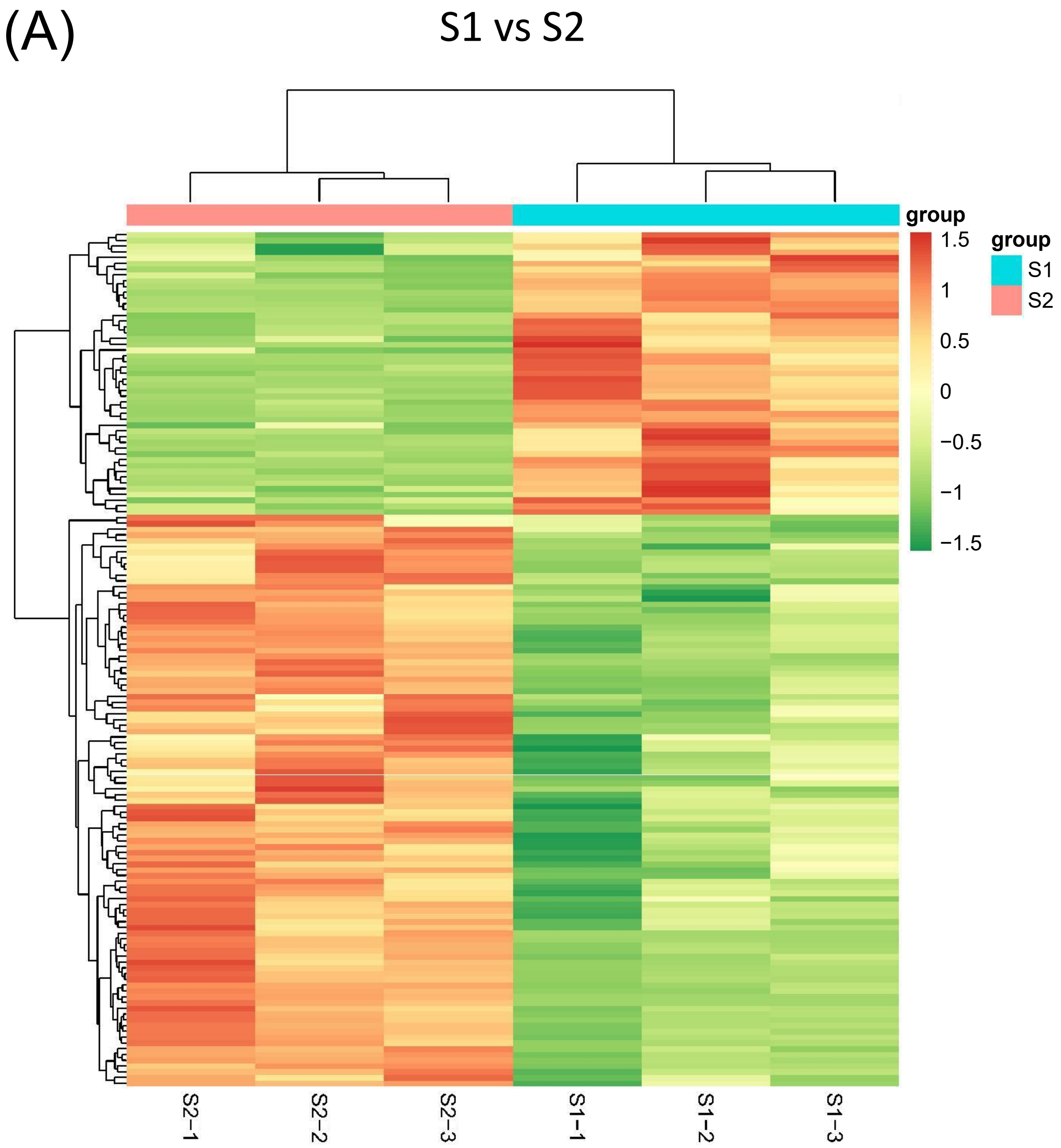

(B)

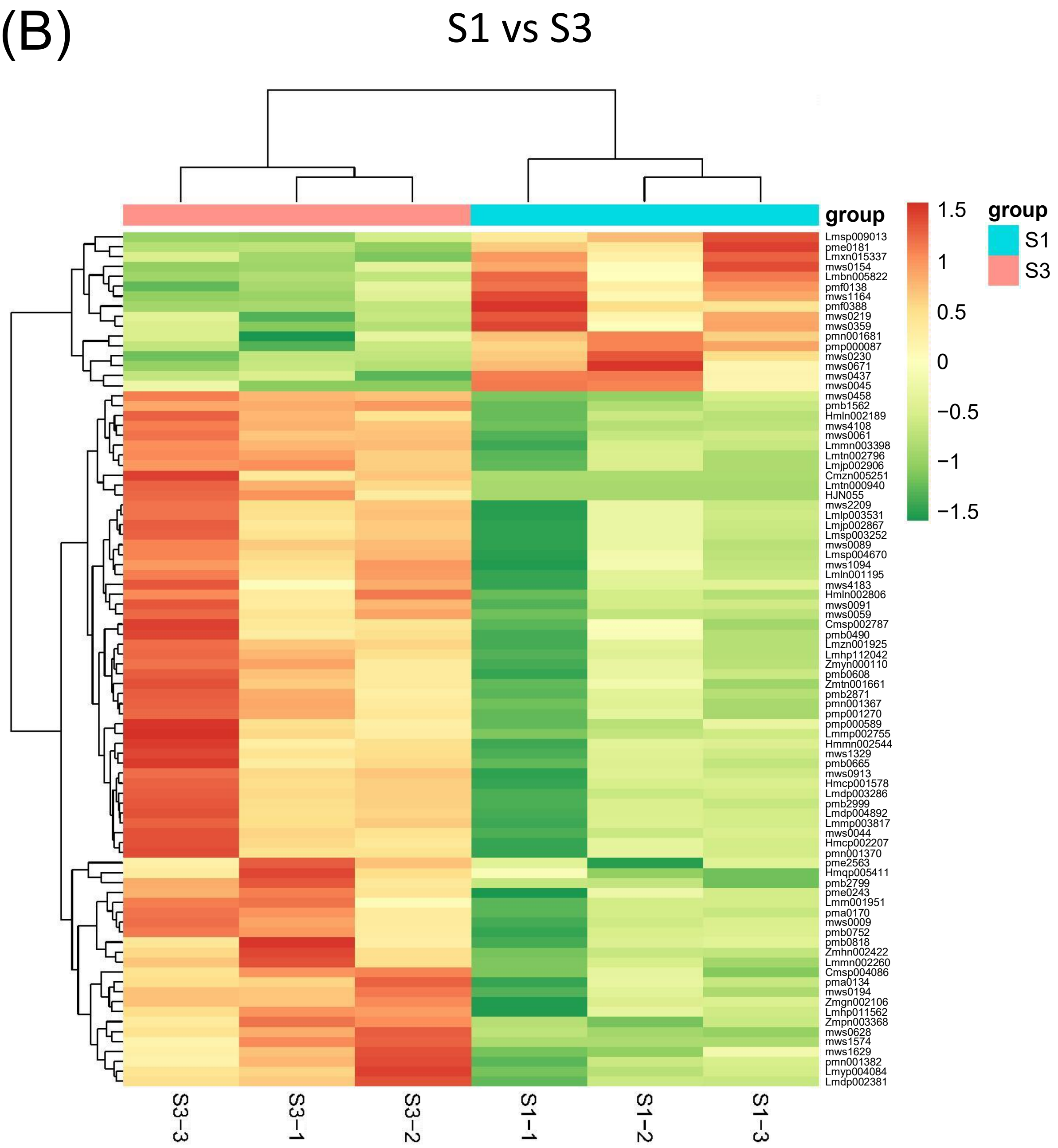

S1 vs S3
group
Lmsp009013
pme0181
Lmxn015337
mws0154
Lmbn005822
pmf0138
mws1164
pmf0388
mws0219
mws0359
pmn001681
pmp000087
mws0230
mws0671
mws0437
mws0045
mws0458
pmb1562
Hmln002189
mws4108
mws0061
Lmmn003398
Lmtn002796
Lmjp002906
Cmzn005251
Lmtn000940
HJN055
mws2209
Lmlp003531
Lmjp002867
Lmsp003252
mws0089
Lmsp004670
mws1094
Lmln001195
mws4183
Hmln002806
mws0091
mws0059
Cmsp002787
pmb0490
Lmzn001925
Lmhp112042
Zmyn000110
pmb0608
Zmtn001661
pmb2871
pmn001367
pmp001270
pmp000589
Lmmp002755
Hmmn002544
mws1329
pmb0665
mws0913
Hmcp001578
Lmdp003286
pmb2999
Lmdp004892
Lmmp003817
mws0044
Hmcp002207
pmn001370
pme2563
Hmqp005411
pmb2799
pme0243
Lmrn001951
pma0170
mws0009
pmb0752
pmb0818
Zmhn002422
Lmmn002260
Cmsp004086
pma0134
mws0194
Zmgn002106
Lmhp011562
Zmpn003368
mws0628
mws1574
mws1629
pmn001382
Lmyp004084
Lmdp002381
S3−3
S3−1
S3−2
S1−1
S1−2
S1−3
1.5
1
0.5
0
−0.5
−1
−1.5
group
S1
S3

(C)

S1 vs S4

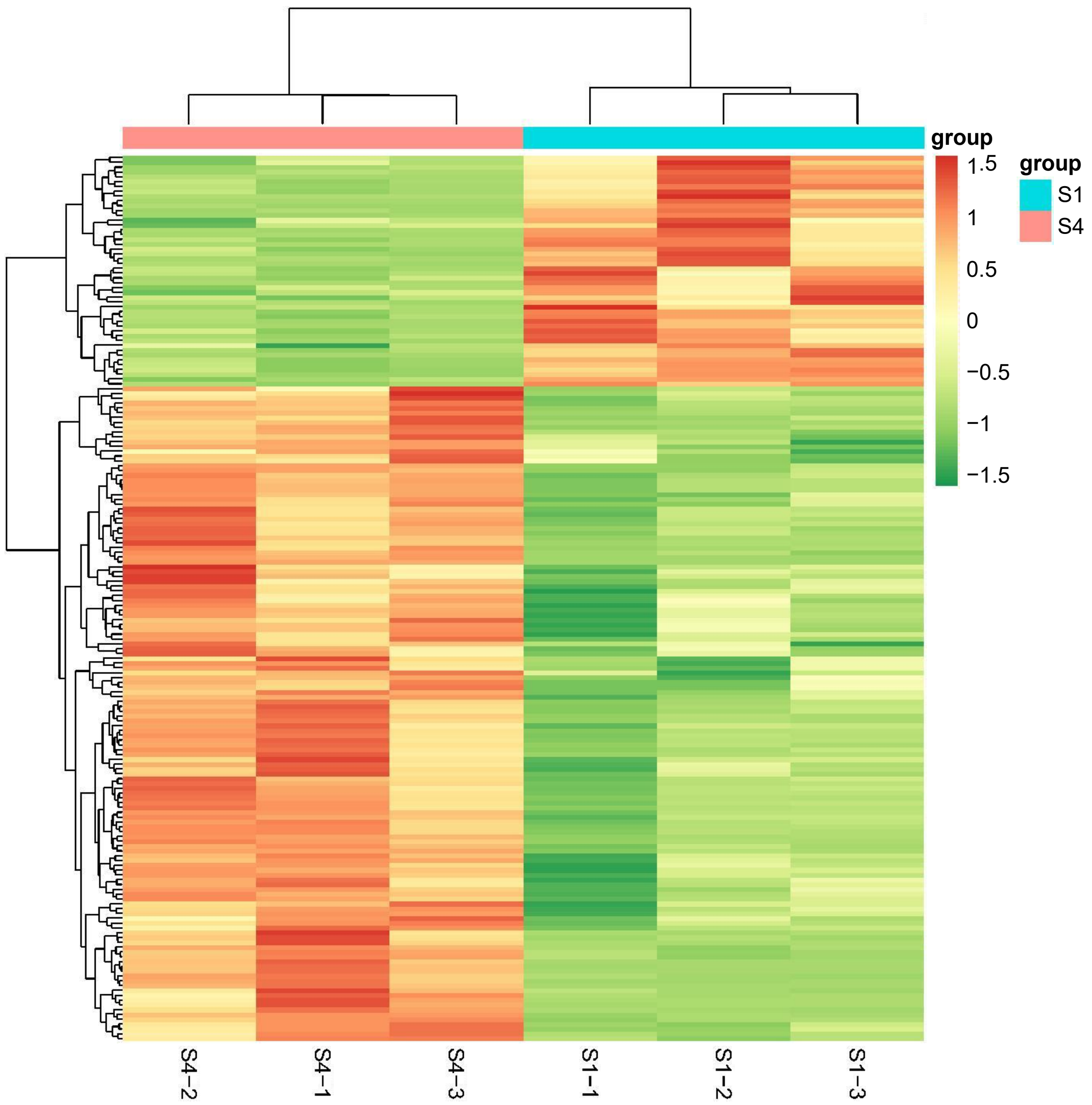

(D)

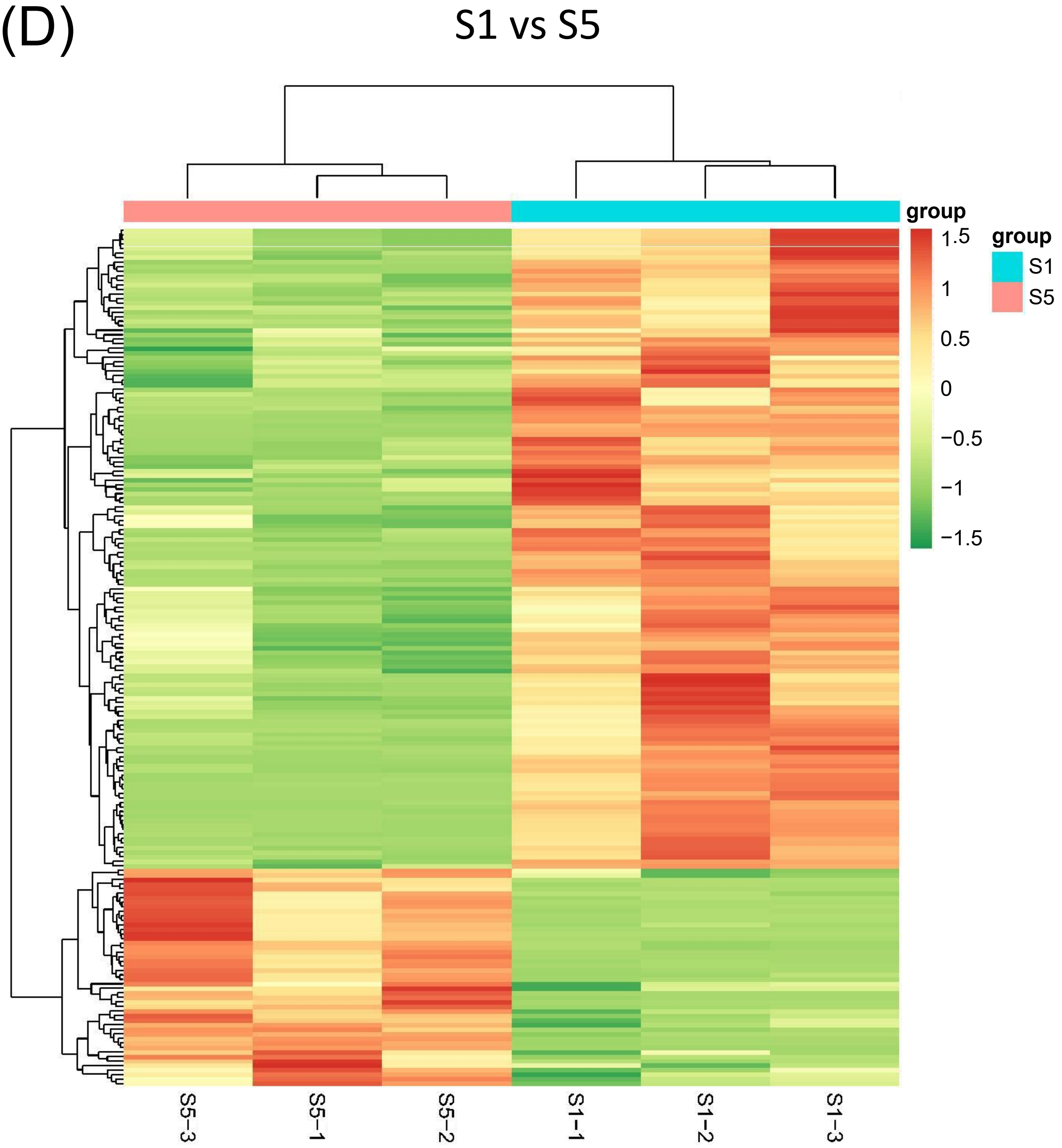

(E)

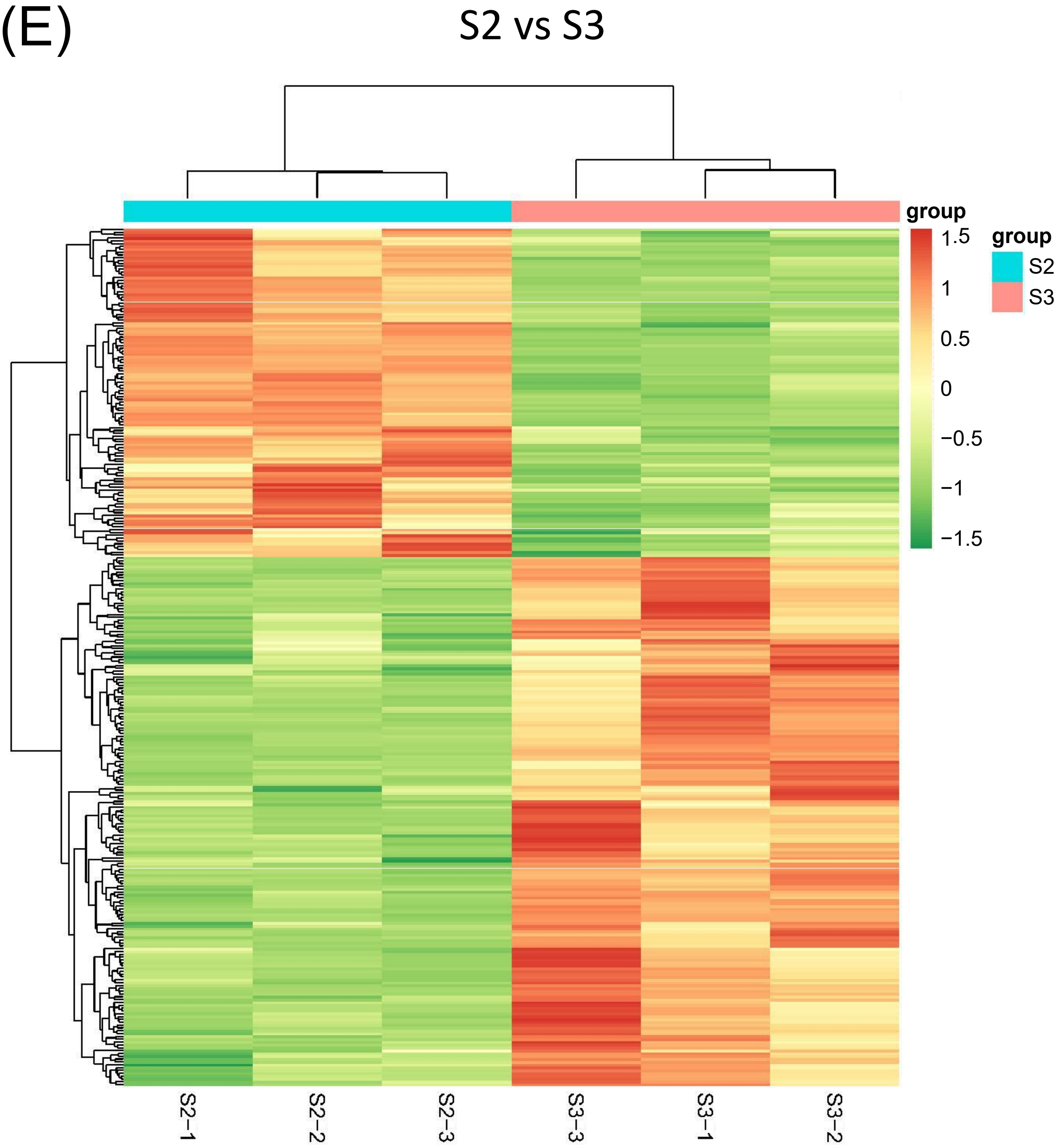

(F)

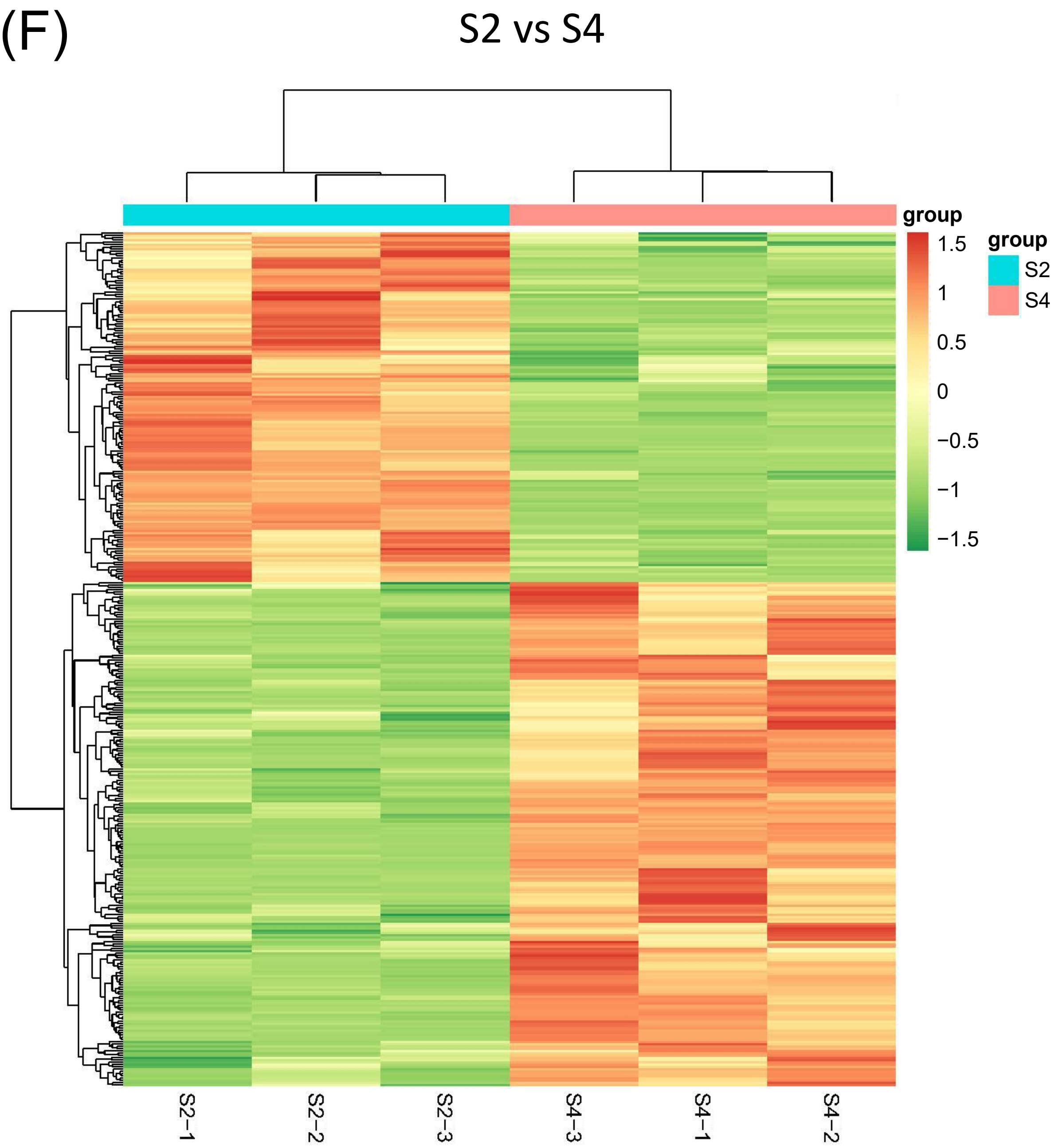

(G)

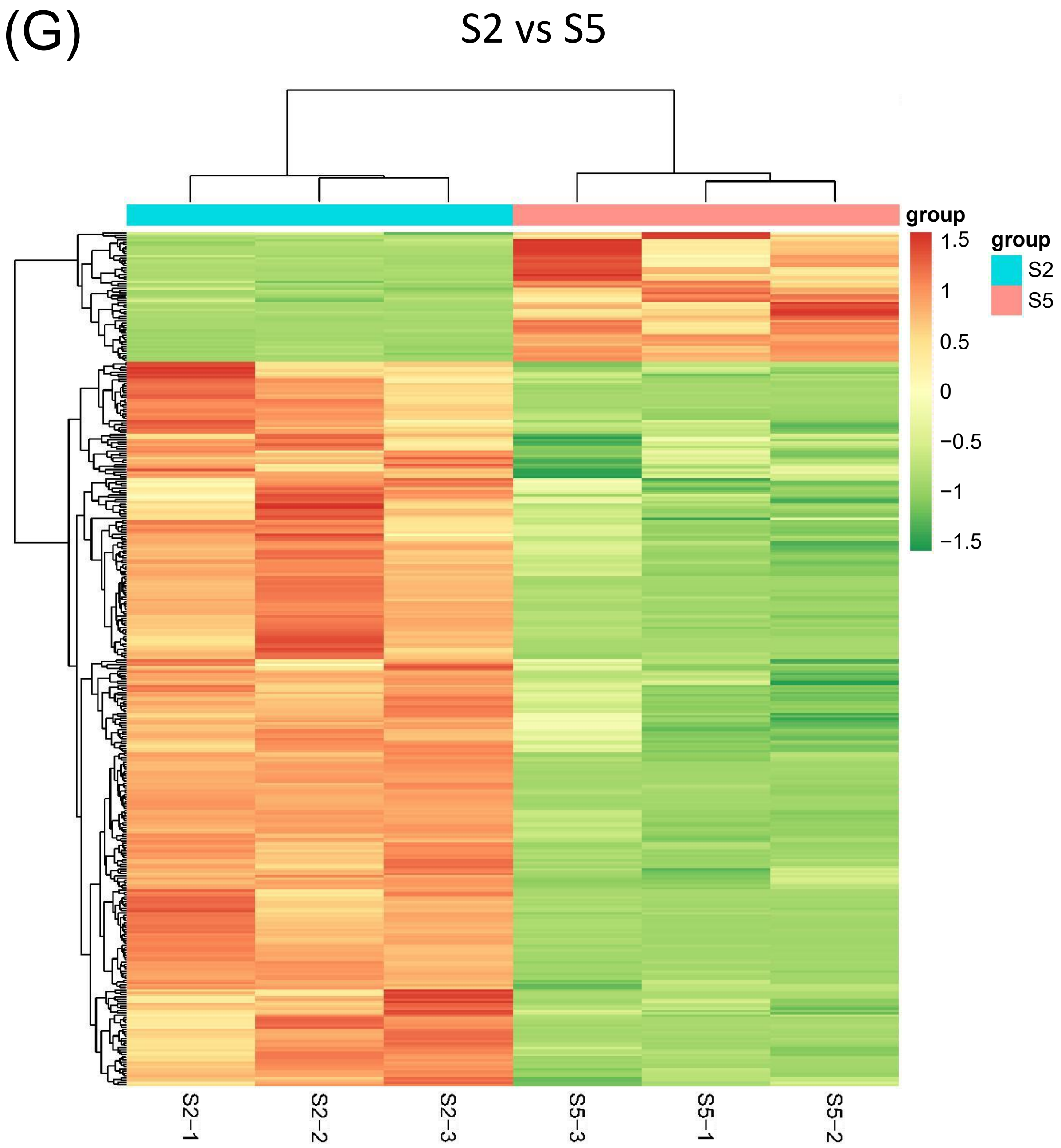

(H)

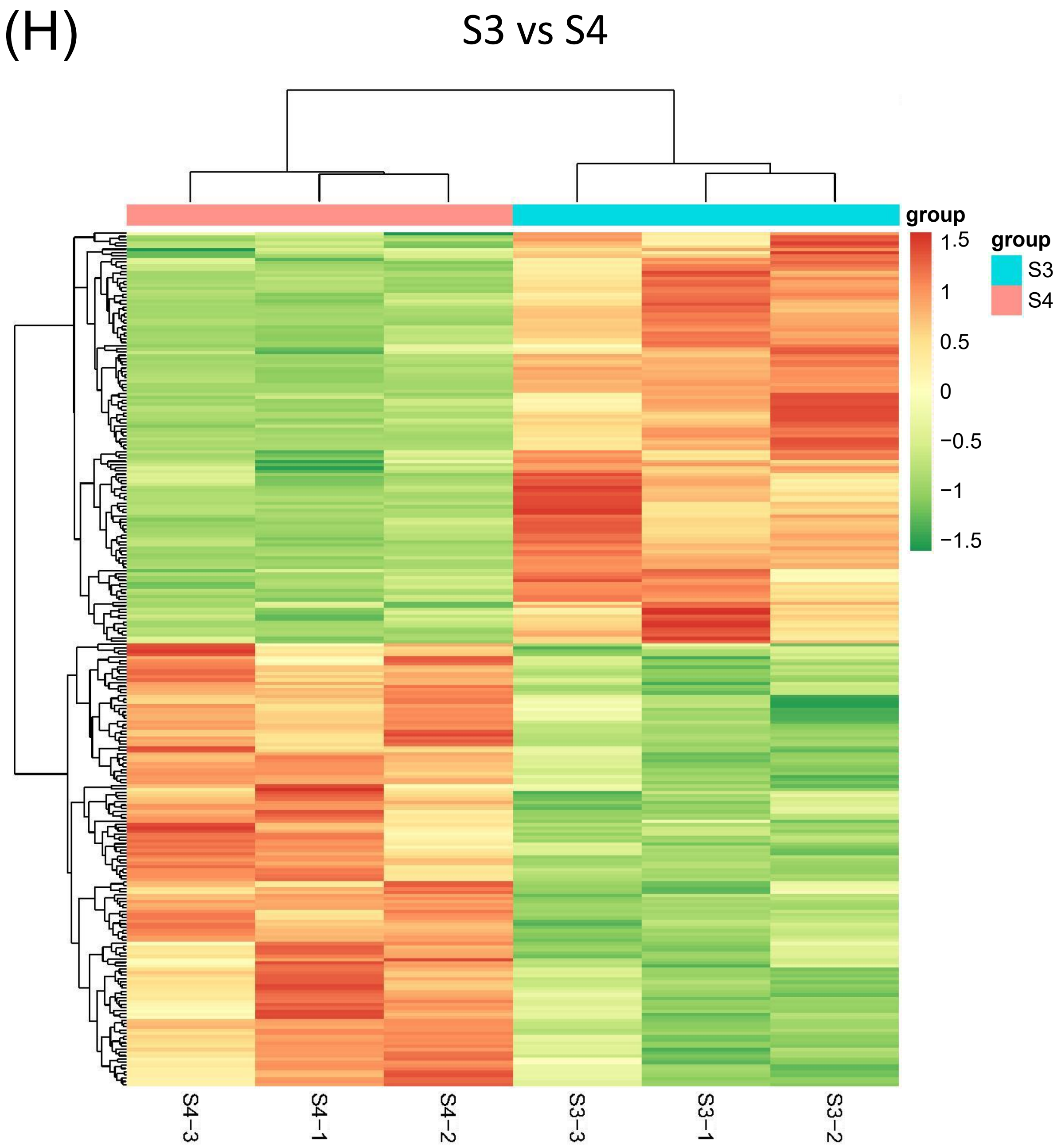

(I)

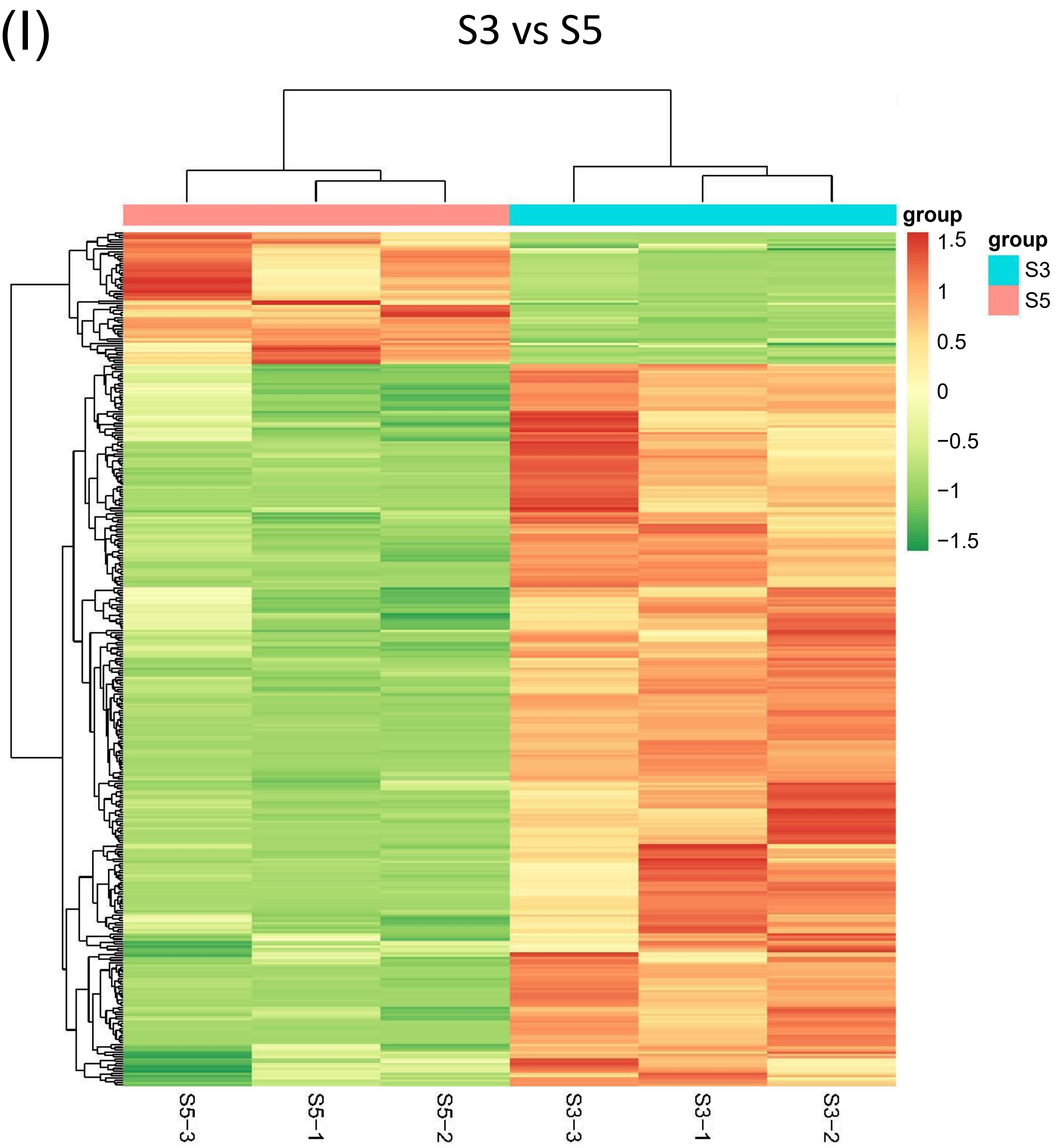

(J)

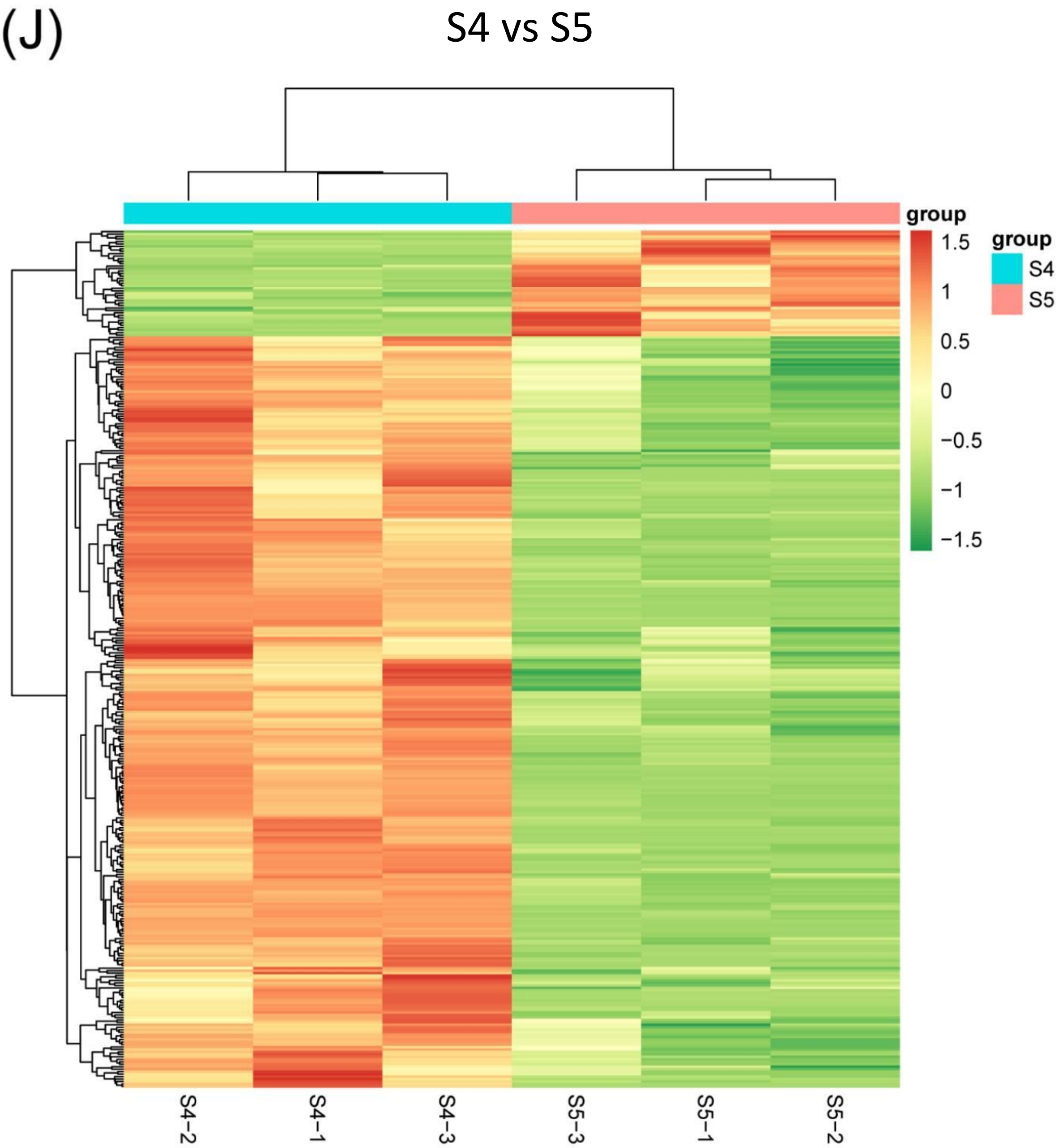

Supplementary Figure 4. Heat map representing the hierarchical cluster analysis among different group comparisons. (A) S1 vs S2, (B) S1 vs S3, (C) S1 vs S4, (D) S1 vs S5, (E) S2 vs S3, (F) S2 vs S4, (G) S2 vs S5, (H) S3 vs S4, (I) S3 vs S5, and (J) S4 vs S5.

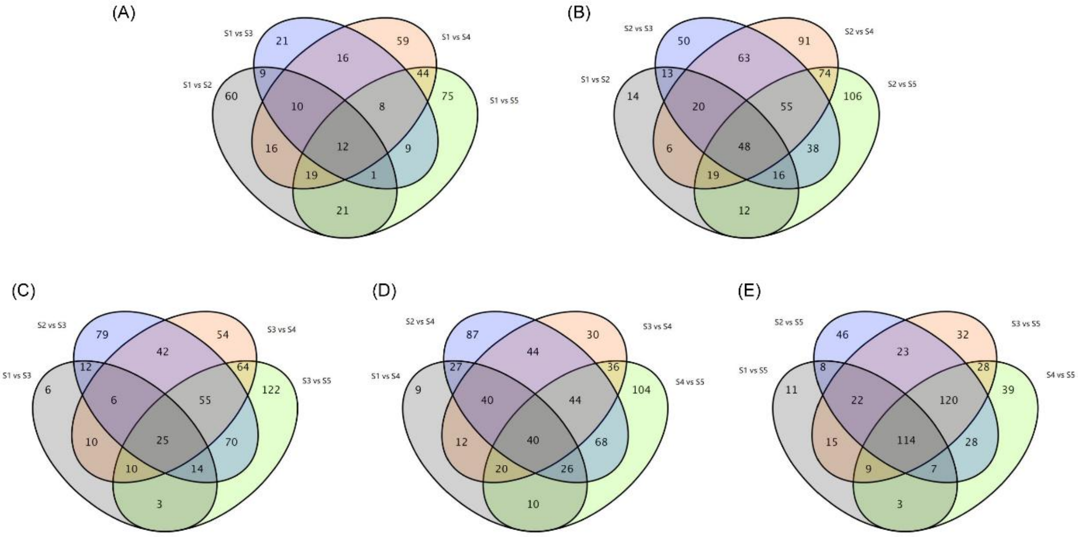

Supplementary Figure 5. Venn diagram depicting the shared and unique DAMs among different group comparisons. (A) S1 vs S2, S1 vs S3, S1 vs S4 and S1 vs S5, (B**)** S1 vs S2, S2 vs S3, S2 vs S4 and S2 vs S5, (C) S1 vs S3, S2 vs S3, S3 vs S4 and S3 vs S5, (D) S1 vs S4, S2 vs S4, S3 vs S4 and S4 vs S5, and (E) S1 vs S5, S2 vs S5, S3 vs S5 and S4 vs S5.

(A)

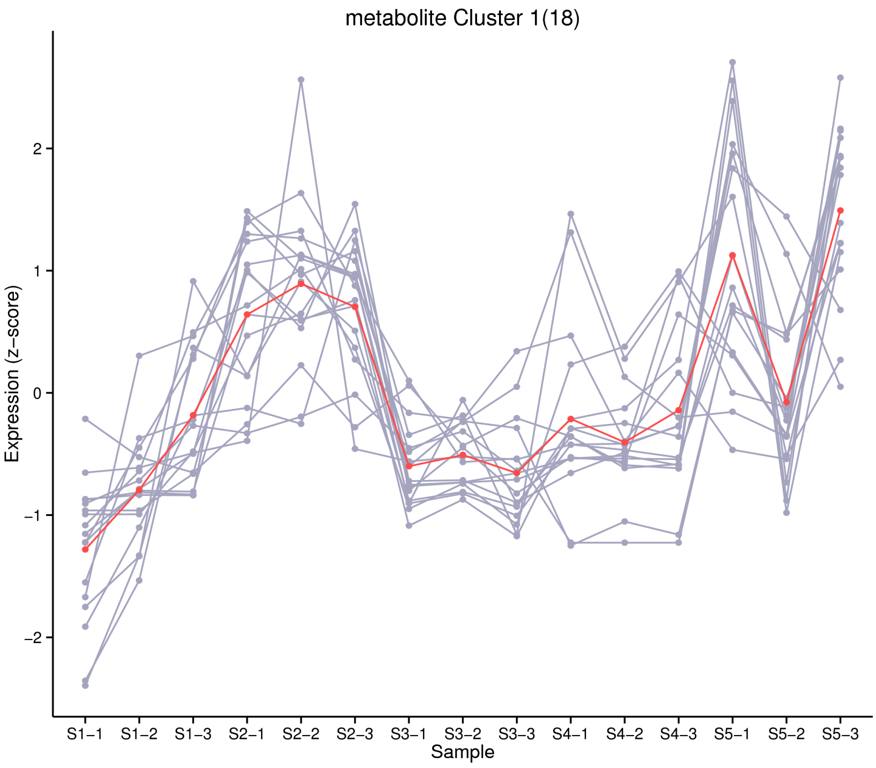

metabolite Cluster 1(18)
Expression (z−score)
2
1
0
−1
−2
S1−1
S1−2
S1−3
S2−1
S2−2
S2−3
S3−1
S3−2
S3−3
S4−1
S4−2
S4−3
S5−1
S5−2
S5−3
Sample

(B)

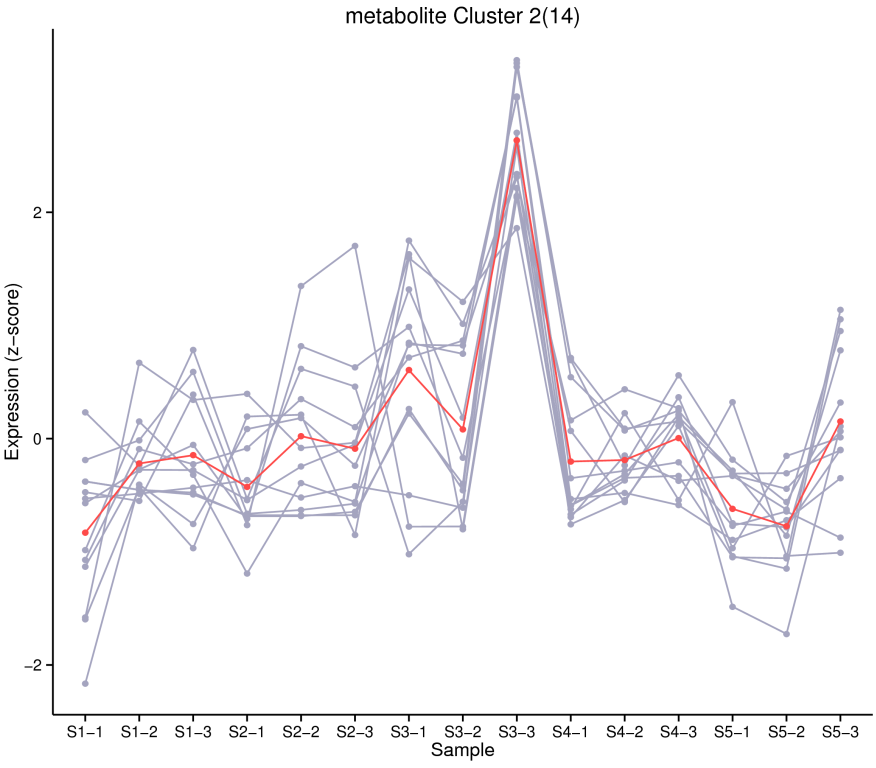

metabolite Cluster 2(14)
Expression (z−score)
2
0
−2
S1−1
S1−2
S1−3
S2−1
S2−2
S2−3
S3−1
S3−2
S3−3
S4−1
S4−2
S4−3
S5−1
S5−2
S5−3
Sample

(C)

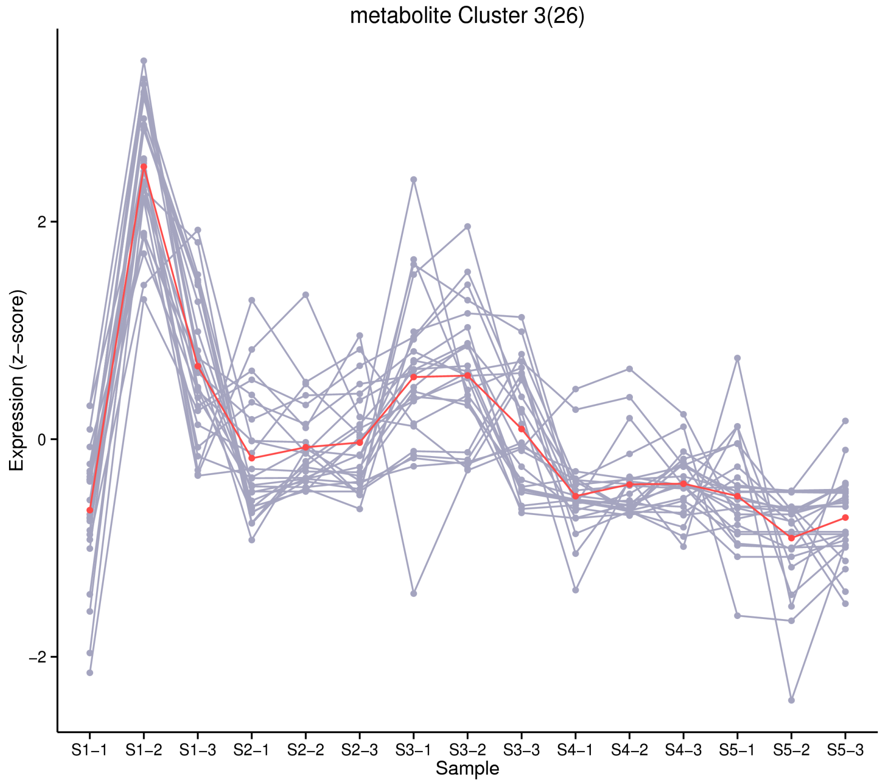
metabolite Cluster 3(26)
Expression (z−score)
2
0
−2
S1−1
S1−2
S1−3
S2−1
S2−2
S2−3
S3−1
S3−2
S3−3
S4−1
S4−2
S4−3
S5−1
S5−2
S5−3
Sample

(D)

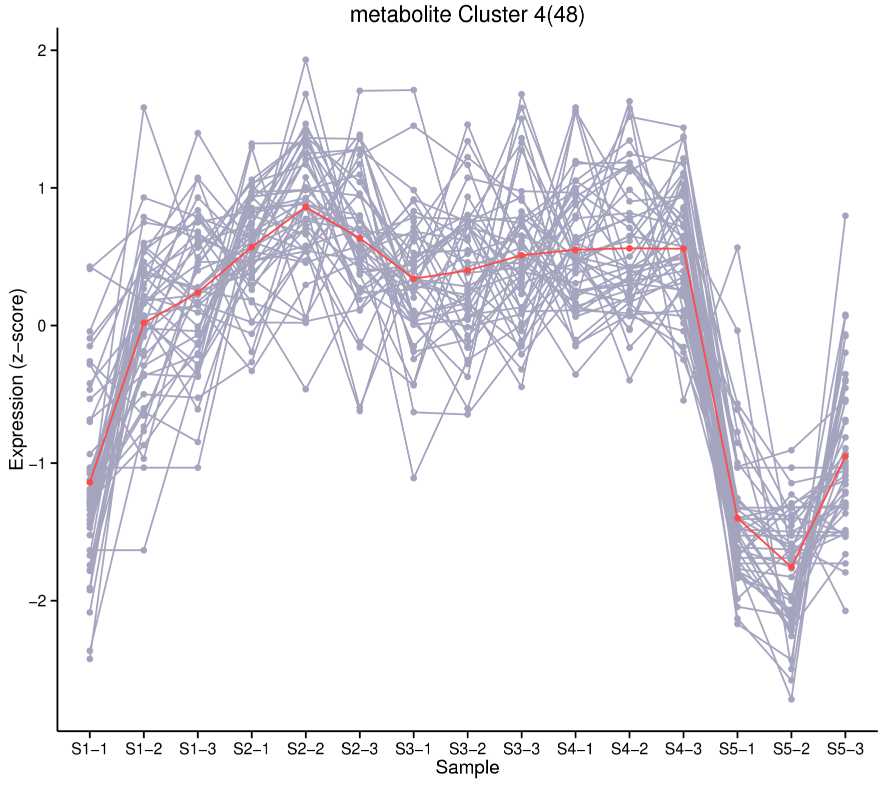
metabolite Cluster 4(48)
Expression (z−score)
2
1
0
−1
−2
S1−1
S1−2
S1−3
S2−1
S2−2
S2−3
S3−1
S3−2
S3−3
S4−1
S4−2
S4−3
S5−1
S5−2
S5−3
Sample

(E)

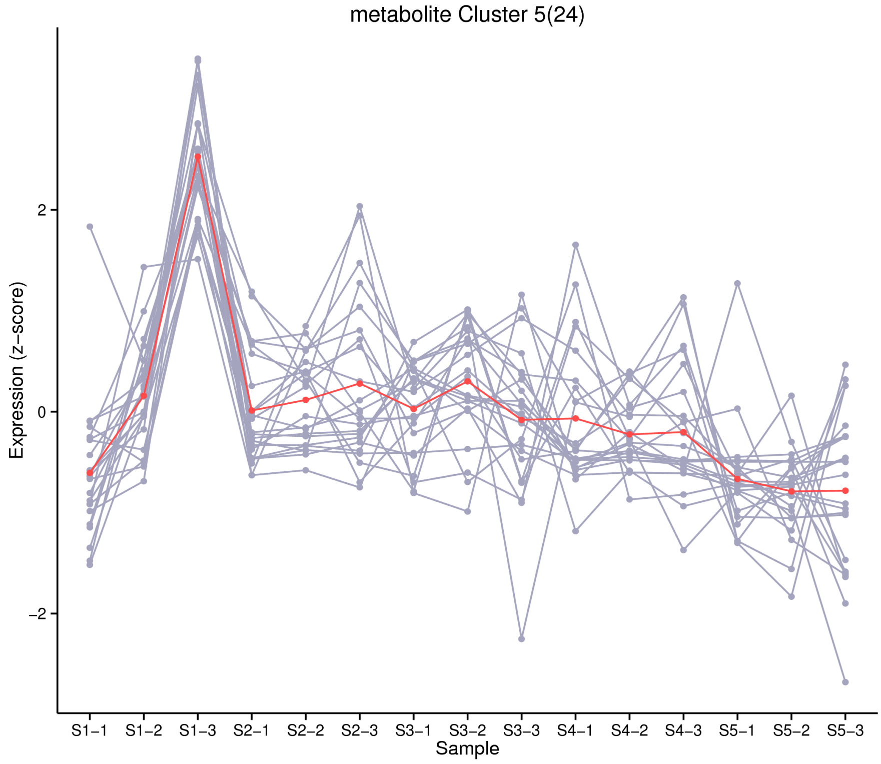
metabolite Cluster 5(24)
Expression (z−score)
2
0
−2
S1−1
S1−2
S1−3
S2−1
S2−2
S2−3
S3−1
S3−2
S3−3
S4−1
S4−2
S4−3
S5−1
S5−2
S5−3
Sample

(F)

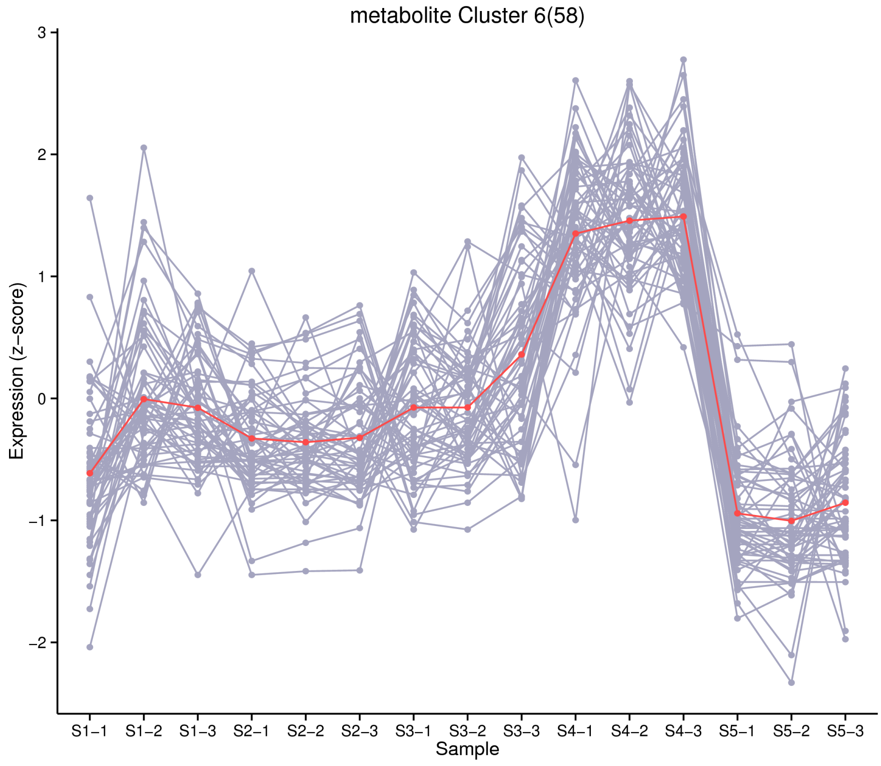
metabolite Cluster 6(58)
Expression (z−score)
3
2
1
0
−1
−2
S1−1
S1−2
S1−3
S2−1
S2−2
S2−3
S3−1
S3−2
S3−3
S4−1
S4−2
S4−3
S5−1
S5−2
S5−3
Sample

(G)

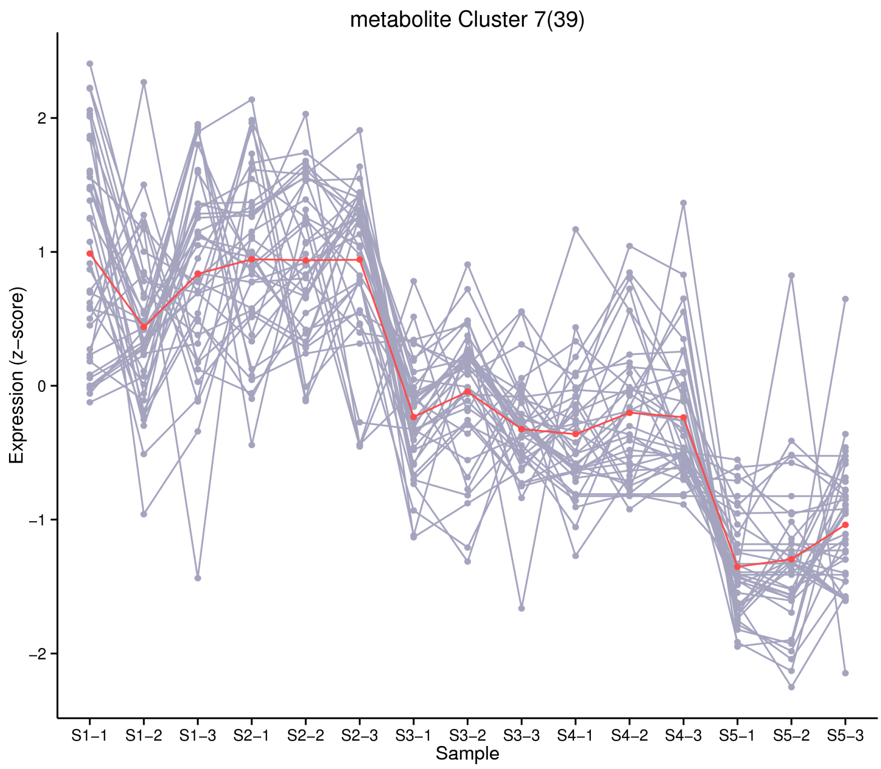
metabolite Cluster 7(39)
Expression (z−score)
2
1
0
−1
−2
S1−1
S1−2
S1−3
S2−1
S2−2
S2−3
S3−1
S3−2
S3−3
S4−1
S4−2
S4−3
S5−1
S5−2
S5−3
Sample

(H)

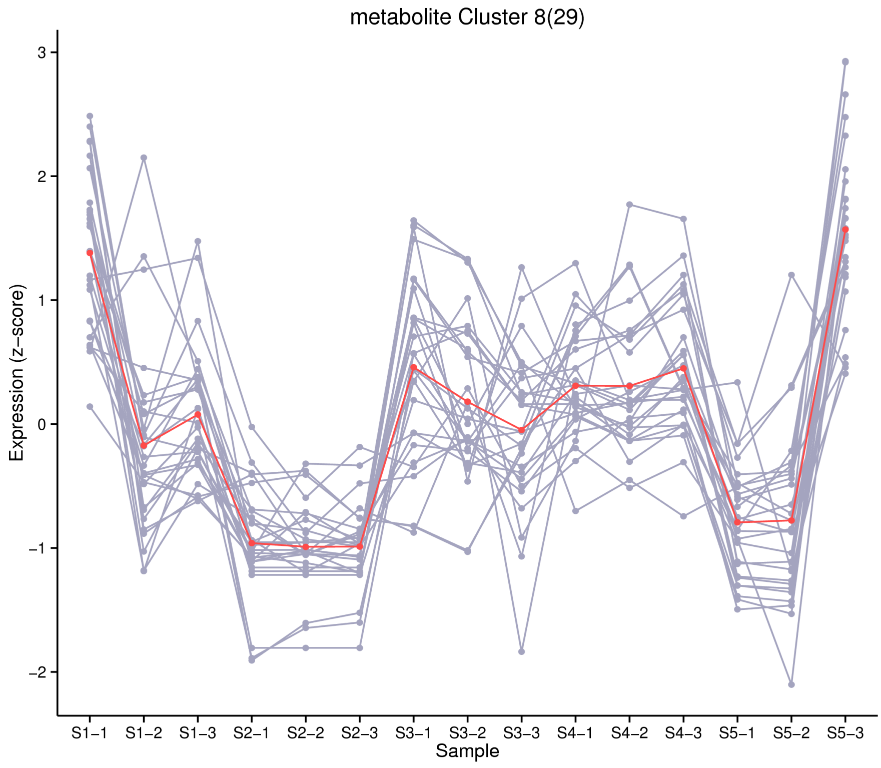
metabolite Cluster 8(29)
Expression (z−score)
3
2
1
0
−1
−2
S1−1
S1−2
S1−3
S2−1
S2−2
S2−3
S3−1
S3−2
S3−3
S4−1
S4−2
S4−3
S5−1
S5−2
S5−3
Sample

(I)

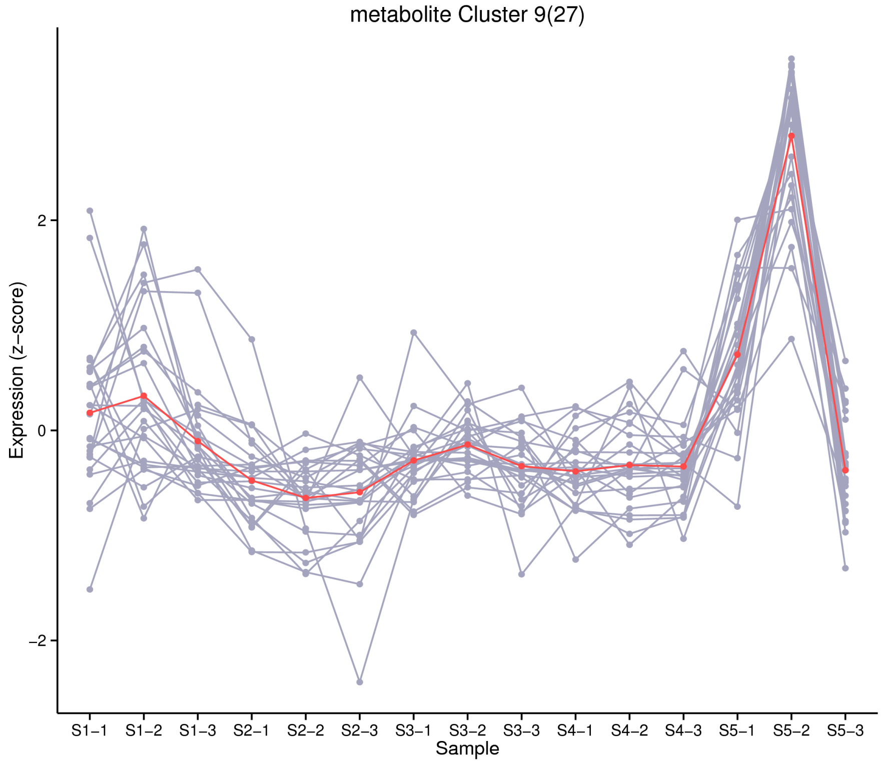
metabolite Cluster 9(27)
Expression (z−score)
2
0
−2
S1−1
S1−2
S1−3
S2−1
S2−2
S2−3
S3−1
S3−2
S3−3
S4−1
S4−2
S4−3
S5−1
S5−2
S5−3
Sample

(J)

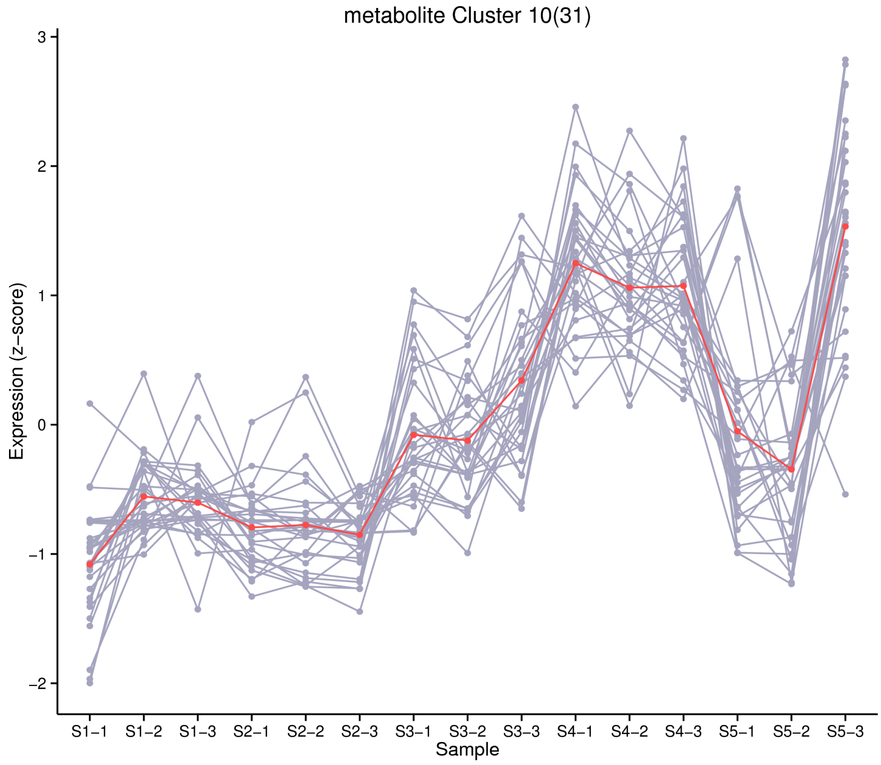
metabolite Cluster 10(31)
Expression (z−score)
3
2
1
0
−1
−2
S1−1
S1−2
S1−3
S2−1
S2−2
S2−3
S3−1
S3−2
S3−3
S4−1
S4−2
S4−3
S5−1
S5−2
S5−3
Sample

(K)

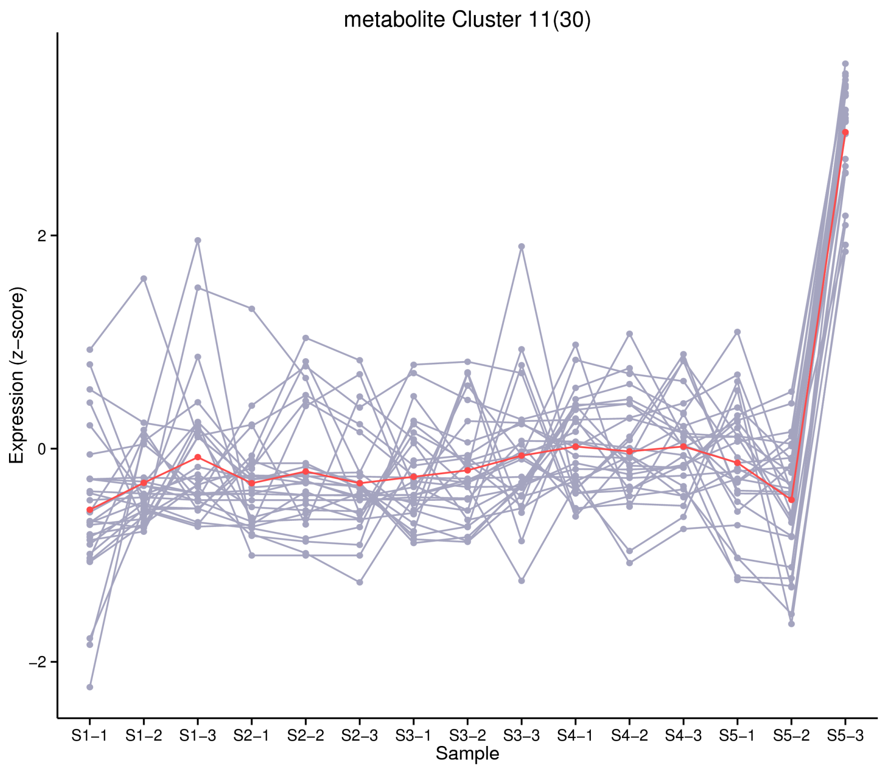

metabolite Cluster 11(30)
Expression (z−score)
2
0
−2
S1−1
S1−2
S1−3
S2−1
S2−2
S2−3
S3−1
S3−2
S3−3
S4−1
S4−2
S4−3
S5−1
S5−2
S5−3
Sample

(L)

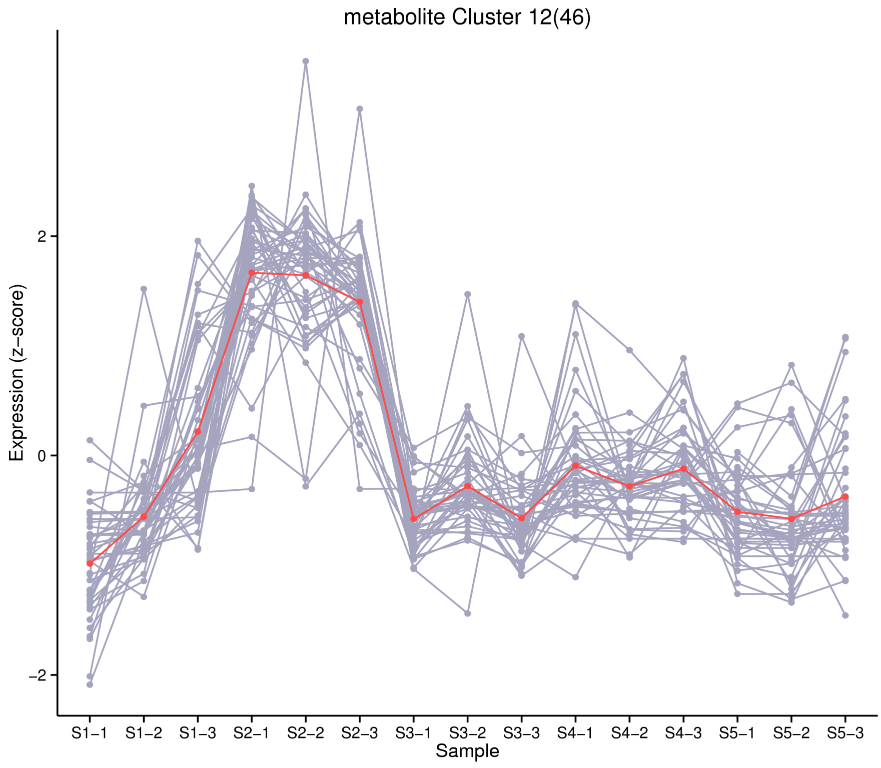

metabolite Cluster 12(46)
Expression (z−score)
2
0
−2
S1−1
S1−2
S1−3
S2−1
S2−2
S2−3
S3−1
S3−2
S3−3
S4−1
S4−2
S4−3
S5−1
S5−2
S5−3
Sample

(M)

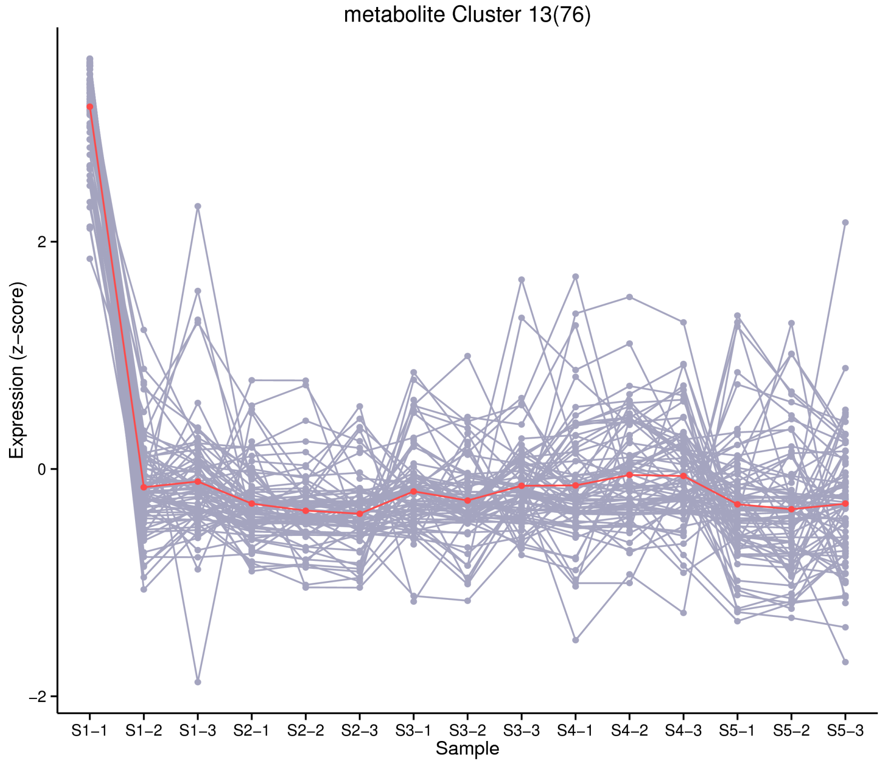
metabolite Cluster 13(76)
Expression (z−score)
2
0
−2
S1−1
S1−2
S1−3
S2−1
S2−2
S2−3
S3−1
S3−2
S3−3
S4−1
S4−2
S4−3
S5−1
S5−2
S5−3
Sample

(N)

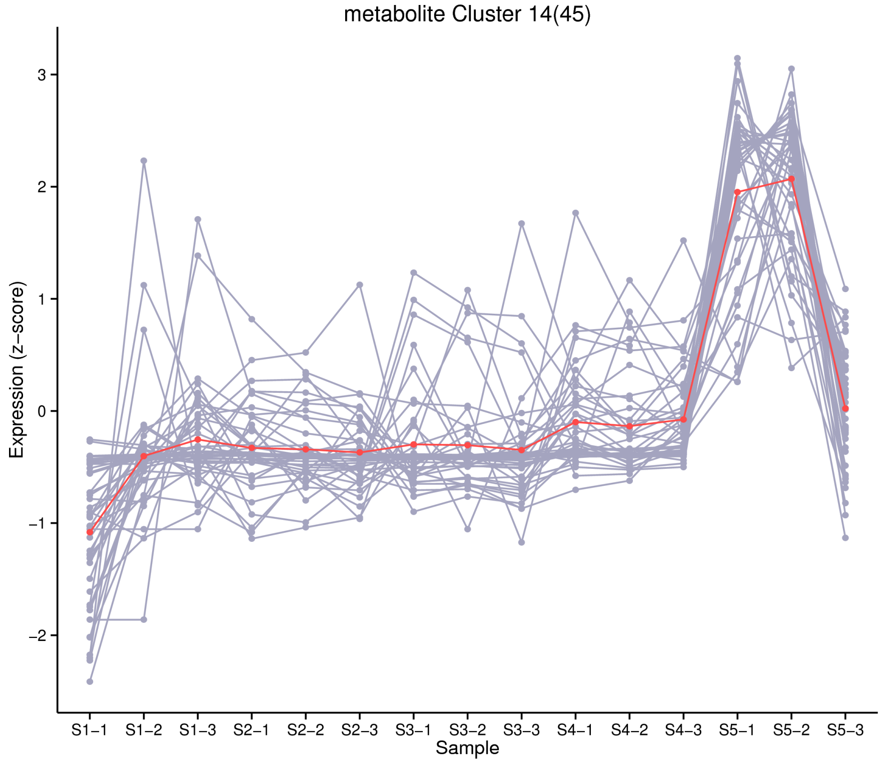
metabolite Cluster 14(45)
Expression (z−score)
3
2
1
0
−1
−2
S1−1
S1−2
S1−3
S2−1
S2−2
S2−3
S3−1
S3−2
S3−3
S4−1
S4−2
S4−3
S5−1
S5−2
S5−3
Sample

(O)

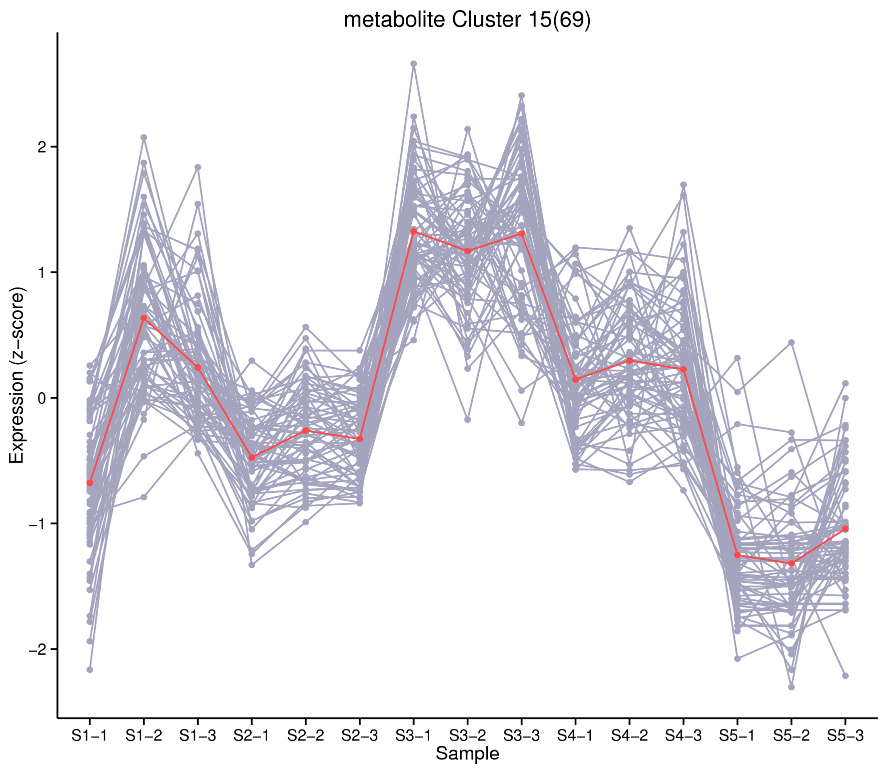

metabolite Cluster 15(69)
Expression (z−score)
2
1
0
−1
−2
S1−1 S1−2 S1−3 S2−1 S2−2 S2−3 S3−1 S3−2 S3−3 S4−1 S4−2 S4−3 S5−1 S5−2 S5−3
Sample

(P)

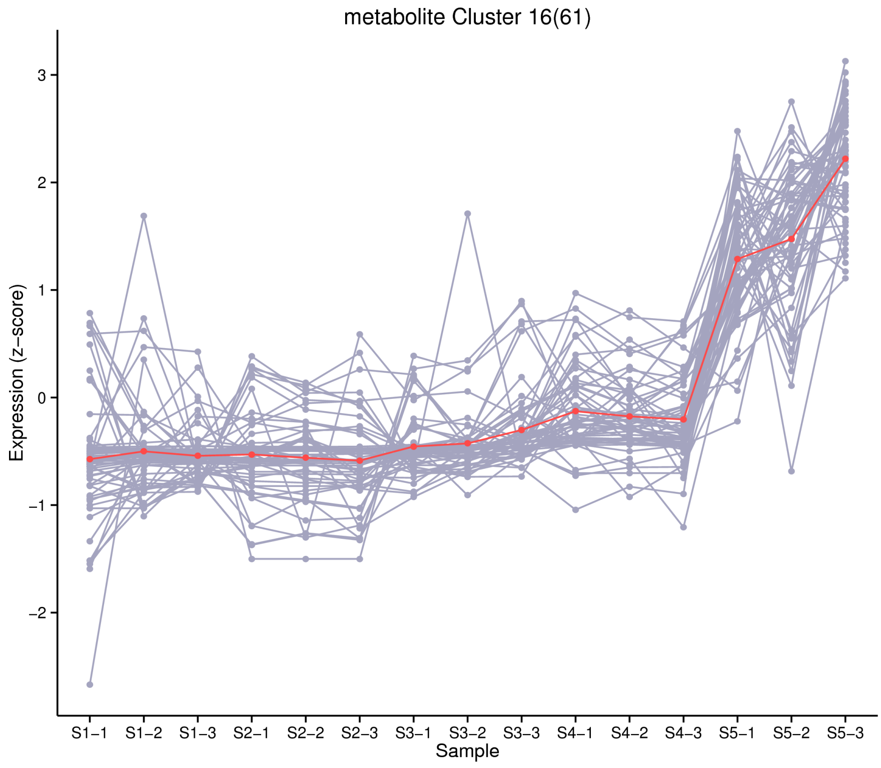

metabolite Cluster 16(61)
Expression (z−score)
3
2
1
0
−1
−2
S1−1 S1−2 S1−3 S2−1 S2−2 S2−3 S3−1 S3−2 S3−3 S4−1 S4−2 S4−3 S5−1 S5−2 S5−3
Sample

(Q)

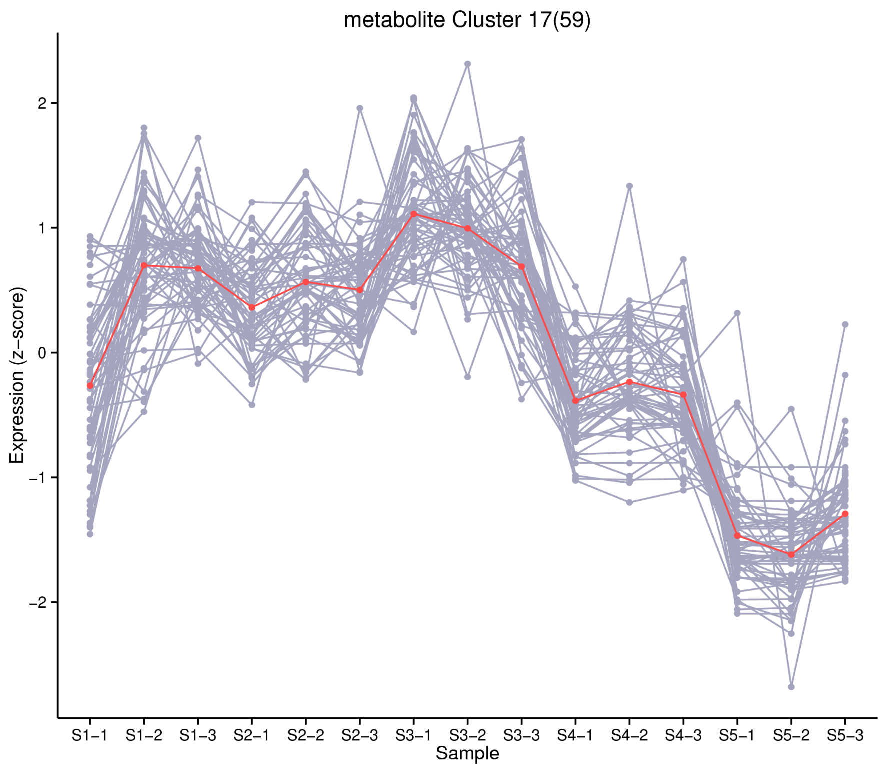
metabolite Cluster 17(59)
Expression (z−score)
2
1
0
−1
−2
S1−1
S1−2
S1−3
S2−1
S2−2
S2−3
S3−1
S3−2
S3−3
S4−1
S4−2
S4−3
S5−1
S5−2
S5−3
Sample

(R)

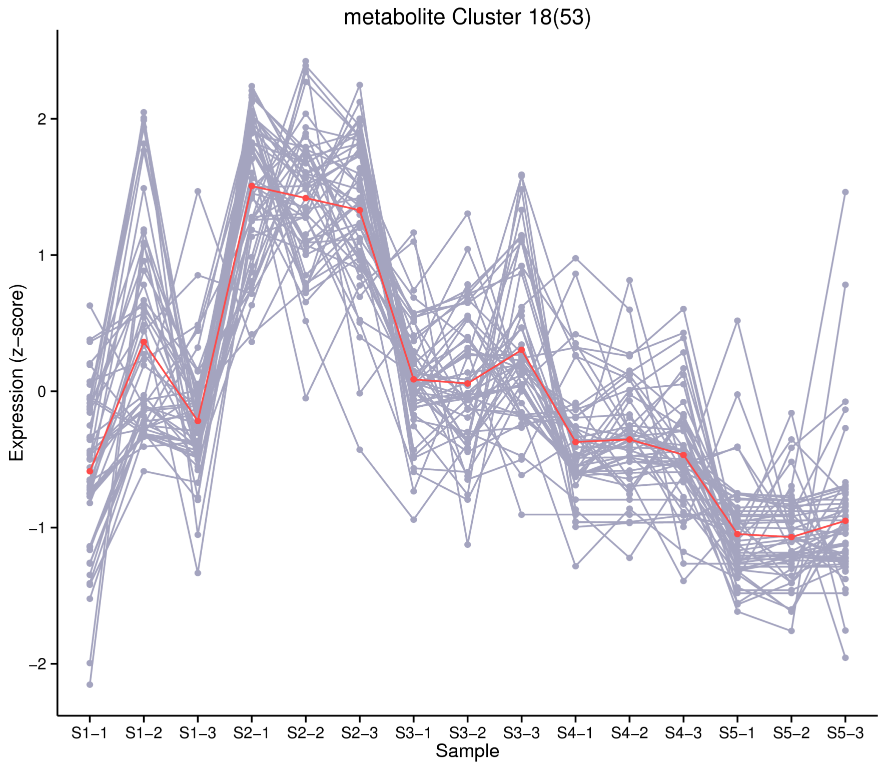
metabolite Cluster 18(53)
Expression (z−score)
2
1
0
−1
−2
S1−1
S1−2
S1−3
S2−1
S2−2
S2−3
S3−1
S3−2
S3−3
S4−1
S4−2
S4−3
S5−1
S5−2
S5−3
Sample

(S)

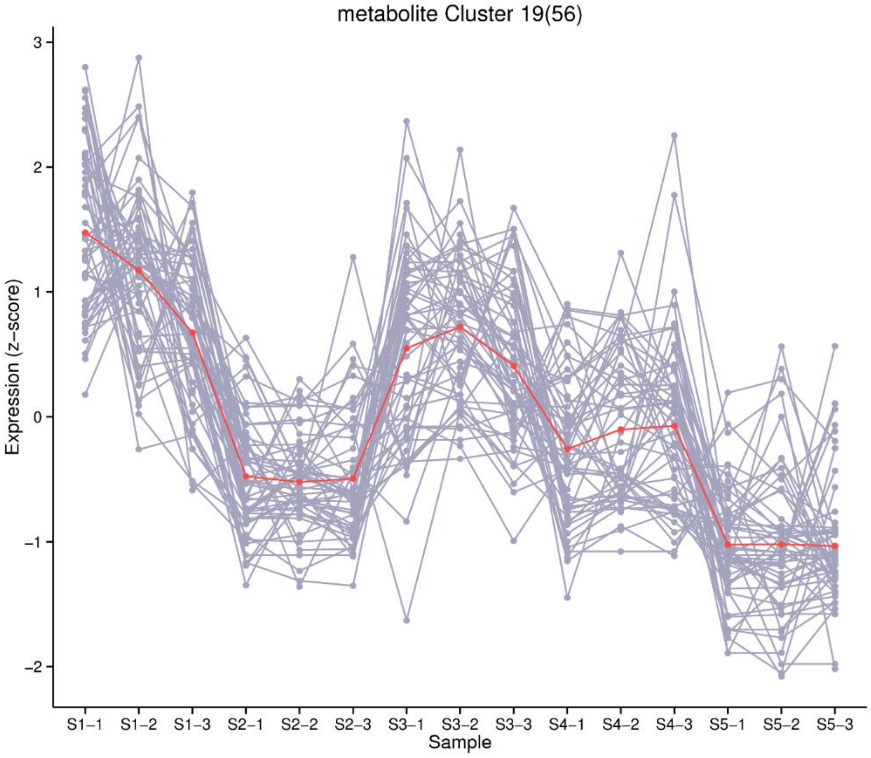

Supplementary Figure 6. All DAMs were classified into 19 clusters (A-S) according to the expression trend of five time points during seed development.

(A)

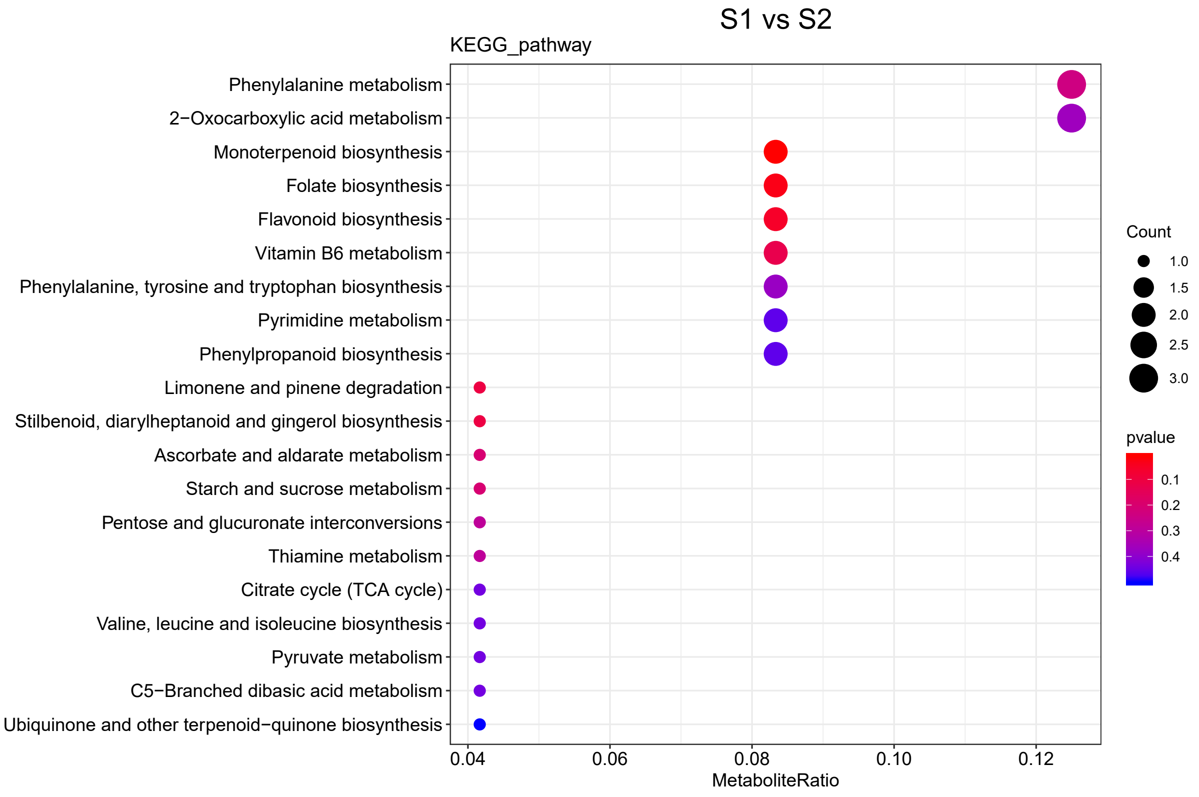
S1 vs S2
KEGG_pathway
Phenylalanine metabolism
2−Oxocarboxylic acid metabolism
Monoterpenoid biosynthesis
Folate biosynthesis
Flavonoid biosynthesis
Vitamin B6 metabolism
Phenylalanine, tyrosine and tryptophan biosynthesis
Pyrimidine metabolism
Phenylpropanoid biosynthesis
Limonene and pinene degradation
Stilbenoid, diarylheptanoid and gingerol biosynthesis
Ascorbate and aldarate metabolism
Starch and sucrose metabolism
Pentose and glucuronate interconversions
Thiamine metabolism
Citrate cycle (TCA cycle)
Valine, leucine and isoleucine biosynthesis
Pyruvate metabolism
C5−Branched dibasic acid metabolism
Ubiquinone and other terpenoid−quinone biosynthesis
0.04
0.06
0.08
0.10
0.12
MetaboliteRatio
Count
1.0
1.5
2.0
2.5
3.0
pvalue
0.1
0.2
0.3
0.4

(B)

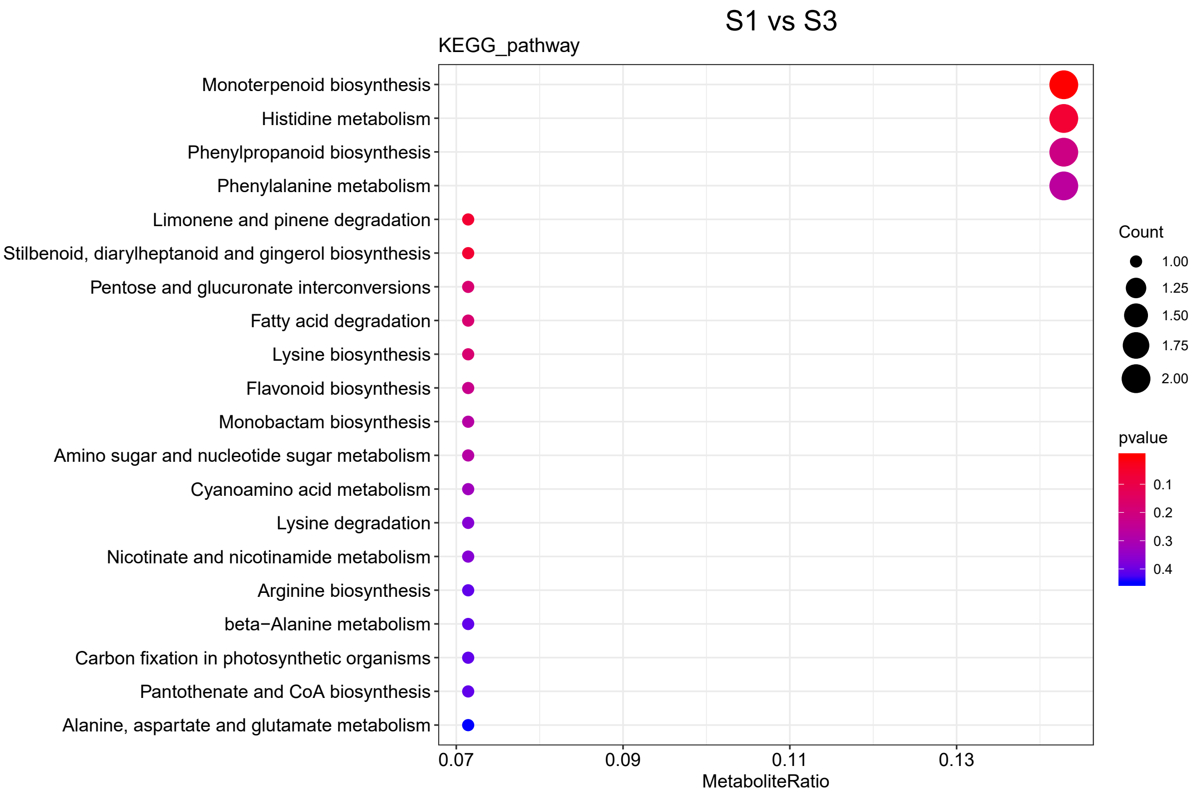
S1 vs S3
KEGG_pathway
Monoterpenoid biosynthesis
Histidine metabolism
Phenylpropanoid biosynthesis
Phenylalanine metabolism
Limonene and pinene degradation
Stilbenoid, diarylheptanoid and gingerol biosynthesis
Pentose and glucuronate interconversions
Fatty acid degradation
Lysine biosynthesis
Flavonoid biosynthesis
Monobactam biosynthesis
Amino sugar and nucleotide sugar metabolism
Cyanoamino acid metabolism
Lysine degradation
Nicotinate and nicotinamide metabolism
Arginine biosynthesis
beta−Alanine metabolism
Carbon fixation in photosynthetic organisms
Pantothenate and CoA biosynthesis
Alanine, aspartate and glutamate metabolism
0.07
0.09
0.11
0.13
MetaboliteRatio
Count
1.00
1.25
1.50
1.75
2.00
pvalue
0.1
0.2
0.3
0.4

(C)

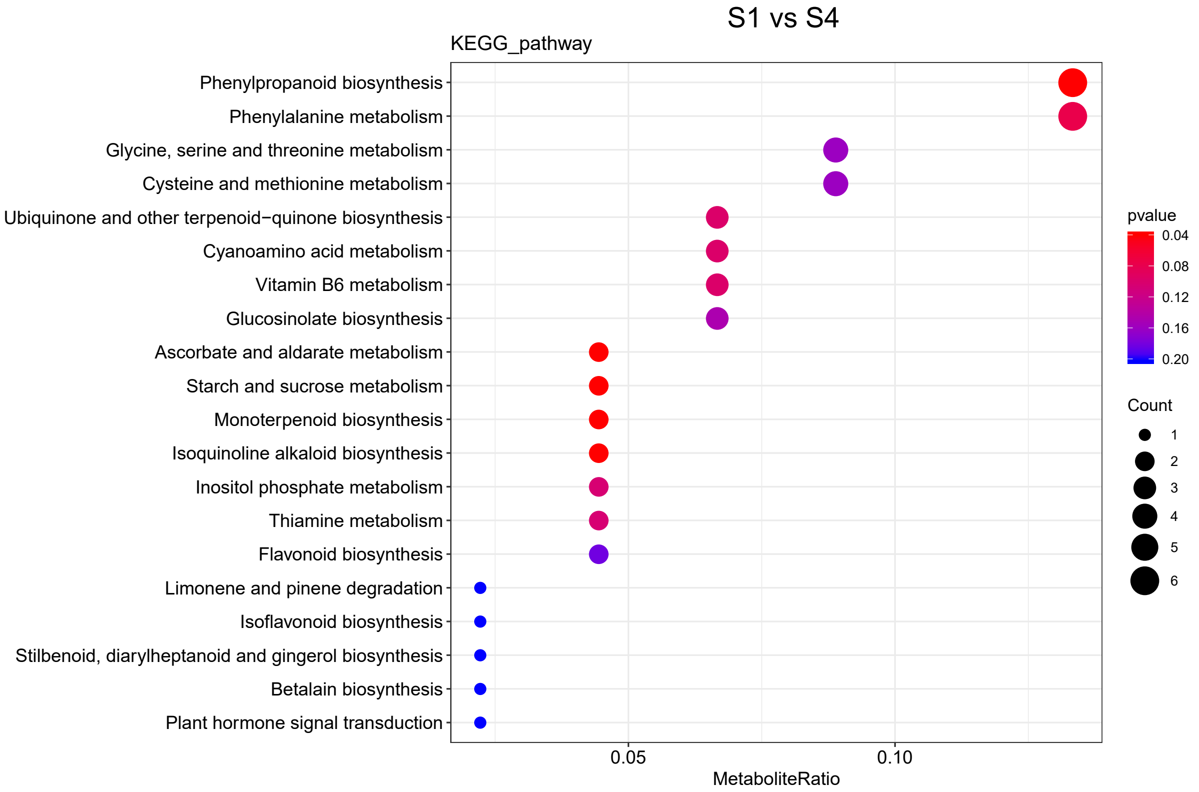
S1 vs S4
KEGG_pathway
Phenylpropanoid biosynthesis
Phenylalanine metabolism
Glycine, serine and threonine metabolism
Cysteine and methionine metabolism
Ubiquinone and other terpenoid−quinone biosynthesis
Cyanoamino acid metabolism
Vitamin B6 metabolism
Glucosinolate biosynthesis
Ascorbate and aldarate metabolism
Starch and sucrose metabolism
Monoterpenoid biosynthesis
Isoquinoline alkaloid biosynthesis
Inositol phosphate metabolism
Thiamine metabolism
Flavonoid biosynthesis
Limonene and pinene degradation
Isoflavonoid biosynthesis
Stilbenoid, diarylheptanoid and gingerol biosynthesis
Betalain biosynthesis
Plant hormone signal transduction
0.05
0.10
MetaboliteRatio
pvalue
0.04
0.08
0.12
0.16
0.20
Count
1
2
3
4
5
6

(D)

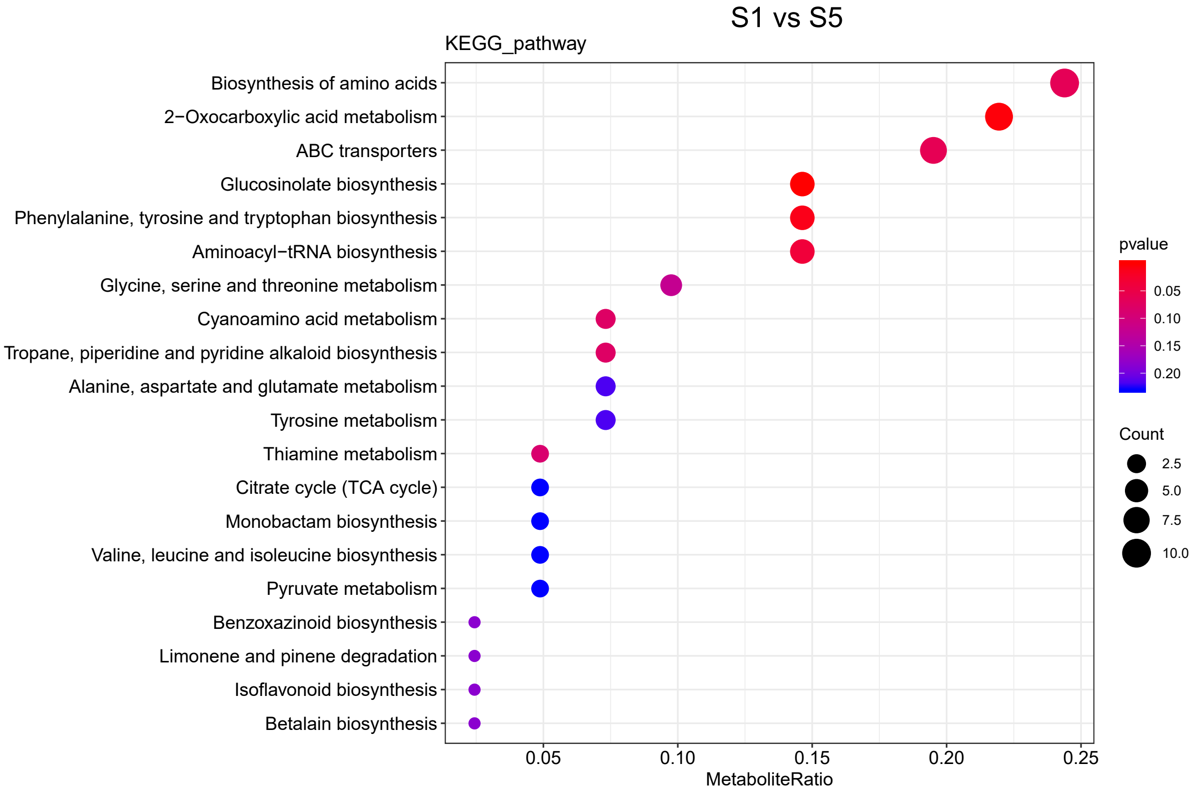
S1 vs S5
KEGG_pathway
Biosynthesis of amino acids
2−Oxocarboxylic acid metabolism
ABC transporters
Glucosinolate biosynthesis
Phenylalanine, tyrosine and tryptophan biosynthesis
Aminoacyl−tRNA biosynthesis
Glycine, serine and threonine metabolism
Cyanoamino acid metabolism
Tropane, piperidine and pyridine alkaloid biosynthesis
Alanine, aspartate and glutamate metabolism
Tyrosine metabolism
Thiamine metabolism
Citrate cycle (TCA cycle)
Monobactam biosynthesis
Valine, leucine and isoleucine biosynthesis
Pyruvate metabolism
Benzoxazinoid biosynthesis
Limonene and pinene degradation
Isoflavonoid biosynthesis
Betalain biosynthesis
0.05
0.10
0.15
0.20
0.25
MetaboliteRatio
pvalue
0.05
0.10
0.15
0.20
Count
2.5
5.0
7.5
10.0

(E)

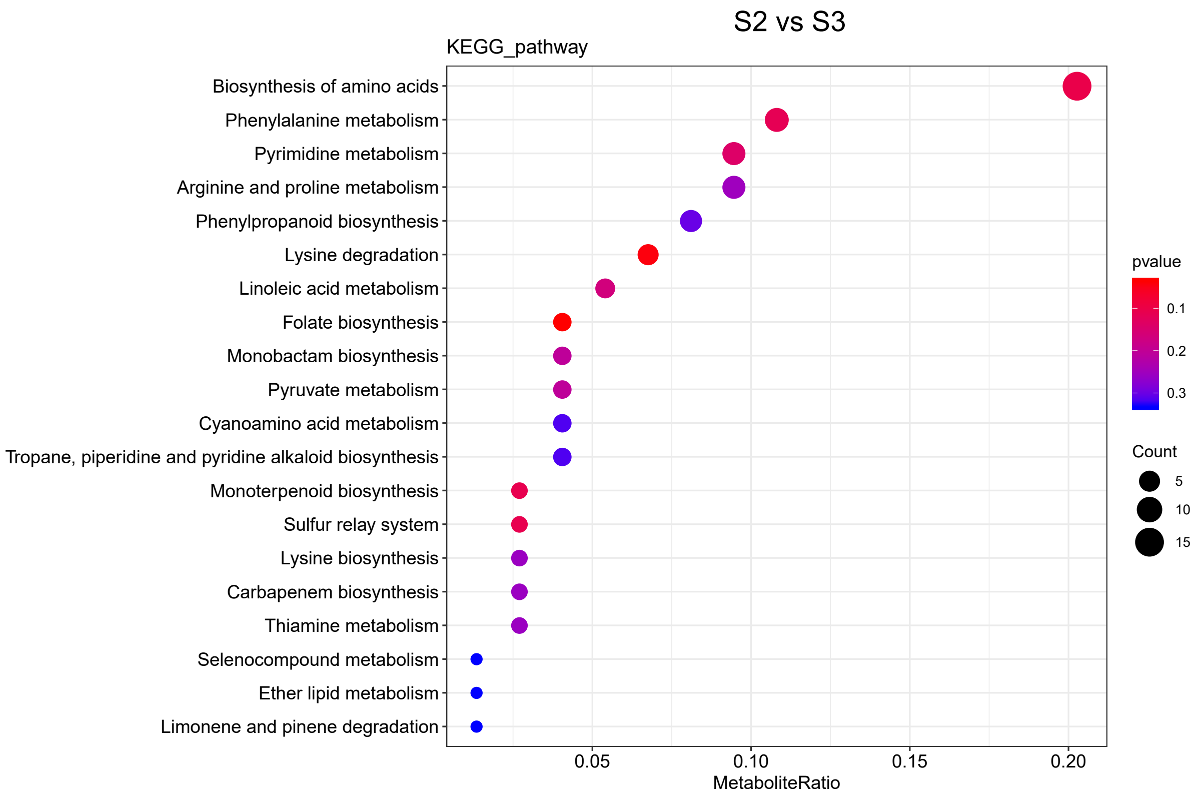
S2 vs S3
KEGG_pathway
Biosynthesis of amino acids
Phenylalanine metabolism
Pyrimidine metabolism
Arginine and proline metabolism
Phenylpropanoid biosynthesis
Lysine degradation
Linoleic acid metabolism
Folate biosynthesis
Monobactam biosynthesis
Pyruvate metabolism
Cyanoamino acid metabolism
Tropane, piperidine and pyridine alkaloid biosynthesis
Monoterpenoid biosynthesis
Sulfur relay system
Lysine biosynthesis
Carbapenem biosynthesis
Thiamine metabolism
Selenocompound metabolism
Ether lipid metabolism
Limonene and pinene degradation
0.05
0.10
0.15
0.20
MetaboliteRatio
pvalue
0.1
0.2
0.3
Count
5
10
15

(F)

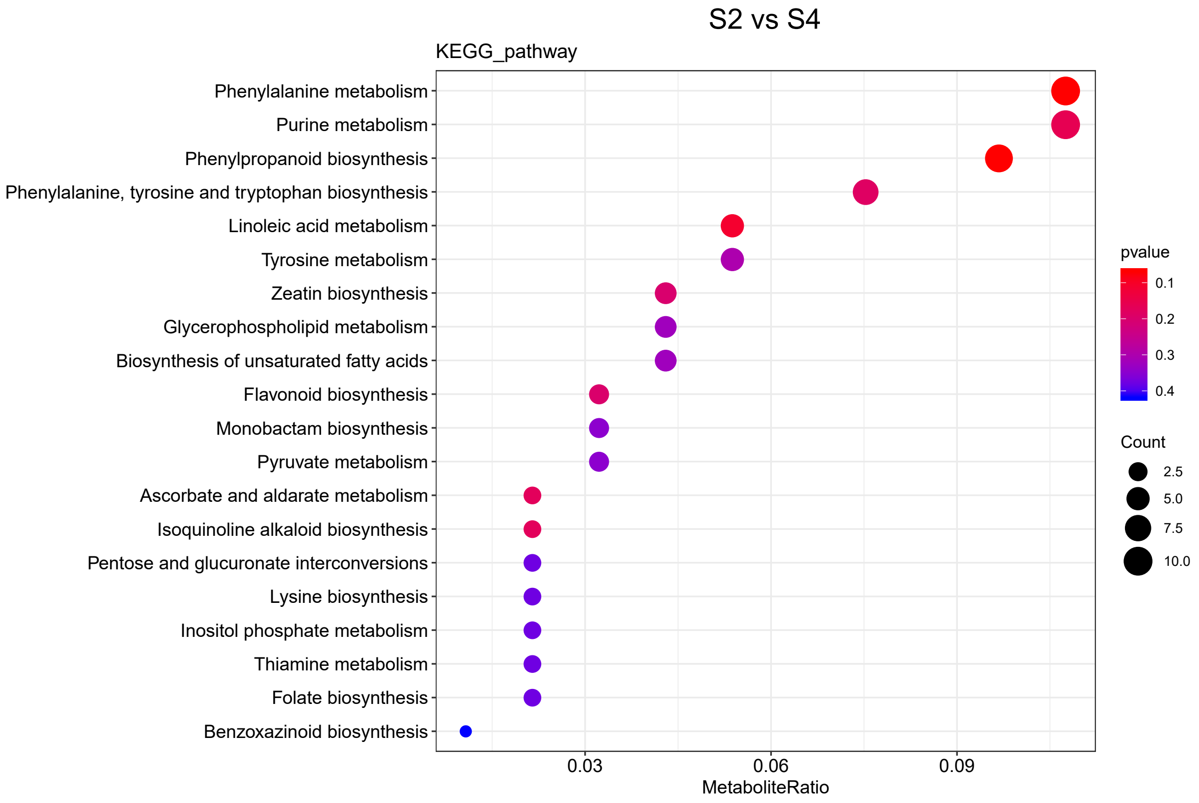
S2 vs S4
KEGG_pathway
Phenylalanine metabolism
Purine metabolism
Phenylpropanoid biosynthesis
Phenylalanine, tyrosine and tryptophan biosynthesis
Linoleic acid metabolism
Tyrosine metabolism
Zeatin biosynthesis
Glycerophospholipid metabolism
Biosynthesis of unsaturated fatty acids
Flavonoid biosynthesis
Monobactam biosynthesis
Pyruvate metabolism
Ascorbate and aldarate metabolism
Isoquinoline alkaloid biosynthesis
Pentose and glucuronate interconversions
Lysine biosynthesis
Inositol phosphate metabolism
Thiamine metabolism
Folate biosynthesis
Benzoxazinoid biosynthesis
0.03
0.06
0.09
MetaboliteRatio
pvalue
0.1
0.2
0.3
0.4
Count
2.5
5.0
7.5
10.0

(G)

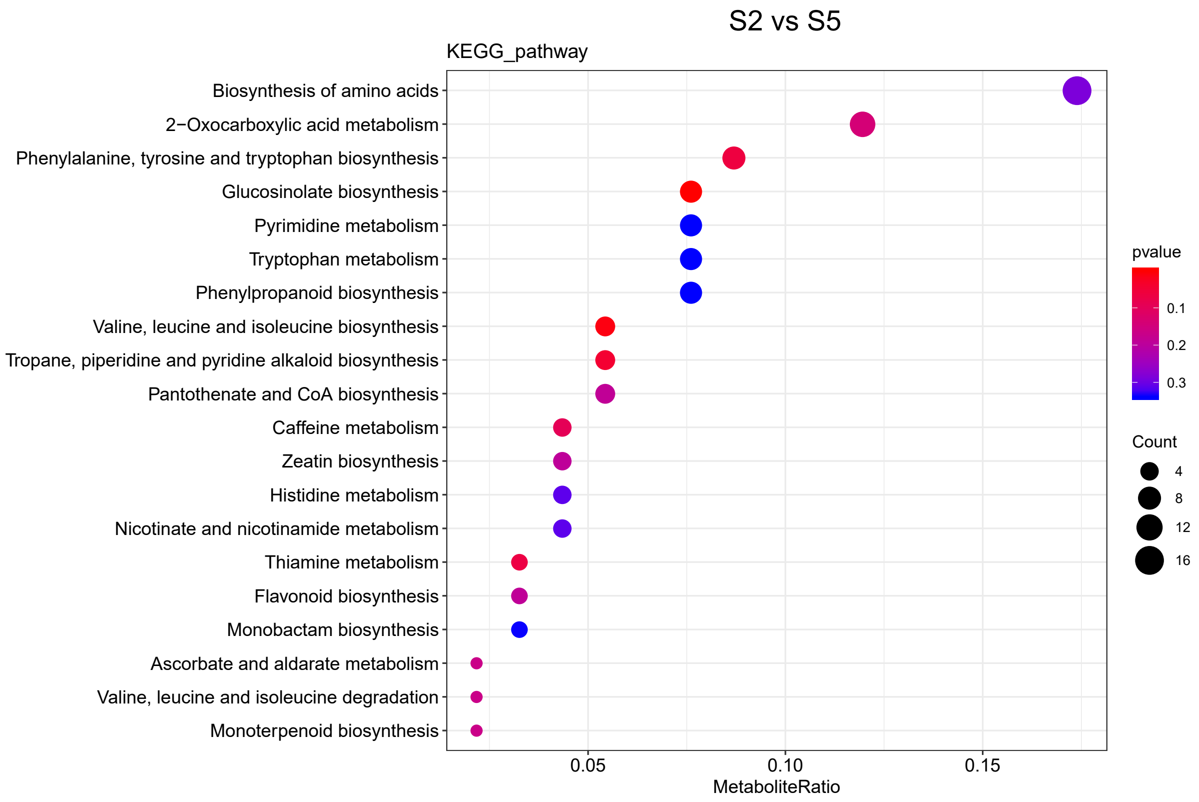
S2 vs S5
KEGG_pathway
Biosynthesis of amino acids
2−Oxocarboxylic acid metabolism
Phenylalanine, tyrosine and tryptophan biosynthesis
Glucosinolate biosynthesis
Pyrimidine metabolism
Tryptophan metabolism
Phenylpropanoid biosynthesis
Valine, leucine and isoleucine biosynthesis
Tropane, piperidine and pyridine alkaloid biosynthesis
Pantothenate and CoA biosynthesis
Caffeine metabolism
Zeatin biosynthesis
Histidine metabolism
Nicotinate and nicotinamide metabolism
Thiamine metabolism
Flavonoid biosynthesis
Monobactam biosynthesis
Ascorbate and aldarate metabolism
Valine, leucine and isoleucine degradation
Monoterpenoid biosynthesis
0.05
0.10
0.15
MetaboliteRatio
pvalue
0.1
0.2
0.3
Count
4
8
12
16

(H)

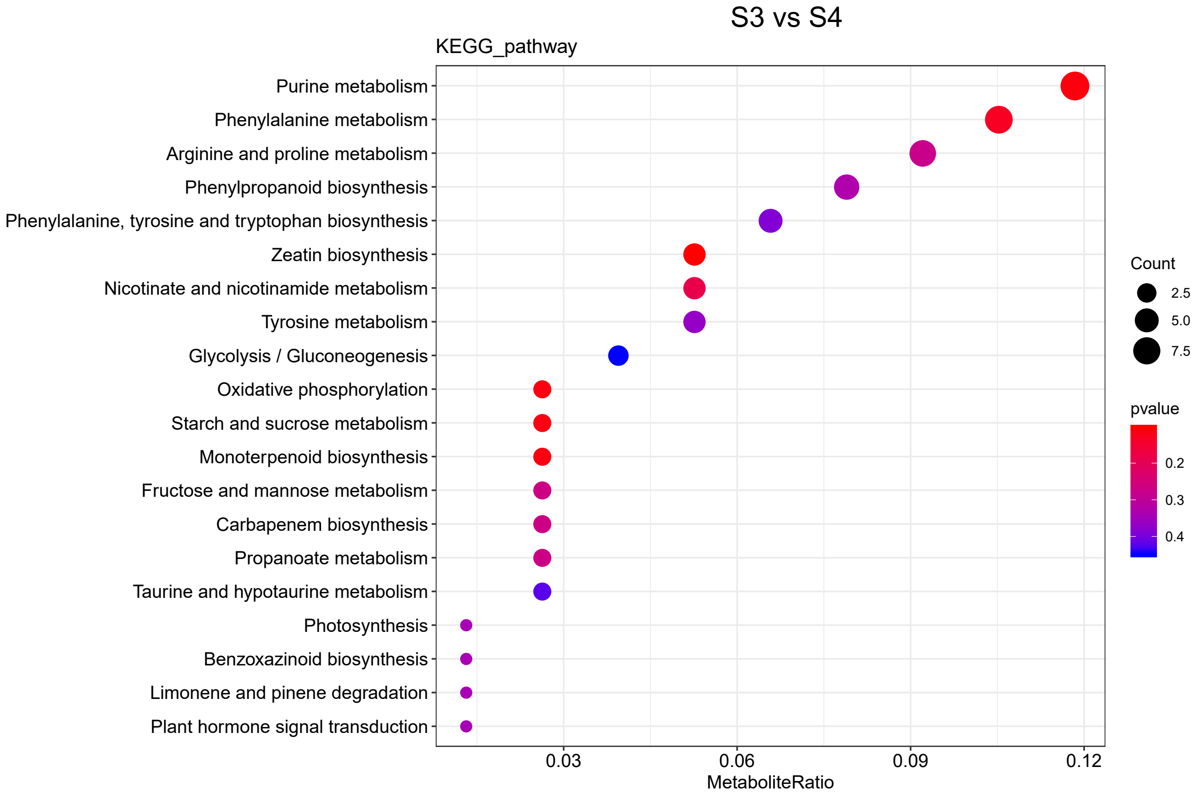
S3 vs S4
KEGG_pathway
Purine metabolism
Phenylalanine metabolism
Arginine and proline metabolism
Phenylpropanoid biosynthesis
Phenylalanine, tyrosine and tryptophan biosynthesis
Zeatin biosynthesis
Nicotinate and nicotinamide metabolism
Tyrosine metabolism
Glycolysis / Gluconeogenesis
Oxidative phosphorylation
Starch and sucrose metabolism
Monoterpenoid biosynthesis
Fructose and mannose metabolism
Carbapenem biosynthesis
Propanoate metabolism
Taurine and hypotaurine metabolism
Photosynthesis
Benzoxazinoid biosynthesis
Limonene and pinene degradation
Plant hormone signal transduction
0.03
0.06
0.09
0.12
MetaboliteRatio
Count
2.5
5.0
7.5
pvalue
0.2
0.3
0.4

(I)

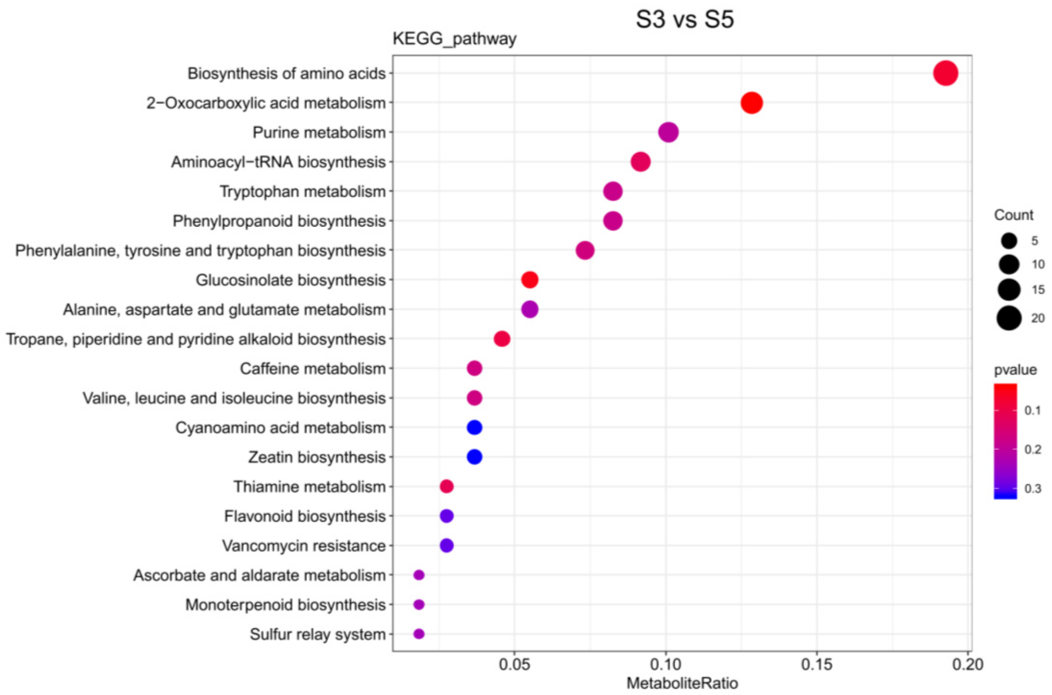

(J)

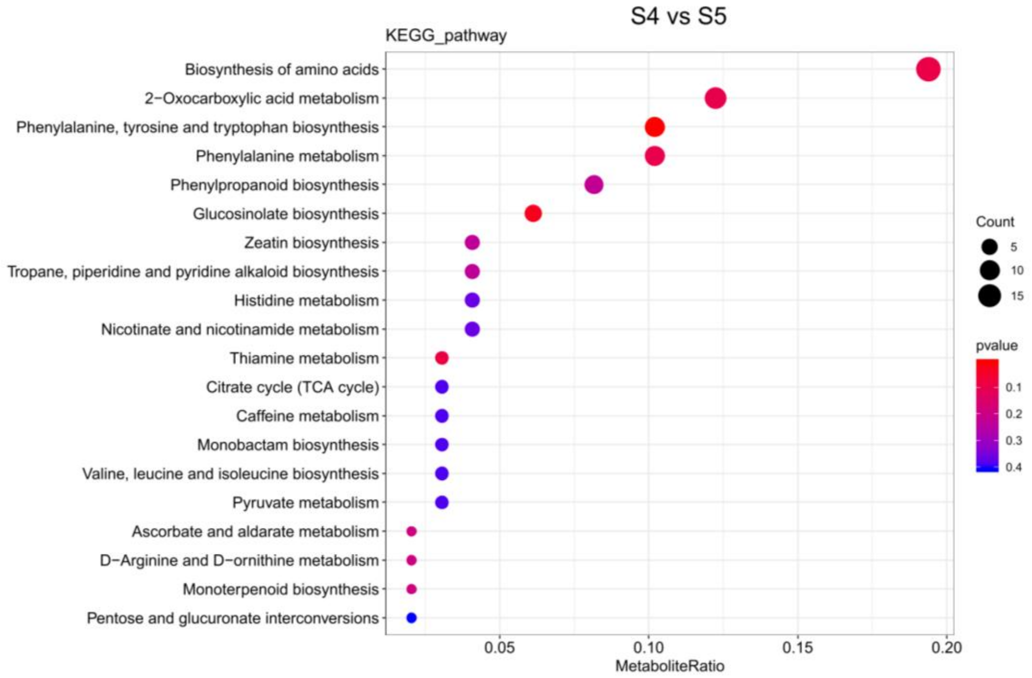

Supplementary Figure 7. KEGG pathway enrichment analysis of DAMs among different group comparisons. (A) S1 vs S2, (B) S1 vs S3, (C) S1 vs S4, (D) S1 vs S5, (E) S2 vs S3, (F) S2 vs S4, (G) S2 vs S5, (H) S3 vs S4, (I) S3 vs S5, and (J) S4 vs S5.

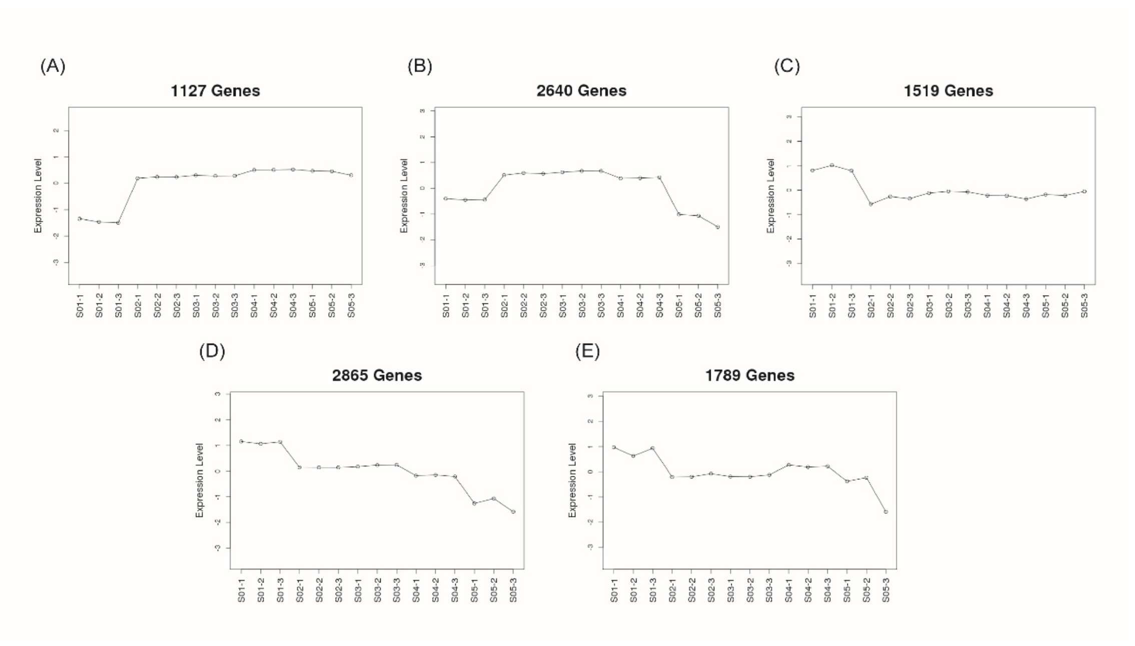

Supplementary Figure 8. All DEGs were classified into 5 clusters (A-E) according to the expression trend of five time points during seed development.

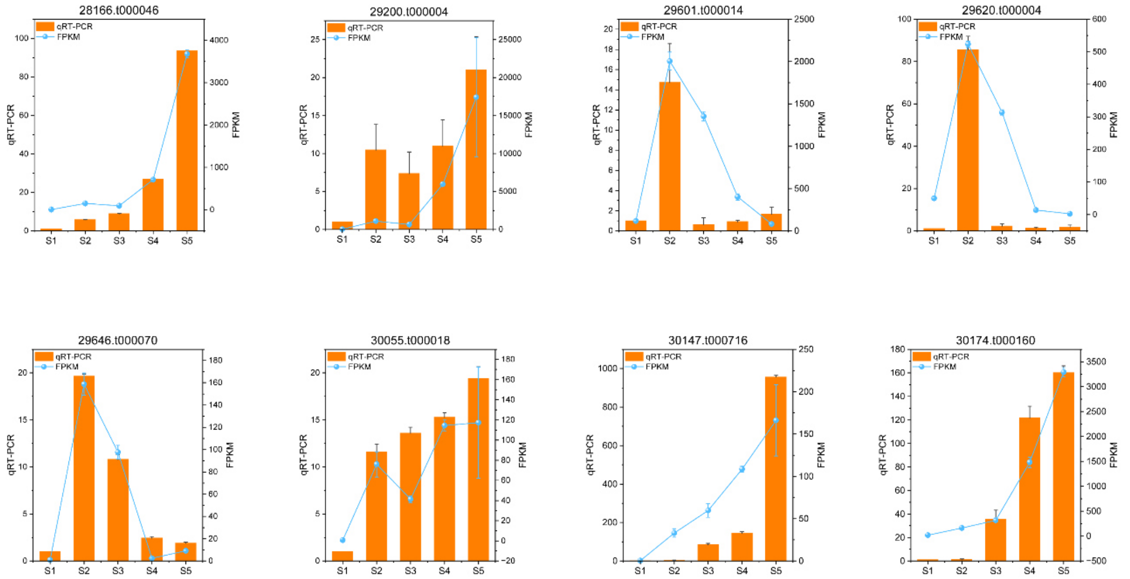

Supplementary Figure 9. The expression changes of randomly selected twelve common genes confirmed by RT-qPCR analysis to verify transcriptome data. The comparative $\log_2$FPKM and $\Delta\Delta$Ct values at stage S1 were used as the control for normalization. Means ± SDs, n = 3 biological replicates.

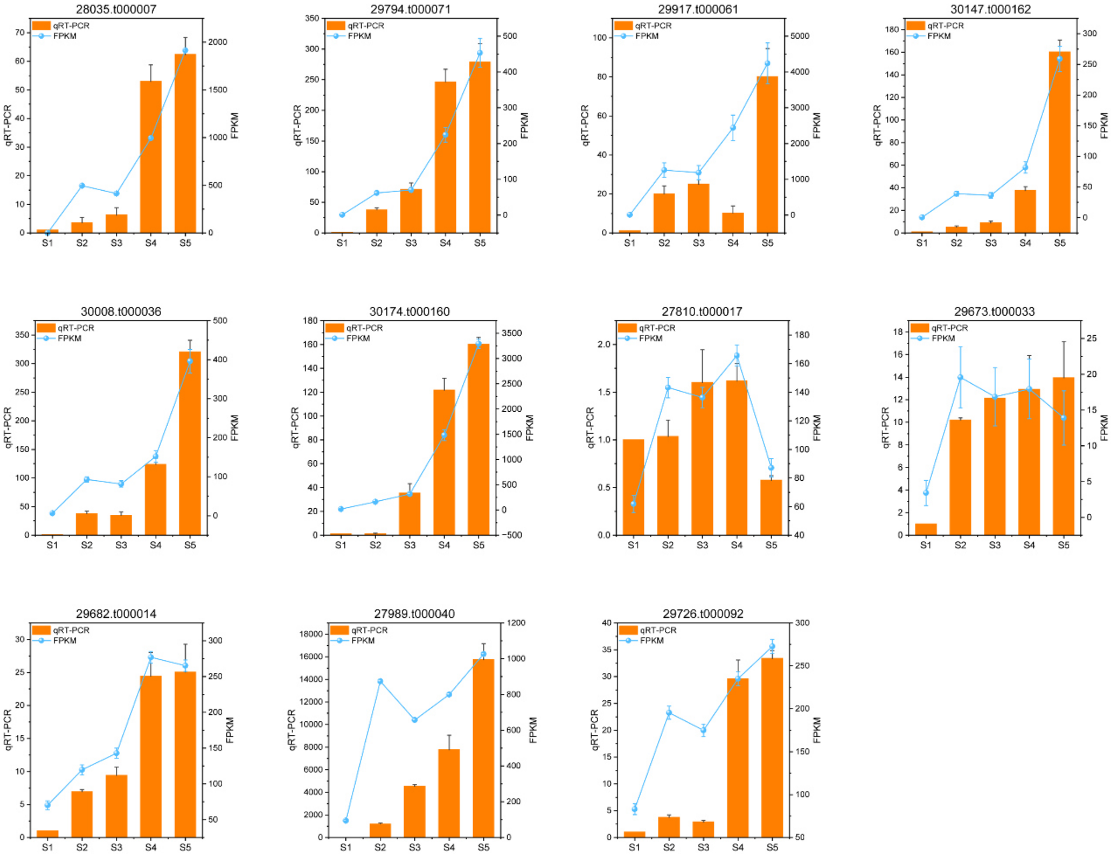

Supplementary Figure 10. The expression changes of randomly selected 6 DEGs belong to Cluster I confirmed by RT-qPCR analysis to verify transcriptome data. The comparative log2FPKM and ΔΔCt values at stage S1 were used as the control for normalization. Means ± SDs, n = 3 biological replicates.

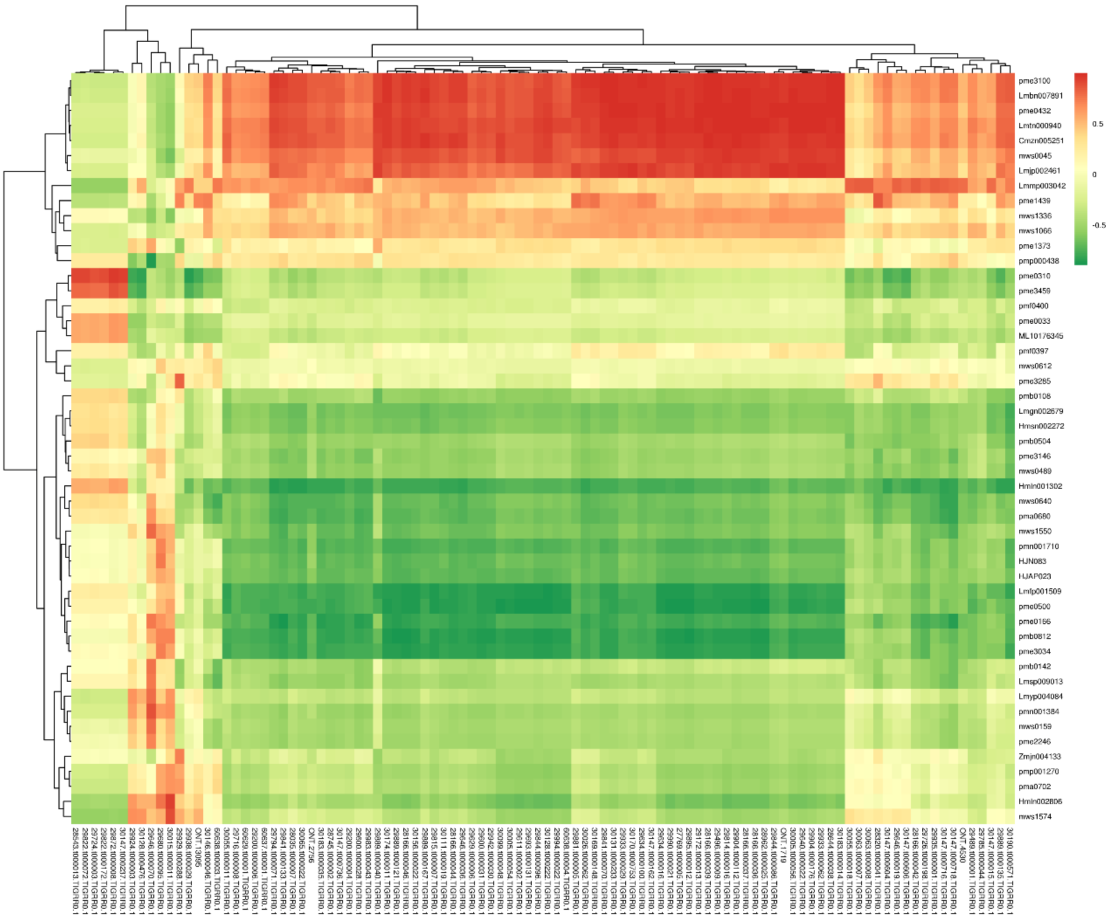

Supplementary Figure 11. Correlations were calculated between the top 100 transcripts and top 50 metabolites based on variable importance (VIP) values using O2PLS analysis. Red indicates a strong positive correlation between metabolites and transcripts, while green indicates a strong negative correlation.
